# Iodide/H_2_O_2_ Catalyzed Intramolecular Oxidative Amination for the Synthesis of 3,2′-Pyrrolidinyl Spirooxindoles

**DOI:** 10.3390/molecules23092265

**Published:** 2018-09-05

**Authors:** Yu-Ting Gao, Xiao-Yang Jin, Qi Liu, An-Di Liu, Liang Cheng, Dong Wang, Li Liu

**Affiliations:** 1 Beijing National Laboratory for Molecular Sciences (BNLMS), CAS Key Laboratory of Molecular Recognition and Function, CAS Research/Education Center for Excellence in Molecular Sciences, Institute of Chemistry, Chinese Academy of Sciences, Beijing 100190, China; gaoyuting@iccas.ac.cn (Y.-T.G.); jinxy123@iccas.ac.cn (X.-Y.J.); liuqir@iccas.ac.cn (Q.L.); liuandi@iccas.ac.cn (A.-D.L.); dwang210@iccas.ac.cn (D.W.); 2 University of Chinese Academy of Sciences, Beijing 100049, China

**Keywords:** spirooxindoles, iodine/H_2_O_2_, oxidative amination

## Abstract

An ammonium iodide/hydrogen peroxide-mediated intramolecular oxidative amination of 3-aminoalkyl-2-oxindoles was achieved, affording the corresponding 3,2′-pyrrolidinyl spirooxindoles and their 6- or 7-membered analogous in moderate to high yields. This metal-free procedure features very mild reaction conditions, non-toxicity and easily handled hydrogen peroxide as a clean oxidant.

## 1. Introduction

**Figure 1:**
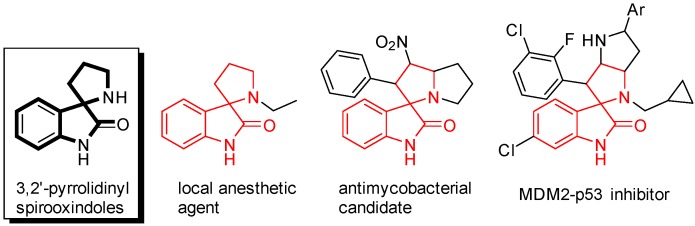
Biologically active 3,2′-pyrrolidinyl spirooxindole derivatives

The 3,2′-pyrrolidinyl spirooxindoles and their 6- or 7- membered analogous are among one of the most important privileged structural units, whicH-Not only frequently appear in a plethora of biologically active oxindole alkaloids, but also in several pharmaceuticals [1,2,3,4,5,6,7,8,9,10,11,12,13]. As one of the most common such skeletons, 3,2′-pyrrolidinyl spirooxindoles shown a wide spectrum of notable bioactivities [14,15,16,17], such as local anesthetic and antimycobacterial effects, and binding to the MDM2 protein to interrupt its protein-protein interaction with TP53 (Figure 1).

**Scheme 1:**
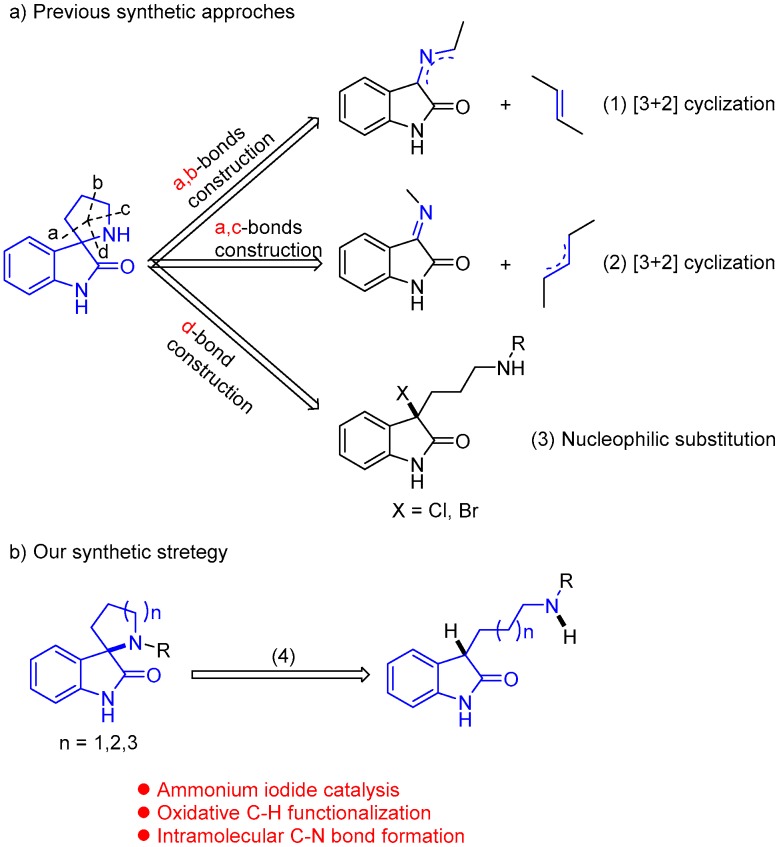
Strategies for the 3,2′-pyrrolidinyl spirooxindole synthesis.

Considering their potential pharmaceutical value, decades of research have introduced several synthetic methods to assemble this type of spirooxindole compounds. However, almost all published approaches are based on the powerful (3 + 2) cyclization to construct the five-membered pyrrolidinyl ring [18,19,20,21,22,23,24,25,26] (Scheme 1). For example, metal-catalyzed and organocatalytic cycloadditions of azomethine imines derived from isatins with a variety of dipolarophiles have been successfully developed (*a,b-bond construction*, pathway (1)). Alternatively, a nucleophilic attack of 1,3-ylides on isatin-3-imine derivatives also led to the formation of corresponding spirooxindoles (*a,c-bond construction*, pathway (2)). Despite the fact that these elegant one-step assemblies are capable of producing various spirooxindoles with molecular complexity and diversity, efficient and accessible methodologies towards these synthetic targets are still in high demand. The first intramolecular nucleophilic substitution of 3-halo-2-oxindoles providing a 3,2′-pyrrolidinyl-spirooxindole intermediate was reported by Cohen et al. almost 30 years ago (*d-bond construction*, pathway (3)) [27]. Unfortunately, almost no further development has been reported in the field of intramolecular amination. Very recently, Chen et al. described an annulation reaction of 3-bromo-2-oxindoles generating spirocyclic oxindoles, in which a cinchona alkaloid catalyzed amination was involved [27]. Thus, the potential application of simpler 3-*H*-2-oxindole precursors, which would provide the spirooxindole derivatives via a challenging intramolecular oxidative amination approach (pathway (4)), has not succeeded [28,29].

On the other hand, oxidative C–H/N–H coupling is a direct approach for the effective construction of C-N bonds [30,31,32,33,34,35]. However, most of the existing methods typically require the use of transition metals, which has hindered their practical application [30,31,32,33,34,35]. Therefore, the development of new C-N bond formation reactions under metal-free conditions is also in high demand [36,37,38,39,40,41]. Recently, the catalytic system with I^−^ or I_2_ and a terminal oxidant has emerged as an environmentally benign oxidative system for a range of transformations, such as etherification, lactonization, and others [42,43,44,45,46,47,48,49,50,51]. Based on our continuing interest in the synthesis of 2-oxindole derivatives and the application of iodine/iodide catalysis [52,53,54,55,56,57], we herein report our progress in the TBAI/H_2_O_2_ catalyzed intramolecular oxidative amination of 3-aminoalkyl 2-oxindoles to give 3,2′-pyrrolidinyl-spirooxindoles and their 6/7-membered analogs [58,59,60,61,62].

## 2. Results and Discussion

To explore the possibility of the proposed intramolecular C-N bond formation process, our investigation began with a screening of several iodides to evaluate their catalytic activity under different reaction conditions (Table 1). The model reaction of (3-(benzylamino)propyl)-2-oxindole (**1a**) was firstly performed with the commonly used oxidant TBHP (entries 1–3). While the desired cyclization product 1′-benzylspiro[indoline-3,2′-pyrrolidin]-2-one (**2a**) could be obtained, only moderate yields were observed. To our delight, when H_2_O_2_ was used as the terminal oxidant, a slightly better result (62% yield) was obtained with TBAI as the optimal iodide source in CH_3_CN (entries 4–5). Subsequently, a survey of other solvents was carried out (entries 6–9). The results indicated that changing the solvent has a significant effect on the reaction. Among the solvents screened, toluene emerged as the most suitable medium in terms of high chemical yield (79%) and short reaction time (30 min) (entry 9).

**Scheme 2:**
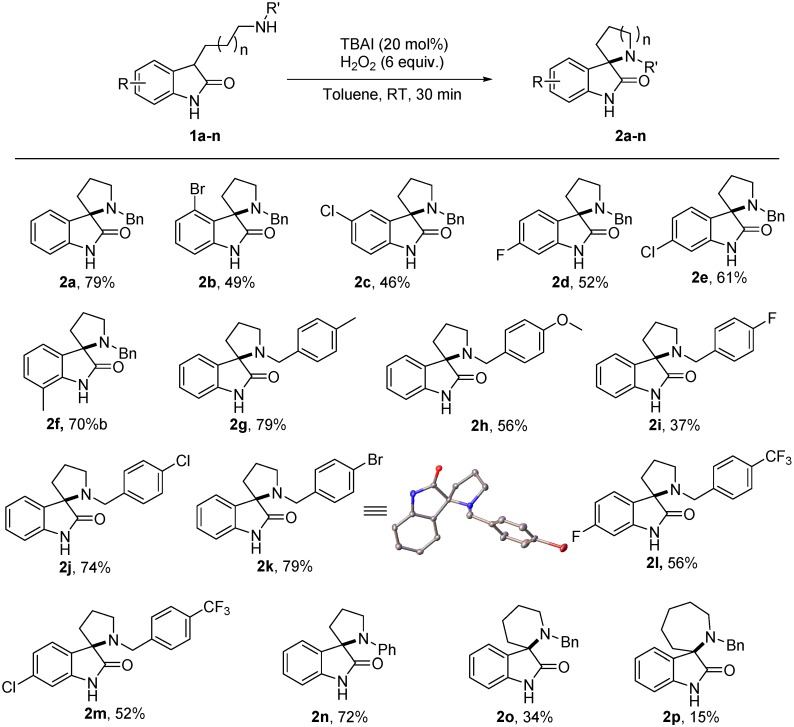
Investigations on the substrate scope.

The results with different substrates under the optimized conditions that probed the scope of this transformation are summarized in Scheme 2. A variety of substituted 3-aminopropanyl- 2-oxindoles **1b**–**n**, including those bearing electron-withdrawing (F, Cl, Br, CF_3_) and electron-donating (CH_3_) substituents on the oxindole ring were examined. Gratifyingly, all of these substrates afforded the desired cyclization products **2b**–**n** in good to high yields. It is noteworthy that the substituents on the amino group did not much influence the yield of the reaction and a good yield of **2n** was obtained when an *N*-Ph substrate was used. However, for 2-oxindoles with longer aminoalkyl chains (compounds **2o**–**p**), diminished yields were observed for the 6-membered and 7-membered spirooxindoles, respectively. The configuration of products was also confirmed by the X-ray crystallographic analysis of product **2k**.

**Scheme 3:**
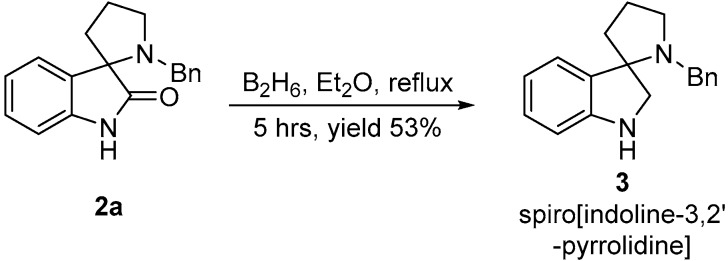
Selective reduction of the cyclization product.

In order to demonstrate the synthetic utility of this methodology, we next performed the selective reduction of the amide by using borane. Spiro[indoline-3,2′-pyrrolidine] **3**, a core structure found in several natural products and pharmaceutical agents [63], was obtained in 53% yield (Scheme 3).

**Scheme 4:**
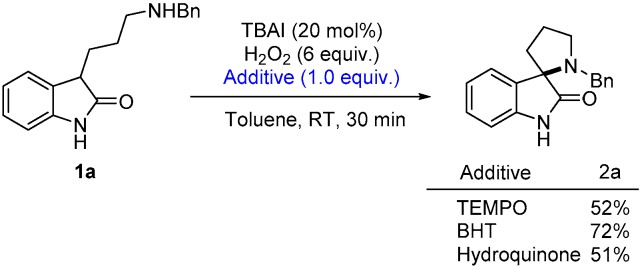
Control experiments.

Some control experiments were conducted in order to elucidate the mechanism. Previous reports [58,59,60,61,62] have suggested that a radical process was involved in iodide/oxidant catalyzed C-N bond formation. However, when a stoichiometric amount of radical inhibitors, like TEMPO, BHT and hydroquinone, was used, the cyclization of **1a** proceeded smoothly and afforded the desired product **2a** in comparable yields (Scheme 4), indicating a complete different pathway.

**Scheme 5:**
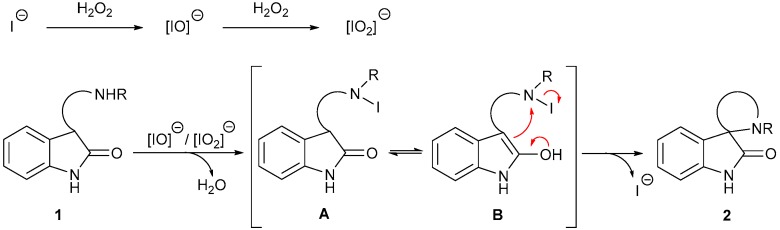
The proposed mechanism.

On the basis of the experimental results and recent studies [64,65,66], a possible stepwise mechanism is proposed (Scheme 5). First, two hypervalent iodine species, i.e., IO^–^ and IO_2_^−^ were likely generated by the oxidation of iodide with H_2_O_2_. Those hypervalent iodines then reacted with aminoalkyl 2-oxindoles **1** to form an iodoamino intermediate **A**, which was in equilibrium with its enolate **B**. The latter readily underwent an intramolecular substitution to afford the cyclization product **2**, while releasing the iodide, which further underwent oxidation to regenerate the reactive hypervalent iodine species.

**Scheme 6:**
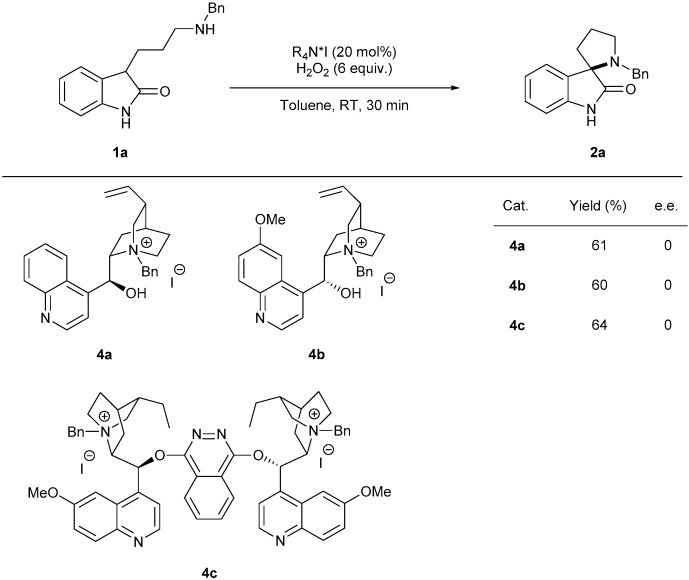
Preliminary experiments applying chiral iodide catalysts.

We had an initial predisposition toward using chiral iodide salts because of their easy preparation from enantiopure amines and well-known role as phase-transfer catalysts in asymmetric transformations [67,68,69]. Other catalytic use, particularly for asymmetric C-N bond formations, has been quite limited. Thus we prepared several cinchona alkaloid-based iodide salts **4a**–**c** as chiral quaternary ammonium iodide salts, and tested their stereocontrol in this reaction with hydrogen peroxide as an environmentally benign oxidant (Scheme 6).

All those tested catalysts gave good results, with an average yield of 60%, however, no enantioselectivity was observed. Thus, a more detailed screening of other quaternary ammonium iodide salts may be needed for this transformation.

## 3. Materials and Methods 

### 3.1. Chemicals and Instruments

Unless otherwise noted, all reagents were obtained from commercially suppliers and were used without further purification. All reactions were carried out under argon atmosphere using Schlenk techniques. Oxindoles **1** were obtained from commercially suppliers or prepared according to the literature procedures. TBAI were obtained from commercially suppliers. TLC analysis was performed on glass-baked silica plates and visualized with UV light. Column chromatography was performed on silica gel (200–300 mesh) using petroleum ether/ethyl acetate/ dichloromethane/methanol. ^1^H-, and ^13^C-NMR spectra were obtained on Bruker 300 MHz, 400 MHz or 500 MHz NMR spectrometer in the deuterated solvents indicated (Bruker, Billerica, MA, USA). Chemical shifts are reported in ppm from tetramethylsilane with the solvent resonance as the internal standard. The following abbreviations were used to designate chemical shift multiplicities: s = singlet, d = doublet, t = triplet, q = quartet, h = heptet, m = multiplet. All first-order splitting patterns were assigned on the basis of the appearance of the multiplet. Splitting patterns that could not be easily interpreted are designated as multiplet (m) or (br). Melting points were measured without correction on a Beijing Tech X-4 apparatus (Beijing Tech Instrument Co., Ltd., Beijing, China). IR spectra were recorded on a Nicolet 6700 FT-IR spectrometer (Thermo Fisher Scientific, Waltham, MA, USA). HRMS were obtained using electrospray ionization (ESI) mass spectrometer (Exactive, Thermo Fisher Scientific, Waltham, MA, USA). 

### 3.2. Synthetic Procedures

#### 3.2.1. General Procedure for Synthesis of **1**

To a mixture of indolyl propionic acid [64] (10.0 mmol, 1.9 g) and triethylamine (20.0 mmol, 2.8 mL) in dichloromethane (70 mL) was added 1-[bis(dimethylamino)methylene]-1*H*-1,2,3-triazolo [4,5-*b*]pyridinium 3-oxid hexafluorophosphate (12.0 mmol, 4.6 g) and benzylamine (12.0 mmol, 1.3 mL). The mixture was stirred at room temperature for 1 h and then diluted with dichloromethane (200 mL). The organic layer was washed by water (200 mL × 2), dried over anhydrous sodium sulfate and evaporated to afford the intermediate *N*-benzyl-3-(1*H*-indol-3-yl)propanamide without further purification. *N*-Benzyl-3-(1*H*-indol-3-yl)propanamide (8.0 mmol, 2.3 g) was dissolved in dry tetrahydrofuran (40 mL) under argon, and then a solution of lithium aluminum hydride (32.0 mmol, 12.8 mL, 2.5 M in THF) was added dropwise. The mixture was heated to reflux overnight and then cooled to room temperature. To the vigorously stirring mixture were added H_2_O (4 mL), 15% NaOH (4 mL), H_2_O (4 mL × 3) at 0 °C. After being stirred at 0 °C for another 10 min, the mixture was filtered through celite, the white filter cake was washed with methanol and the filtrate was concentrated *in vacuum*. The crude was purified by silica column chromatography (elute: dichloromethane /methanol 10/1, with 1% NH_4_OH) to afford the intermediate *N*-benzyl-3-(1*H*-indol-3-yl)- propan-1-amine[70] as a yellow oil. To the solution of *N*-benzyl-3-(1*H*-indol-3-yl)propan-1-amine (6.9 mmol,1.8 g) in dimethyl sulfoxide (20.7 mmol, 1.5 mL) and methanol (0.3 mL) was added concentrated hydrochloric acid (20.7 mmol, 1.7 mL) slowly at 0 °C. The resulting mixture was stirred at 50 °C for 5 h. After cooling to room temperature, the mixture was diluted with ethyl acetate (50 mL) and washed with H_2_O (50 mL). Aqueous phase was adjust to pH = 7 by ammonium hydroxide and extracted with ethyl acetate (50 mL × 2). The organic was dried over anhydrous sodium sulfate, evaporated and purified by silica column chromatography (elute: dichloromethane /methanol 10/1, with 1% NH_4_OH) to afford the desired product 3-(3-(benzylamino)propyl)indolin-2-one (**1a**) [71]. See the Supplementary Materials for the details. 

#### 3.2.2. General Procedure for the Synthesis of Compounds **2**

To the mixture of oxindole **1** (0.10 mmol) and TBAI (20 mol%) in toluene (0.5 mL) was added 35% H_2_O_2_ (6 equiv.), the reaction mixture was stirred at room temperature until completion the reaction. After that time, the mixture was quenched by saturated sodium thiosulfate solution (1 mL) and diluted with dichloromethane (10 mL). The organic layer was washed by water (10 mL × 2), dried over anhydrous sodium sulfate and evaporated to afford the crude product. The crude was purified by silica column chromatography (elute: petroleum ether/ethyl acetate 2/1) to give the pure desired products **2**.

### 3.3. Characterization Data

*3-(3-(Benzylamino)propyl)indolin-2-one* (**1a**): Yellow oil. ^1^H-NMR (300 MHz, CDCl_3_) δ 8.47 (br, 1H), 7.31–7.30 (m, 4H), 7.24–7.16 (m, 3H), 7.03–6.98 (t, *J* = 7.5, 1H), 6.86–6.84 (d, *J* = 7.5 Hz, 1H), 3.78 (s, 2H), 3.49–3.45 (t, *J* = 6.0 Hz, 1H), 2.68–2.63 (m, 2H), 2.49 (br, 1H), 2.05–1.98 (dd, *J* = 14.1, 8.1 Hz, 2H), 1.66–1.53 (m, 2H). ^13^C-NMR (126 MHz, DMSO-*d*_6_) δ 178.7, 142.7, 136.2, 129.4, 129.0, 128.3, 127.7, 127.6, 124.0, 121.2, 109.2, 51.3, 47.3, 44.7, 27.2, 23.6. IR ν_max_ (KBr, film, cm^−1^): 3203, 3061, 2929, 2856, 1683, 1471, 751. HRMS (ESI): calcd for C_18_H_21_ON_2_^+^ [M + H]^+^: 281.1648, found: 281.1647.

*3-(3-(benzylamino)propyl)-4-bromoindolin-2-one* (**1b**): Pink solid, m.p. 83–85 °C. ^1^H-NMR (300 MHz, DMSO-*d*_6_) δ 10.70 (s, 1H), 8.56 (br, 1H), 7.43–7.38 (m, 5H), 7.16–7.14 (d, *J* = 5.4 Hz, 2H), 6.86–6.84 (d, *J* = 3.9 Hz, 1H), 4.01 (s, 2H), 3.60 (s, 1H), 2.84–2.79 (t, *J* = 7.8 Hz, 2H), 2.21–2.18 (m, 1H), 2.02–1.98 (m, 1H), 1.42–1.37 (m, 2H). ^13^C-NMR (126 MHz, DMSO-*d*_6_) δ 177.3, 144.8, 133.0, 130.0, 129.8, 128.54, 128.49, 127.8, 124.6, 118.4, 108.7, 50.2, 46.5, 46.2, 24.6, 21.4. IR ν_max_ (KBr, film, cm^−1^): 3360, 2920, 2848, 1698, 1458, 1019, 699. HRMS (ESI): calcd for C_18_H_20_ON_2_Br^+^ [M + H]^+^: 359.0754, found: 359.0750.

*3-(3-(benzylamino)propyl)-5-chloroindolin-2-one* (**1c**): Orange solid, m.p. 89–91 °C. ^1^H-NMR (500 MHz, DMSO-*d*_6_) δ 10.59 (s, 1H), 8.81 (br, 1H), 7.51–7.48 (m, 2H), 7.39–7.36 (m, 4H), 7.24–7.21 (t, *J* = 8.0 Hz, 1H), 6.85–6.82 (m,1H), 4.02 (s, 2H), 3.53–3.51 (t, *J* = 5.5 Hz, 1H), 2.83–2.80 (m, 2H), 1.89–1.85 (m, 2H), 1.60–1.58 (m, 2H). ^13^C-NMR (126 MHz, DMSO-*d*_6_) δ 178.2, 141.7, 133.0, 131.5, 129.8, 128.6, 128.5, 127.5, 125.4, 124.3, 110.6, 50.3, 46.5, 44.8, 26.6, 22.2. IR ν_max_ (KBr, film, cm^−1^): 3446, 2920, 2849, 1702, 1478, 699. HRMS (ESI): calcd for C_18_H_20_ON_2_Cl^+^ [M + H]^+^: 315.1259, found: 315.1257. 

*3-(3-(Benzylamino)propyl)-6-fluoroindolin-2-one* (**1d**): Pink solid, m.p. 81–83 °C. ^1^H-NMR (500 MHz, DMSO-*d*_6_) δ 10.62 (s, 1H), 9.05 (br, 1H), 7.52–7.51 (m, 2H), 7.40 (m, 1H), 7.39–7.38 (m, 2H), 7.30–7.26 (m, 1H), 6.77–6.74 (m, 1H), 6.66–6.64 (m, 1H), 4.04 (s, 2H), 3.47–3.45 (t, *J* = 5.5 Hz, 1H), 2.85–2.82 (t, *J* = 6.5 Hz, 2H), 1.91–1.82 (m, 2H), 1.64–1.63 (m, 2H). ^13^C-NMR (126 MHz, DMSO-*d*_6_) δ 178.9, 163.0, 161.0, 144.3, 144.2, 132.5, 129.9, 128.7, 128.5, 125.33, 125.26, 125.02, 125.00, 107.3, 107.1, 97.5, 97.3, 50.1, 46.4, 44.0, 26.9, 22.0. ^19^F NMR (377 MHz, DMSO-*d*_6_) −113.9(s). IR ν_max_ (KBr, film, cm^−1^): 3359, 3195, 2920, 2849, 1702, 1469, 1340. HRMS (ESI): calcd for C_18_H_20_ON_2_F^+^ [M + H]^+^: 299.1554, found: 299.1554.

*3-(3-(Benzylamino)propyl)-6-chloroindolin-2-one* (**1e**): Orange solid, m.p. 89–91 °C. ^1^H-NMR (300 MHz, DMSO-*d*_6_) δ 10.64 (s, 1H), 9.07 (br, 1H), 7.53–7.50 (m, 2H), 7.43–7.38 (m, 3H), 7.30–7.27 (m, 1H), 7.01–6.87 (dd, *J* = 7.8, 1.5 Hz, 1H), 6.87–6.86 (d, *J* = 1.5 Hz, 1H), 4.04 (s, 2H), 3.50–3.47 (t, *J* = 5.7 Hz, 1H), 2.85–2.80 (t, *J* = 7.8 Hz, 2H), 1.91–1.81 (m, 2H), 1.65–1.63 (m, 2H). ^13^C-NMR (75 MHz, DMSO) δ 178.4, 144.3, 132.5, 131.9, 129.8, 128.6, 128.5, 128.1, 125.5, 120.8, 109.3, 50.0, 46.3, 44.1, 26.7, 21.9. IR ν_max_ (KBr, film, cm^−1^): 3360, 3188, 2920, 2848, 1703, 1486, 749. HRMS (ESI): calcd for C_18_H_20_ON_2_Cl^+^ [M + H]^+^: 315.1259, found: 315.1258.

*3-(3-(Benzylamino)propyl)-7-methylindolin-2-one* (**1f**): White solid, m.p. 202–204 °C. ^1^H-NMR (300 MHz, DMSO-*d*_6_) δ 10.45 (s, 1H), 9.00 (br, 1H), 7.51–7.48 (m, 2H), 7.45–7.40(m, 3H), 7.10–7.07 (d, *J* = 7.2 Hz, 1H), 7.01–6.98 (d, *J* = 7.8 Hz, 1H), 6.90–6.85 (t, *J* = 7.5 Hz, 1H), 4.07 (s, 2H), 3.49–3.48 (t, *J* = 5.4 Hz, 1H), 2.90–2.85 (m, 2H), 2.19 (s, 3H), 1.91–1.82 (m, 2H), 1.68–1.61 (m, 2H). ^13^C-NMR (126 MHz, DMSO-*d*_6_) δ 179.0, 141.3, 132.2, 129.9, 129.0, 128.81, 128.78, 128.6, 121.3, 118.5, 50.1, 46.5, 44.8, 26.8, 22.0, 16.5. IR ν_max_ (KBr, film, cm^−1^): 3392, 2946, 2838, 1702, 1458, 694. HRMS (ESI): calcd for C_19_H_23_ON_2_^+^ [M + H]^+^: 295.1805, found: 295.1804.

*3-(3-((4-Methylbenzyl)amino)propyl)indolin-2-one* (**1g**): Yellow oil. ^1^H-NMR (300 MHz, CDCl_3_) δ 8.04 (br, 1H), 7.22–7.10 (m, 6H), 7.03–6.98 (t, *J* = 7.2 Hz, 1H), 6.85–6.83 (m, 1H), 3.71 (s, 2H), 3.49–3.46 (t, *J* = 6.0 Hz, 1H), 2.65–2.60 (t, *J* = 7.2 Hz, 2H), 2.32 (s, 3H), 2.02–1.97 (m, 2H), 1.72 (br, 1H), 1.64–1.52 (m, 2H). ^13^C-NMR (126 MHz, CDCl_3_) δ 180.2, 141.6, 136.9, 136.5, 129.5, 129.1, 128.1, 127.8, 124.1, 122.2, 109.6, 53.5, 48.9, 45.7, 28.1, 26.0, 21.1. IR ν_max_ (KBr, film, cm^−1^): 3204, 3022, 2923, 2857, 1706, 1620, 1486, 751. HRMS (ESI): calcd for C_19_H_23_ON_2_^+^ [M + H]^+^: 295.1805, found: 295.1804.

*3-(3-((4-Methoxybenzyl)amino)propyl)indolin-2-one* (**1h**): Yellow oil. ^1^H-NMR (300 MHz, DMSO-*d*_6_) δ 10.34 (br, 1H), 7.23–7.13 (m, 4H), 6.96–6.91 (t, *J* = 7.5 Hz, 1H), 6.85–6.81 (m, 3H), 3.71 (s, 3H), 3.56 (s, 2H), 3.42–3.38 (t, *J* = 5.7 Hz, 1H), 2.97 (br, 1H), 2.46–2.41(t, *J* = 7.2 Hz, 2H), 1.89–1.79 (m, 2H), 1.43–1.37 (m, 2H). ^13^C-NMR (126 MHz, DMSO-*d*_6_) δ 178.9, 158.0, 142.8, 132.6, 129.7, 129.1, 127.5, 123.9, 121.2, 113.4, 109.1, 54.9, 52.2, 48.3, 45.0, 27.7, 25.5. IR ν_max_ (KBr, film, cm^−1^): 3197, 2933, 2835, 1698, 1471, 1177, 751. HRMS (ESI): calcd for C_19_H_23_O_2_N_2_^+^ [M + H]^+^: 311.1765, found: 311.1747.

*3-(3-((4-Fluorobenzyl)amino)propyl)indolin-2-one* (**1i**): Yellow solid, m.p. 101–103 °C ^1^H-NMR (300 MHz, DMSO-*d*_6_) δ 10.42 (s, 1H), 8.86 (br, 1H), 7.57–7.52 (m, 2H), 7.28–7.15 (m, 4H), 6.98–6.93 (t, *J* = 7.5 Hz, 1H), 6.84–6.82 (d, *J* = 7.8 Hz, 1H), 4.05 (s, 2H), 3.50–3.48 (t, *J* = 5.7 Hz, 1H), 2.87–2.82 (t, *J* = 7.8 Hz, 2H), 1.89–1.82 (m, 2H), 1.67–1.59 (m, 2H). ^13^C-NMR (101 MHz, DMSO-*d*_6_) δ 178.5, 163.4, 161.0, 142.7, 132.3, 132.3, 129.2, 128.6, 127.6, 124.0, 121.2, 115.4, 115.2, 109.2, 49.1, 46.2, 44.5, 26.9, 22.0. ^19^F NMR (377 MHz, DMSO-*d*_6_) δ −113.9(s). IR ν_max_ (KBr, film, cm^−1^): 3361, 2920, 2849, 1703, 1471, 1226, 751. HRMS (ESI): calcd for C_18_H_20_ON_2_F^+^ [M + H]^+^: 299.1554, found: 299.1553.

*3-(3-((4-Chlorobenzyl)amino)propyl)indolin-2-one* (**1j**): Yellow oil. ^1^H-NMR (300 MHz, CDCl_3_) δ 8.76 (br, 1H), 7.31–7.28 (m, 1H), 7.25–7.17(m, 5H), 7.03–6.98 (t, *J* = 7.5 Hz, 1H), 6.87–6.84 (d, *J* = 7.8 Hz, 1H), 3.72 (s, 2H), 3.49–3.45 (t, *J* = 5.7 Hz, 1H), 2.64–2.59 (t, *J* = 7.2 Hz, 2H), 2.24–2.19 (m, 1H), 2.04–1.97 (dd, *J* = 14.1, 7.8 Hz, 2H), 1.62–1.51 (m, 2H). ^13^C-NMR (126 MHz, CDCl_3_) δ 180.2, 141.6, 138.3, 132.7, 129.5, 129.4, 128.5, 127.9, 124.1, 122.3, 109.7, 53.0, 48.7, 45.7, 28.0, 25.9. IR ν_max_ (KBr, film, cm^−1^): 3200, 2932, 1714, 1471, 1015, 751. HRMS (ESI): calcd for C_18_H_20_ON_2_Cl^+^ [M + H]^+^: 315.1259, found: 315.1256.

*3-(3-((4-Bromobenzyl)amino)propyl)indolin-2-one* (**1k**): Yellow solid, m.p. 102–104 °C. ^1^H-NMR (300 MHz, DMSO-*d*_6_) δ 10.41 (s, 1H), 8.33 (br, 1H), 7.60–7.58 (d, *J* = 8.1 Hz, 2H), 7.44–7.41 (d, *J* = 8.1 Hz, 2H), 7.27–7.24 (d, *J* = 7.2 Hz, 1H), 7.20–7.15 (t, *J* = 7.8 Hz, 1H), 6.97–6.92 (t, *J* = 7.5 Hz, 1H), 6.84–6.81 (d, *J* = 7.8 Hz, 1H), 3.96 (s, 2H), 3.48–3.44 (t, *J* = 5.7 Hz, 1H), 2.80–2.74 (t, *J* = 7.5 Hz, 2H), 1.91–1.80 (m, 2H), 1.64–1.55 (m, 2H). ^13^C-NMR (75 MHz, DMSO-*d*_6_) δ 178.6, 142.7, 131.8, 131.3, 129.3, 127.7, 124.0, 121.6, 121.2, 109.2, 49.8, 46.8, 44.7, 26.9, 22.7. IR ν_max_ (KBr, film, cm^−1^): 3366, 3197, 2922, 2850, 1702, 1622, 1471, 753. HRMS (ESI): calcd for C_18_H_20_ON_2_Br^+^ [M + H]^+^: 359.0754, found: 359.0740.

*6-Fluoro-3-(3-((4-(trifluoromethyl)benzyl)amino)propyl)indolin-2-one* (**1l**): Orange oil. ^1^H-NMR (300 MHz, DMSO-*d*_6_) δ 10.56 (br, 1H), 7.69–7.66 (d, *J* = 7.8 Hz, 2H), 7.58–7.55 (d, *J* = 7.8 Hz, 2H), 7.30–7.25 (m, 1H), 6.81–6.67 (m, 2H), 3.77 (s, 2H), 3.48–3.44 (t, *J* = 6.3Hz, 1H), 2.56 (s, 1H), 1.95–1.87 (m, 3H), 1.49–1.41 (m, 3H). ^13^C-NMR (126 MHz, DMSO-*d*_6_) δ 179.8, 163.3, 161.4, 146.5, 144.8, 144.7, 128.9, 125.9, 125.6, 125.5, 125.30, 125.27, 107.7, 107.5, 97.9, 97.7, 52.8, 49.0, 45.0, 28.1, 26.0. ^19^F NMR (377 MHz, DMSO-*d*_6_) δ −60.86(s), −113.93 (s). IR ν_max_ (KBr, film, cm^−1^): 3633, 2952, 2855, 1717, 1558, 1329, 1020, 849, 737. HRMS (ESI): calcd for C_19_H_19_ON_2_F_4_^+^ [M + H]^+^: 367.1428, found: 367.1422.

*6-Chloro-3-(3-((4-(trifluoromethyl)benzyl)amino)propyl)indolin-2-one* (**1m**): Orange oil. ^1^H-NMR (300 MHz, DMSO-*d*_6_) δ 10.57 (br, 1H), 7.71–7.68 (d, *J* = 7.8 Hz, 2H), 7.58–7.56 (d, *J* = 7.8 Hz, 2H), 7.30–7.28 (m, 1H), 7.05–7.02 (m, 1H), 6.89 (s, 1H), 3.77 (s, 2H), 3.52–3.48 (t, *J* = 5.7 Hz, 1H), 2.57 (s, 1H), 1.97–1.86 (m, 3H), 1.50–1.38 (m, 3H). ^13^C-NMR (126 MHz, DMSO-*d*_6_) δ 178.9, 146.1, 144.3, 131.8, 128.6, 128.4, 125.3, 124.83, 124.80, 120.8, 109.2, 52.3, 48.5, 44.6, 27.5, 25.5. ^19^F NMR (377 MHz, DMSO-*d*_6_) δ −60.79 (s). IR ν_max_ (KBr, film, cm^−1^): 3419, 3181, 2952, 2800, 1704, 1619, 1326, 1127, 1068, 737. HRMS (ESI): calcd for C_19_H_19_ON_2_ClF_3_^+^ [M + H]^+^: 382.1133, found: 383.1126.

*3-(3-(Phenylamino)propyl)indolin-2-one* (**1n**): Pale yellow solid, m.p. 105–107 °C. ^1^H-NMR (500 MHz, CDCl_3_) δ 8.87 (s, 1H), 7.24–7.22 (m, 2H), 7.17–7.14 (t, *J* = 7.5 Hz, 2H), 7.06–7.03 (t, *J* = 7.5 Hz, 1H), 6.93–6.91 (d, *J* = 8.5 Hz, 1H), 6.70–6.67 (m, 1H), 6.57–6.56 (d, *J* = 8.5 Hz, 2H), 3.67 (br, 1H), 3.56–3.54 (t, *J* = 5.5 Hz, 2H), 3.14–3.11 (t, *J* = 7.0 Hz, 2H), 2.13–2.09 (m, 2H), 1.77–1.66 (m, 2H). ^13^C-NMR (126 MHz, CDCl_3_) δ 180.4, 148.2, 141.6, 129.3, 129.2, 128.0, 124.0, 122.4, 117.2, 112.7, 109.8, 45.7, 43.7, 27.9, 25.7. IR ν_max_ (KBr, film, cm^−1^): 3368, 3210, 2925, 2855, 1707, 1602, 1471, 749. HRMS (ESI): calcd for C_17_H_19_ON_2_^+^ [M + H]^+^: 267.1492, found: 267.1494.

*3-(4-(Benzylamino)butyl)indolin-2-one* (**1o**): Pale yellow solid, m.p. 172–173 °C. ^1^H-NMR (300 MHz, DMSO-*d*_6_) δ 10.41 (s, 1H), 8.66 (br, 1H), 7.52–7.50 (m, 2H), 7.38–7.36 (m, 3H), 7.26–7.24 (d, *J* = 7.5 Hz, 1H),7.19–7.14 (t, *J* = 7.5 Hz, 1H), 6.96–6.91 (t, *J* = 7.2 Hz, 1H), 6.84–6.82 (d, *J* = 7.5 Hz, 1H), 3.99 (s, 2H), 3.43–3.39 (m, 1H), 2.77–2.71 (t, *J* = 7.5 Hz, 2H), 1.85–1.76 (m, 2H), 1.65–1.60 (m, 2H), 1.32–1.24 (m, 2H). ^13^C-NMR (101 MHz, DMSO-*d*_6_) δ 178.7, 142.7, 133.3, 129.7, 129.5, 128.42, 128.38, 127.5, 123.9, 121.1, 109.1, 50.2, 46.4, 44.9, 29.5, 25.8, 22.6. IR ν_max_ (KBr, film, cm^−1^): 3359, 2920, 2849, 1702, 1472, 751. HRMS (ESI): calcd for C_19_H_23_ON_2_^+^ [M + H]^+^: 295.1805, found: 295.1802.

*3-(5-(Benzylamino)pentyl)indolin-2-one* (**1p**): Yellow oil. ^1^H-NMR (300 MHz, CDCl_3_) δ 8.47 (br, 1H), 7.34–7.28 (m, 5H), 7.24–7.20 (m, 2H), 7.05–7.00 (m, 1H), 6.90–6.88 (d, *J* = 8.1 Hz, 1H), 3.80 (s, 2H), 3.49–3.45 (t, *J* = 6.0 Hz, 1H), 2.65–2.60 (t, *J* = 6.9 Hz, 2H), 2.22 (br, 1H), 2.00–1.95 (m, 2H), 1.55–1.48 (m, 2H), 1.45–1.36 (m, 4H). ^13^C-NMR (126 MHz, CDCl_3_) δ 180.2, 141.5, 139.7, 129.7, 128.4, 128.2, 127.8, 127.0, 124.1, 122.2, 109.6, 53.8, 49.0, 45.9, 30.4, 29.5, 27.2, 25.5. IR ν_max_ (KBr, film, cm^−1^): 3197, 3061, 2830, 2856, 1683, 1506, 1471, 749. HRMS (ESI): calcd for C_20_H_25_ON_2_^+^ [M + H]^+^: 309.1961, found: 309.1959.

*1′-Benzylspiro[indoline-3,2′-pyrrolidin]-2-one* (**2a**): White solid, 21.9 mg (from 0.10 mmol), 79% yield, m.p. 154–156 °C. ^1^H-NMR (500 MHz, DMSO-*d*_6_) δ 10.28 (s, 1H), 7.35–7.33 (d, *J* = 7.5 Hz, 1H), 7.26–7.22 (m, 2H), 7.20–7.18 (m, 4H), 7.03–7.00 (t, *J* = 7.5 Hz, 1H), 6.80–6.79 (d, *J* = 7.5 Hz, 1H), 3.31–3.25 (m, 2H), 2.98–2.91 (m, 2H), 2.14–2.06 (m, 2H), 2.04–1.98 (m, 2H). ^13^C-NMR (126 MHz, DMSO-*d*_6_) δ 179.7, 142.4, 139.3, 130.7, 128.7, 128.1, 127.9, 126.8, 123.8, 121.9, 109.4, 70.8, 53.1, 50.4, 35.8, 21.7. IR ν_max_ (KBr, film, cm^−1^): 3207, 3061, 3028, 2925, 2852, 1706, 1620, 1470, 749. HRMS (ESI): calcd for C_18_H_17_ON_2_^−^ [M − H]^−^: 277.1364, found: 277.1364.

*1′-Benzyl-4-bromospiro[indoline-3,2′-pyrrolidin]-2-one* (**2b**): White solid, 17.6 mg (from 0.10 mmol), 49% yield, m.p. 169–171 °C. ^1^H-NMR (500 MHz, CDCl_3_) δ 8.74 (s, 1H), 7.32–7.31 (d, *J* = 7.5 Hz, 2H), 7.26–7.15 (m, 4H), 7.08–7.05 (m, 1H), 6.81–6.80 (dd, *J* = 7.5, 1.0 Hz, 1H), 3.59–3.49 (m, 2H), 3.17–3.10 (m, 2H), 2.68–2.63 (m, 1H), 2.25–2.18 (m, 3H). ^13^C-NMR (126 MHz, CDCl_3_) δ 181.3, 143.2, 139.5, 130.1, 128.7, 128.5, 128.0, 127.4, 126.8, 119.9, 108.9, 72.5, 53.4, 51.1, 32.8, 23.1. IR ν_max_ (KBr, film, cm^−1^): 3213, 3086, 3027, 2964, 2831, 1717, 1613, 1447, 736. HRMS (ESI): calcd for C_18_H_18_ON_2_Br^+^ [M + H]^+^: 357.0597, found: 354.0594.

*1′-Benzyl-5-chlorospiro[indoline-3,2′-pyrrolidin]-2-one* (**2c**): White solid, 14.3 mg (from 0.10 mmol), 46% yield, m.p. 149–151 °C. ^1^H-NMR (500 MHz, CDCl_3_) δ 8.47 (s, 1H), 7.34 (s, 1H), 7.25–7.19 (m, 6H), 6.79–6.78 (d, *J* = 8.0 Hz, 1H), 3.52–3.45 (m, 2H), 3.18–3.13 (m, 1H), 3.10–3.07 (m, 1H), 2.35–2.31 (m, 1H), 2.24–2.21 (m, 1H), 2.17–2.08 (m, 2H). ^13^C-NMR (126 MHz, CDCl_3_) δ 181.2, 139.5, 138.9, 133.4, 128.6, 128.5, 128.2, 128.1, 127.0, 124.6, 110.8, 71.7, 53.9, 51.4, 37.0, 22.3. IR ν_max_ (KBr, film, cm^−1^): 3213, 3063, 3029, 2963, 2840, 1717, 1619, 1475, 733. HRMS (ESI): calcd for C_18_H_18_ON_2_Br^+^ [M + H]^+^: 313.1102, found: 313.1102.

*1′-Benzyl-6-fluorospiro[indoline-3,2′-pyrrolidin]-2-one* (**2d**): White solid, 15.3 mg (from 0.10 mmol), 52% yield, m.p. 141–143 °C. ^1^H-NMR (300 MHz, CDCl_3_) δ 8.88 (s, 1H), 7.33–7.29 (m, 1H), 7.23–7.15 (m, 5H), 6.81–6.74 (m, 1H), 6.65–6.62 (dd, *J* = 14.5, 2.1 Hz, 1H), 3.52–3.41 (m, 2H), 3.22–3.06 (m, 2H), 2.35–2.04 (m, 4H). ^13^C-NMR (126 MHz, CDCl_3_) δ 182.1, 164.1, 162.2, 142.5, 142.4, 139.0, 128.5, 128.1, 126.9, 126.7, 126.6, 125.3, 125.2, 109.2, 109.0, 98.7, 98.4, 71.2, 53.8, 51.2, 36.7, 22.1. ^19^F NMR (377 MHz, CDCl_3_) δ −111.7 (s). IR ν_max_ (KBr, film, cm^−1^): 3226, 3063, 3029, 2965, 2836, 1717, 1622, 1456, 733. HRMS (ESI): calcd for C_18_H_18_ON_2_F^+^ [M + H]^+^: 297.1398, found: 297.1400.

*1′-Benzyl-6-chlorospiro[indoline-3,2′-pyrrolidin]-2-one* (**2e**): White solid, 19.0 mg (from 0.10 mmol), 61% yield, m.p. 170–172 °C. ^1^H-NMR (500 MHz, CDCl_3_) δ 8.67 (s, 1H), 7.30–7.28 (m, 1H), 7.25–7.21 (m, 2H), 7.20–7.18 (m, 3H), 7.08–7.05 (m, 1H), 6.89 (s, 1H), 3.50–3.42 (m, 2H), 3.19–3.14 (m, 1H), 3.10–3.06 (m, 1H), 2.35–2.29 (m, 1H), 2.27–2.22 (m, 1H), 2.17–2.07 (m, 2H). ^13^C-NMR (126 MHz, CDCl_3_) δ 181.6, 142.2, 139.0, 134.3, 129.8, 128.5, 128.1, 127.0, 125.2, 122.8, 110.5, 71.3, 53.9, 51.2, 36.7, 22.2. IR ν_max_ (KBr, film, cm^−1^): 3232, 3064, 3029, 2965, 2834, 1717, 1615, 1455, 732. HRMS (ESI): calcd for C_18_H_18_ON_2_Cl ^+^ [M + H]^+^: 313.1102, found: 313.1101.

*1′-Benzyl-7-methylspiro[indoline-3,2′-pyrrolidin]-2-one* (**2f**): White solid, 20.5 mg (from 0.10 mmol), 70% yield, m.p. 148–150 °C. ^1^H-NMR (500 MHz, CDCl_3_) δ 8.83 (s, 1H), 7.23–7.16 (m, 6H), 7.07–7.05 (m, 1H), 7.03–7.00 (m, 1H), 3.51–3.42 (m, 2H), 3.20–3.15 (m, 1H), 3.10–3.06 (m, 1H), 2.35–2.31 (m, 1H), 2.29 (s, 3H), 2.27–2.21 (m, 1H), 2.19–2.14 (m, 1H), 2.11–2.08 (m, 1H). ^13^C-NMR (126 MHz, CDCl_3_) δ 182.0, 139.9, 139.4, 130.9, 130.0, 128.5, 128.0, 126.8, 122.7, 121.5, 119.0, 72.0, 54.0, 51.2, 36.7, 22.2, 16.2. IR ν_max_ (KBr, film, cm^−1^): 3280, 3061, 3028, 2964, 2837, 1704, 1627, 1458, 732. HRMS (ESI): calcd for C_19_H_21_ON_2_^+^ [M + H]^+^: 293.1648, found: 293.1648.

*1′-(4-Methylbenzyl)spiro[indoline-3,2′-pyrrolidin]-2-one* (**2g**): White solid, 22.7 mg (from 0.10 mmol), 79% yield, m.p. 96–98 °C. ^1^H-NMR (500 MHz, CDCl_3_) δ 9.03 (s, 1H), 7.37–7.36 (d, *J* = 7.0 Hz, 1H), 7.23–7.20 (t, *J* = 8.0 Hz, 1H), 7.09–7.06 (m, 3H), 7.03–7.01 (m, 2H), 6.89–6.87 (d, *J* = 8.0 Hz, 1H), 3.45–3.38 (m, 2H), 3.20–3.15 (m, 1H), 3.09–3.05 (m, 1H), 2.35–2.30 (m, 1H), 2.26 (s, 3H), 2.22–2.04 (m, 3H). ^13^C-NMR (126 MHz, CDCl_3_) δ 182.0, 141.3, 136.3, 136.2, 131.4, 128.7, 128.6, 128.4, 124.1, 122.7, 109.9, 71.7, 53.5, 51.1, 36.6, 22.2, 21.0. IR ν_max_ (KBr, film, cm^−1^): 3215, 3025, 2971, 2830, 1706, 1620, 1471, 750. HRMS (ESI): calcd for C_19_H_21_ON_2_^+^ [M + H]^+^: 293.1648, found: 293.1647.

*1′-(4-Methoxybenzyl)spiro[indoline-3,2′-pyrrolidin]-2-one* (**2h**): White solid, 17.1 mg (from 0.10 mmol), 56% yield, m.p. 135–137 °C. ^1^H-NMR (500 MHz, CDCl_3_) δ 8.52 (s, 1H), 7.37–7.36 (d, *J* = 7.0 Hz, 1H), 7.24–7.21 (t, *J* = 7.5 Hz, 1H), 7.10–7.07 (m, 3H), 6.87–6.85 (d, *J* = 8.0 Hz, 1H), 6.77–6.75 (d, *J* = 8.0 Hz, 2H), 3.73 (s, 3H), 3.44–3.36 (m, 2H), 3.20–3.15 (m, 1H), 3.09–3.05 (m, 1H), 2.35–2.30 (m, 1H), 2.24–2.21 (m, 1H), 2.18–2.07 (m, 2H). ^13^C-NMR (126 MHz, CDCl_3_) δ 181.6, 158.5, 141.2, 131.5, 131.4, 129.7, 128.6, 124.2, 122.7, 113.4, 109.8, 71.5, 55.1, 53.3, 51.2, 36.7, 22.2. IR ν_max_ (KBr, film, cm^−1^): 3251, 2962, 2834, 1700, 1622, 1471, 751. HRMS (ESI): calcd for C_19_H_19_ON_2_^−^ [M − H]^−^: 307.1452, found: 307.1454.

*1′-(4-Fluorobenzyl)spiro[indoline-3,2′-pyrrolidin]-2-one* (**2i**): White solid, 10.9 mg (from 0.10 mmol), 37% yield, m.p. 126–127 °C. ^1^H-NMR (500 MHz, CDCl_3_) δ 7.89 (s, 1H), 7.36–7.35 (d, *J* = 7.0 Hz, 1H), 7.24–7.21 (td, *J* = 7.5, 1.0 Hz, 1H), 7.16–7.13 (m, 2H), 7.10–7.07 (t, *J* = 7.5 Hz, 1H), 6.92–6.88 (m, 2H), 6.84–6.82 (d, *J* = 7.5 Hz, 1H), 3.46–3.39 (m, 2H), 3.17–3.13 (m, 1H), 3.07–3.03 (m, 1H), 2.35–2.30 (m, 1H), 2.28–2.20 (m, 1H), 2.18–2.13 (m, 1H), 2.11–2.06 (m, 1H). ^13^C-NMR (126 MHz, CDCl_3_) δ 181.6, 162.8, 160.9, 141.1, 134.9, 131.3, 130.1, 130.0, 128.7, 124.1, 122.8, 114.8, 114.7, 109.9, 71.5, 53.2, 51.3, 36.7, 22.2. ^19^F NMR (377 MHz, CDCl_3_) δ −116.1 (s). IR ν_max_ (KBr, film, cm^−1^): 3213, 3086, 2964, 2836, 1717, 1622, 1471, 750. HRMS (ESI): calcd for C_18_H_18_ON_2_F^+^ [M + H]^+^: 297.1398, found: 297.1395.

*1′-(4-Chlorobenzyl)spiro[indoline-3,2′-pyrrolidin]-2-one* (**2j**): White solid, 23.3 mg (from 0.10 mmol), 74% yield, m.p. 140–142 °C. ^1^H-NMR (500 MHz, CDCl_3_) δ 8.54 (s, 1H), 7.36–7.34 (d, *J* = 7.5 Hz, 1H), 7.25–7.21 (m, 1H), 7.19–7.18 (m, 2H), 7.13–7.12 (m, 2H), 7.10–7.07 (td, *J* = 7.5, 0.5 Hz, 1H), 6.88–6.86 (d, *J* = 7.5 Hz, 1H), 3.46–3.39 (m, 2H), 3.17–3.13 (m, 1H), 3.07–3.04 (m, 1H), 2.36–2.31 (m, 1H), 2.28–2.21 (m, 1H), 2.19–2.13 (m, 1H), 2.12–2.04 (m, 1H). ^13^C-NMR (126 MHz, CDCl_3_) δ 181.5, 141.1, 137.8, 132.5, 131.2, 129.8, 128.7, 128.1, 124.1, 122.7, 109.9, 71.5, 53.2, 51.3, 36.7, 22.2. IR ν_max_ (KBr, film, cm^−1^): 3212, 2925, 2849, 1705, 1622, 1471, 750. HRMS (ESI): calcd for C_18_H_18_ON_2_Cl^+^ [M + H]^+^: 313.1102, found: 313.1099.

*1′-(4-Bromobenzyl)spiro[indoline-3,2′-pyrrolidin]-2-one* (**2k**): White solid, 19.6 mg (from 0.10 mmol), 55% yield, m.p. 140–142 °C. ^1^H-NMR (500 MHz, CDCl_3_) δ 8.36 (s, 1H), 7.35–7.33 (m, 3H), 7.24–7.21 (t, *J* = 7.5 Hz, 1H), 7.09–7.07 (m, 3H), 6.86–6.85 (d, *J* = 8.0 Hz, 1H), 3.43–3.38 (m, 2H), 3.17–3.12 (q, *J* = 7.5 Hz, 1H), 3.07–3.03 (m, 1H), 2.35–2.30 (m, 1H), 2.28–2.20 (m, 1H), 2.19–2.13 (m, 1H), 2.12–2.08 (m, 1H). ^13^C-NMR (126 MHz, CDCl_3_) δ 181.3, 141.1, 138.3, 131.2, 131.1, 130.2, 128.7, 124.2, 122.9, 120.6, 109.8, 71.5, 53.3, 51.3, 36.7, 22.3. IR ν_max_ (KBr, film, cm^−1^): 3216, 3090, 2925, 2851, 1706, 1621, 1470, 750. HRMS (ESI): calcd for C_18_H_18_ON_2_Br^+^ [M + H]^+^: 357.0597, found: 357.0594.

*6-Fluoro-1′-(4-(trifluoromethyl)benzyl)spiro[indoline-3,2′-pyrrolidin]-2-one* (**2l**): Syrup, 20.5 mg (from 0.10 mmol), 56% yield. ^1^H-NMR (300 MHz, CDCl_3_) δ 8.84 (br, 1H), 7.50–7.47 (d, *J* = 7.8 Hz, 2H), 7.33–7.27 (m, 3H), 6.79–6.74 (m, 1H), 6.65–6.62 (mz, 1H), 3.51 (s, 2H), 3.19–3.04 (m, 2H), 2.35–2.10 (m, 4H). ^13^C-NMR (126 MHz, CDCl_3_) δ 181.9, 164.2, 162.23, 143.2, 142.5, 142.4, 129.4, 129.1, 128.8, 128.6, 126.38, 126.36, 125.5, 125.3, 125.2, 125.1, 125.03, 125.00, 124.97, 109.4, 109.2, 98.7, 98.5, 713, 53.4, 51.3, 36.7, 22.2. ^19^F NMR (377 MHz, CDCl_3_) δ −62.4 (s), −111.3 (s). IR ν_max_ (KBr, film, cm^−1^): 3235, 2964, 2842, 1717, 1619, 1458, 1326, 1125, 1067, 1019, 810. HRMS (ESI): calcd for C_19_H_17_ON_2_F_4_^+^ [M + H]^+^: 365.1272, found: 365.1266.

*6-Chloro-1′-(4-(trifluoromethyl)benzyl)spiro[indoline-3,2′-pyrrolidin]-2-one* (**2m**): Syrup, 19.7 mg (from 0.10 mmol), 52% yield. ^1^H-NMR (300 MHz, CDCl_3_) δ 8.75 (br, 1H), 7.51–7.47 (m, 2H), 7.33–7.27 (m, 3H), 7.09–7.06 (m, 1H), 6.91–6.90 (m, 1H), 3.51 (s, 2H), 3.17–3.07 (m, 2H), 2.33–2.13 (m, 4H). ^13^C-NMR (126 MHz, CDCl_3_) δ 181.5, 143.2, 142.2, 134.5, 129.5, 128.6, 125.09, 125.05, 125.0, 123.0, 110.6, 71.3, 53.5, 51.3, 36.7, 22.3. ^19^F NMR (377 MHz, CDCl_3_) δ −62.4 (s). IR ν_max_ (KBr, film, cm^−1^):3232, 2963, 2938, 1713, 1616, 1486, 1325, 1124, 1066, 812. HRMS (ESI): calcd for C_19_H_17_ON_2_ClF_3_^+^ [M + H]^+^: 381.0976, found: 381.0971.

*1′-Phenylspiro[indoline-3,2′-pyrrolidin]-2-one* (**2n**): White solid, 19.0 mg (from 0.10 mmol), 72% yield, m.p. 140–142 °C. ^1^H-NMR (400 MHz, CDCl_3_) δ 9.03 (s, 1H), 7.22–7.18 (td, *J* = 7.6, 0.8 Hz, 1H), 7.13–7.11 (d, *J* = 7.6 Hz, 1H), 7.05–7.01 (m, 2H), 7.00–6.96 (m, 1H), 6.89–6.86 (d, *J* = 8.0 Hz, 1H), 6.63–6.59 (t, *J* = 7.2 Hz, 1H), 6.28–6.26 (d, *J* = 8.0 Hz, 2H), 3.85–3.82 (m, 2H), 2.57–2.52 (m, 1H), 2.46–2.37 (m, 1H), 2.33–2.18 (m, 2H). ^13^C-NMR (126 MHz, CDCl_3_) δ 181.5, 145.3, 139.2, 132.1, 129.0, 128.6, 123.02, 122.99, 117.0, 112.7, 110.8, 69.7, 50.5, 41.8, 23.0. IR ν_max_ (KBr, film, cm^−1^): 3202, 3092, 3059, 2922, 2851, 1717, 1505, 1469, 746. HRMS (ESI): calcd for C_17_H_15_ON_2_^−^ [M − H]^−^: 263.1190, found: 263.1191.

*1′-Benzylspiro[indoline-3,2′-piperidin]-2-one* (**2o**): White solid, 9.8 mg (from 0.10 mmol), 34% yield, m.p. 167–169 °C. ^1^H-NMR (500 MHz, CDCl_3_) δ 7.50 (br, 1H), 7.48–7.46 (d, *J* = 7.5 Hz, 1H), 7.26–7.25 (m, 4H), 7.23–7.18 (m, 2H), 7.09–7.06 (t, *J* = 7.5 Hz, 1H), 6.83–6.82 (d, *J* = 7.5 Hz, 1H), 3.38–3.36 (d, *J* = 13.0 Hz, 1H), 3.20–3.19 (d, *J* = 13.0 Hz, 1H), 3.16–3.11 (m, 1H), 2.71–2.67 (m, 1H), 2.11–2.04 (m, 1H), 1.96–1.88 (m, 2H), 1.76–1.72 (m, 1H), 1.69–1.62 (m, 2H). ^13^C-NMR (101 MHz, CDCl_3_) δ 180.7, 140.2, 139.4, 133.1, 128.5, 128.4, 128.0, 126.8, 124.1, 122.7, 109.7, 66.3, 56.3, 46.1, 35.3, 25.6, 19.1. IR ν_max_ (KBr, film, cm^−1^): 3210, 3061, 3028, 2929, 2851, 1702, 1619, 1472, 754. HRMS (ESI): calcd for C_19_H_19_ON_2_^−^ [M−H]^−^: 291.1503, found: 291.1504.

*1-Benzylspiro[azepane-2,3′-indolin]-2′-one* (**2p**): White solid, 4.7 mg (from 0.10 mmol), 15% yield, m.p. 177–179 °C. ^1^H-NMR (500 MHz, CDCl_3_) δ 7.67 (s, 1H), 7.62–7.61 (d, *J* = 7.5 Hz, 1H), 7.32–7.30 (m, 2H), 7.27 (s, 1H), 7.25 (s, 1H), 7.24–7.17 (m, 2H), 7.08–7.05 (t, *J* = 7.5 Hz, 1H), 6.85–6.83 (d, *J* = 7.5 Hz, 1H), 3.55–3.50 (dd, *J* = 15.0, 10.5 Hz, 1H), 3.44–3.42 (d, *J* = 13.5 Hz, 1H), 3.24–3.21 (d, *J* = 13.0 Hz, 1H), 2.68–2.64 (dd, *J* = 15.0, 6.0 Hz, 1H), 2.19–2.05 (m, 2H), 1.92–1.84 (m, 3H), 1.60 (m, 1H) 1.47–1.40 (m, 2H). ^13^C-NMR (126 MHz, CDCl_3_) δ 182.4, 140.3, 139.8, 134.8, 128.5, 128.2, 128.0, 126.8, 124.0, 122.7, 109.7, 69.6, 56.5, 47.2, 38.3, 32.4, 30.1, 22.7. IR ν_max_ (KBr, film, cm^−1^): 3207, 3028, 2925, 2853, 1704, 1651, 1469, 747. HRMS (ESI): calcd for C_20_H_23_ON_2_^+^ [M + H]^+^: 307.1805, found: 307.1808.

### 3.4. Further Functionalization

1′-Benzylspiro[indoline-3,2′-pyrrolidin]-2-one (**2a**, 0.5 mmol, 139.0 mg) was dissolved in dry THF (10 mL), B_2_H_6_ (2.5 mmol, 2.5 mL, 1 M in THF) was added slowly under the Ar. The mixture was heated to reflux for 5 h. And then to the vigorously stirring mixture were added methanol (5 ml) at 0 °C. After being stirred at 0 °C. 10 min, the mixture was warmed to room temperature and reflux for another 30 min. After this time, the solvent was removed under vacuum and residue was purified by silica column chromatography (elute: dichloromethane /methanol 10/1, with 1% NH_4_OH) to afford the desired product 1′-benzylspiro[indoline-3,2′-pyrrolidine] (**3**) as an orange solid.

*1′-Benzylspiro[indoline-3,2′-pyrrolidine]* (**3**): Orange solid, 70.0 mg (from 0.50 mmol), 53% yield, m.p. 79–81 °C. ^1^H-NMR (500 MHz, CDCl_3_) δ 8.14 (s, 1H), 7.59–7.58 (d, *J* = 7.5 Hz, 1H), 7.31–7.30 (m, 4H), 7.25–7.23 (m, 1H), 7.18–7.15 (t, *J* = 7.5 Hz, 1H), 7.11–7.09 (t, *J* = 7.5 Hz, 1H), 6.89 (s, 1H), 3.77 (s, 2H), 2.81–2.78 (t, *J* = 7.5 Hz, 2H), 2.73 (m, 2H), 2.06 (s, 1H), 1.95–1.92 (t, *J* = 7.5 Hz, 2H). ^13^C-NMR (126 MHz, CDCl_3_) δ 140.2, 136.3, 128.4, 128.2, 127.4, 126.9, 121.8, 121.2, 119.0, 118.8, 116.1, 111.0, 62.5, 53.9, 49.1, 30.2, 30.0, 22.8. IR ν_max_ (KBr, film, cm^−1^): 3414, 3241, 3057, 2926, 2849, 1456, 1098, 741, 697. HRMS (ESI): calcd for C_18_H_21_N_2_^+^ [M + H]^+^: 265.1699, found: 265.1695.

### 3.5. Control Experiment 

3-(3-(Benzylamino)propyl)indolin-2-one **(1a**, 0.10 mmol, 28.0 mg), TBAI (0.02 mmol, 7.4 mg) and additive (0.1 mmol) was dissolved in toluene (0.5 mL). 35% of H_2_O_2_ (0.6 mmol, 52.0 μL) was added and the reaction mixture was stirred at room temperature for 0.5 h. After that time, the mixture was quenched by saturated sodium thiosulfate solution (1 mL) and diluted with dichloromethane (10 mL). The organic layer was washed by water (10 mL × 2), dried over anhydrous sodium sulfate and evaporated to afford the crude product. The crude was purified by silica column chromatography (elute: petroleum ether/ethyl acetate 2/1) to give the pure product **2a**.

## 4. Conclusions

In summary, we have disclosed a new strategy for the construction of spirooxindoles via an intramolecular cyclization through an oxidative C-H/N-H bond coupling process under the catalysis of an iodide/H_2_O_2_ system. The representative synthetic examples demonstrate the inherent potential of this metal-free catalytic approach for the preparation of various 3,2′-pyrrolidinyl-spirooxindoles and their 6-/7-membered analogs. Further application of this method for the efficient synthesis of complex chiral 3,2′-pyrrolidinyl-spirooxindole products and other larger fused oxindoles are underway in our laboratory and will be reported in due course.

## Figures and Tables

**Table 1:**
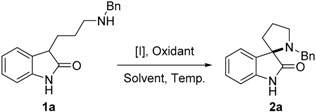
Studies and optimization of the reaction paramaters^1^.

Entry	[I]	Oxidant	Solvent	Temp. (°C)	Yield (%) ^2^
1 ^2,3^	TBAI	TBHP	H_2_O	60	41 ^4^
2 ^2,3^	I_2_	TBHP	H_2_O	60	33 ^5^
3	NaI	TBHP	H_2_O	RT	56 ^4^
4	NaI	H_2_O_2_	CH_3_CN	RT	58 ^4^
5	TBAI	H_2_O_2_	CH_3_CN	RT	62 ^4^
6	TBAI	H_2_O_2_	H_2_O	RT	33 ^4^
7	TBAI	H_2_O_2_	MeOH	RT	56 ^6^
8	TBAI	H_2_O_2_	THF	RT	64 ^6^
9 ^2,7^	TBAI	H_2_O_2_	Toluene	RT	79

^1^ Unless noted otherwise, all the reactions were conducted with 3-(3-(benzylamino)-propyl)-2-oxindole (**1a**, 0.1 mmol), catalyst (0.01 mmol), oxidant (6.0 equiv.) in the indicated solvent (1 mL) for 30 min. Isolated yields are given. ^2^ Catalyst loading: 20 mol·%. ^3^ Oxidant amount: 2.0 equiv. ^4^ Reaction time: 5 h. ^5^ Reaction time: 4 h. ^6^ Reaction time: 3.5 h. ^7^ Solvent volume: 0.5 mL.

## Supplementary Materials

The following supplementary information are available online: Experimental details, ^1^H and ^13^C NMR spectra of starting materials and products, X-Ray structural data for product **2k**.
