# Supplementary material for: Iodide/H2O2 Catalyzed Intramolecular Oxidative Amination for the Synthesis of 3,2′-Pyrrolidinyl Spirooxindoles
Source: Molecules. 2018 Sep 5;23(9):2265. doi: 10.3390/molecules23092265 (PMC6225319; doi:10.3390/molecules23092265)
Supplement: Supplementary file 1 [file molecules-23-02265-s001.pdf]

SUPPORTING INFORMATION

Iodide/H_2O_2 Catalyzed Intramolecular Oxidative Amination for the Synthesis of 3,2'-Pyrrolidinyl Spirooxindoles

Yu-Ting Gao,[a,b] Xiao-Yang Jin,[a,b] Qi Liu,[a,b] An-Di Liu,[a,b] Liang Cheng,*[a,b] Dong Wang,[a] and Li Liu*[a,b]

Table of Contents

1	General information	S2
2	General procedure for synthesis of 1	S3
3	General procedure for synthesis of 2	S8
4	Reduction of 2a	S13
5	Control experiment	S14
6	X-ray crystallographic data	S14
7	NMR spectra	S37
References		S74

General

Unless otherwise noted, all reagents were obtained from commercially suppliers and were used without further purification. All reactions were carried out under argon atmosphere using Schlenk techniques. Oxindoles **1** were obtained from commercially suppliers or prepared according to the literature procedures.[1-3] Alkynes were obtained from commercially suppliers.

TLC analysis was performed on glass-baked silica plates and visualized with UV light. Column chromatography was performed on silica gel (200-300 mesh) using petroleum ether / ethyl acetate / dichloromethane/methanol. ^1H, ^{13}C NMR Spectra were obtained on Bruker 300 MHz, 400 MHz or 500 MHz NMR spectrometer in the deuterated solvents indicated. Chemical shifts are reported in ppm from tetramethylsilane with the solvent resonance as the internal standard. The following abbreviations were used to designate chemical shift multiplicities: s = singlet, d = doublet, t = triplet, q = quartet, h = heptet, m = multiplet. All first-order splitting patterns were assigned on the basis of the appearance of the multiplet. Splitting patterns that could not be easily interpreted are designated as multiplet (m) or (br). Melting points were measured on Beijing Tech X-4 apparats without correction. IR spectra were recorded on a Nicolet 6700 FT-IR spectrometer. HRMS were obtained using electrospray ionization (ESI) mass spectrometer.

2. General procedure for synthesis of 1

To a mixture of indolyl propionic acid[S1] (10.0 mmol, 1.9 g) and triethylamine (20.0 mmol, 2.8 mL) in dichloromethane (70 mL) was added 1-[bis(dimethylamino)methylene]-1*H*-1,2,3-triazolo[4,5-*b*]pyridinium 3-oxid hexafluorophosphate (12.0 mmol, 4.6 g) and benzylamine (12.0 mmol, 1.3 mL). The mixture was stirred at room temperature for 1 hour and then diluted with dichloromethane (200 mL). The organic layer was washed by water (200 mL * 2), dried over anhydrous sodium sulfate and evaporated to afford the intermediate *N*-benzyl-3-(1*H*-indol-3-yl)propanamide without further purification. *N*-benzyl-3-(1*H*-indol-3-yl)propanamide (8.0 mmol, 2.3 g) was dissolved in dry tetrahydrofuran (40 mL) under argon, and then a solution of lithium aluminum hydride (32.0 mmol, 12.8 mL, 2.5 M in THF) was added dropwise. The mixture was heated to reflux overnight and then cooled to room temperature. To the vigorously stirring mixture were added H_2O (4 mL), 15% NaOH (4 mL), H_2O (4 mL * 3) at 0 °C. After being stirred at 0 °C for another 10 minutes, the mixture was filtered through celite, the white filter cake was washed with methanol and the filtrate was concentrated *in vacuum*. The crude was purified by silica column chromatography (elute: dichloromethane /methanol 10/1, with 1‰ NH_4OH) to afford the intermediate *N*-benzyl-3-(1*H*-indol-3-yl)propan-1-amine[S2] as a yellow oil. To the solution of *N*-benzyl-3-(1*H*-indol-3-yl)propan-1-amine (6.9 mmol,1.8 g) in dimethyl sulfoxide (20.7 mmol, 1.5 mL) and methanol (0.3 mL) was added concentrated hydrochloric acid (20.7 mmol, 1.7 mL) slowly at 0 °C. The resulting mixture was stirred at 50 °C for 5 h. After cooling to room temperature, the mixture was diluted with ethyl acetate (50 mL) and washed with H_2O (50 mL). Aqueous phase was adjust to pH = 7 by ammonium hydroxide and extracted with ethyl acetate (50 mL * 2). The organic was dried over anhydrous sodium sulfate, evaporated and purified by silica column chromatography (elute: dichloromethane /methanol 10/1, with 1‰ NH_4OH) to afford the desired product 3-(3-(benzylamino)propyl)indolin-2-one **1a**[S3].

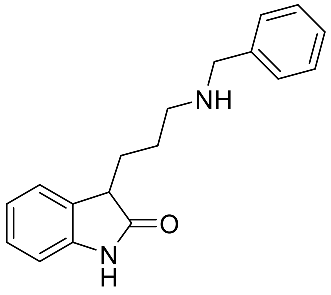

1a

3-(3-(benzylamino)propyl)indolin-2-one 1a. Yellow oil. ^{1}H NMR (300 MHz, $CDCl_3$) δ 8.47 (br, 1H), 7.31 (s, 1H), 7.30 (s, 2H), 7.24-7.16 (m, 3H), 7.03-6.98 (t, *J* = 7.5, 1H), 6.86-6.84 (d, *J* = 7.5 Hz, 1H), 3.78 (s, 2H), 3.49-3.45 (t, *J* = 6.0 Hz, 1H), 2.68-2.63 (m, 2H), 2.49 (br, 1H), 2.05-1.98 (dd, *J* = 14.1, 8.1 Hz, 2H), 1.66-1.53 (m, 2H). ^{13}C NMR (126 MHz, DMSO-d_6) δ 178.7, 142.7, 136.2, 129.4, 129.0, 128.3, 127.7, 127.6, 124.0, 121.2, 109.2, 51.3, 47.3, 44.7, 27.2, 23.6. IR ν_{max} (KBr, film, cm^{-1}): 3203, 3061, 2929, 2856, 1683, 1471, 751. HRMS (ESI): calcd for $C_{18}H_{21}ON_2^+$ $[M+H]^+$: 281.1648, found: 281.1647.

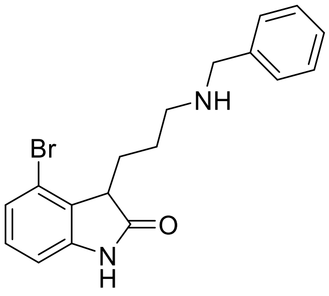

1b

3-(3-(benzylamino)propyl)-4-bromoindolin-2-one 1b. Pink solid, m. p. 83-85 °C. ^{1}H NMR (300 MHz, DMSO-d_6) δ 10.70 (s, 1H), 8.56 (bs, 1H), 7.43-7.38 (m, 6H), 7.16-7.14 (d, J = 5.4 Hz, 2H), 6.86-6.84 (d, J = 3.9 Hz, 1H), 4.01 (s, 2H), 3.60 (s, 1H), 2.84-2.79 (t, J = 7.8 Hz, 2H), 2.21-2.18 (m, 1H), 2.02-1.98 (m, 1H), 1.42-1.37 (m, 2H). ^{13}C NMR (126 MHz, DMSO-d_6) δ 177.3, 144.8, 133.0, 130.0, 129.8, 128.54, 128.49, 127.8, 124.6, 118.4, 108.7, 50.2, 46.5, 46.2, 24.6, 21.4. IR ν_{max} (KBr, film, cm^{-1}): 3360, 2920, 2848, 1698, 1458, 1019, 699. HRMS (ESI): calcd for $C_{18}H_{20}ON_2Br^+$ [M+H]$^+$: 359.0754, found: 359.0750.

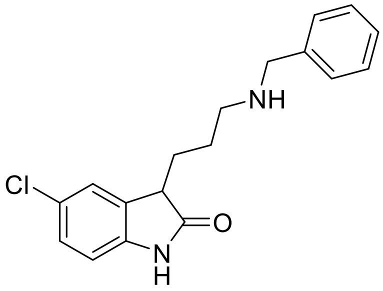

1c

3-(3-(benzylamino)propyl)-5-chloroindolin-2-one 1c. Orange solid, m. p. 89-91 °C. ^{1}H NMR (500 MHz, DMSO-d_6) δ 10.59 (s, 1H), 8.81 (br, 1H), 7.51-7.48 (m, 2H), 7.39-7.36 (m, 4H), 7.24-7.21 (t, J = 8.0 Hz, 1H), 6.85-6.82 (m,1H), 4.02 (s, 2H), 3.53-3.51 (t, J = 5.5 Hz, 1H), 2.83-2.80 (m, 2H), 1.89-1.85 (m, 2H), 1.60-1.58 (m, 2H). ^{13}C NMR (126 MHz, DMSO-d_6) δ 178.2, 141.7, 133.0, 131.5, 129.8, 128.6, 128.5, 127.5, 125.4, 124.3, 110.6, 50.3, 46.5, 44.8, 26.6, 22.2. IR ν_{max} (KBr, film, cm^{-1}): 3446, 2920, 2849, 1702, 1478, 699. HRMS (ESI): calcd for $C_{18}H_{20}ON_2Cl^+$ [M+H]$^+$: 315.1259, found: 315.1257.

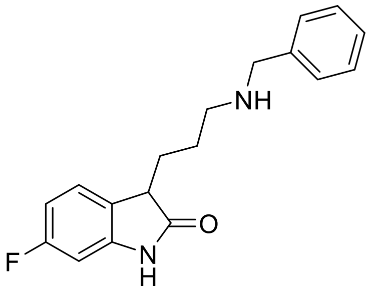

1d

3-(3-(benzylamino)propyl)-6-fluoroindolin-2-one 1d. Pink solid, m. p. 81-83 °C. ^{1}H NMR (500 MHz, DMSO-*d6*) δ 10.62 (s, 1H), 9.05 (br, 1H), 7.52-7.51 (m, 2H), 7.40 (m, 1H), 7.39-7.38 (m, 2H), 7.30-7.26 (m, 1H), 6.77-6.74 (m, 1H), 6.66-6.64 (m, 1H), 4.04 (s, 2H), 3.47-3.45 (t, J = 5.5 Hz, 1H), 2.85-2.82 (t, J = 6.5 Hz, 2H), 1.91-1.82 (m, 2H), 1.64-1.63 (m, 2H). ^{13}C NMR (126 MHz, DMSO-d_6) δ 178.9, 163.0, 161.0, 144.3, 144.2, 132.5, 129.9, 128.7, 128.5, 125.33, 125.26, 125.02, 125.00, 107.3, 107.1, 97.5, 97.3, 50.1, 46.4, 44.0, 26.9, 22.0. ^{19}F NMR (377 MHz, DMSO-*d6*) -

113.9(s). IR ν_{max} (KBr, film, cm^{-1}): 3359, 3195, 2920, 2849, 1702, 1469, 1340. HRMS (ESI): calcd for $C_{18}H_{20}ON_2F^+$ $[M+H]^+$: 299.1554, found: 299.1554.

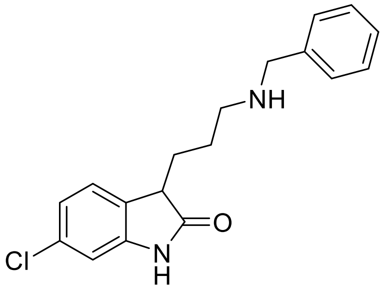

1e

3-(3-(benzylamino)propyl)-6-chloroindolin-2-one 1e. Orange solid, m. p. 89-91 °C. ^{1}H NMR (300 MHz, DMSO-d_6) δ 10.64 (s, 1H), 9.07 (br, 1H), 7.53-7.50 (m, 2H), 7.43-7.38(m, 3H), 7.30-7.27 (m, 1H), 7.01-6.87 (dd, *J* = 7.8, 1.5 Hz, 1H), 6.87-6.86 (d, *J* = 1.5 Hz, 1H), 4.04 (s, 2H), 3.50-3.47 (t, *J* = 5.7 Hz, 1H), 2.85-2.80 (t, *J* = 7.8 Hz, 2H), 1.91-1.81 (m, 2H), 1.65-1.63 (m, 2H). ^{13}C NMR (75 MHz, DMSO) δ 178.4, 144.3, 132.5, 131.9, 129.8, 128.6, 128.5, 128.1, 125.5, 120.8, 109.3, 50.0, 46.3, 44.1, 26.7, 21.9. IR ν_{max} (KBr, film, cm^{-1}): 3360, 3188, 2920, 2848, 1703, 1486, 749. HRMS (ESI): calcd for $C_{18}H_{20}ON_2Cl^+$ $[M+H]^+$: 315.1259, found: 315.1258.

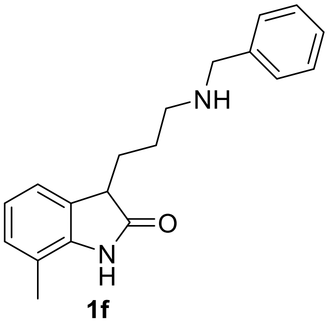

1f

3-(3-(benzylamino)propyl)-7-methylindolin-2-one 1f. White solid, m. p. 202-204 °C. ^{1}H NMR (300 MHz, DMSO-d_6) δ 10.45 (s, 1H), 9.00 (br, 1H), 7.51-7.48 (m, 2H), 7.45-7.40(m, 3H), 7.10-7.07 (d, *J* = 7.2 Hz, 1H), 7.01-6.98 (d, *J* = 7.8 Hz, 1H), 6.90-6.85 (t, *J* = 7.5 Hz, 1H), 4.07 (s, 2H), 3.49-3.48 (t, *J* = 5.4 Hz, 1H), 2.90-2.85 (m, 2H), 2.19 (s, 3H), 1.91-1.82 (m, 2H), 1.68-1.61 (m, 2H). ^{13}C NMR (126 MHz, DMSO-d_6) δ 179.0, 141.3, 132.2, 129.9, 129.0, 128.81, 128.78, 128.6, 121.3, 118.5, 50.1, 46.5, 44.8, 26.8, 22.0, 16.5. IR ν_{max} (KBr, film, cm^{-1}): 3392, 2946, 2838, 1702, 1458, 694. HRMS (ESI): calcd for $C_{19}H_{23}ON_2^+$ $[M+H]^+$: 295.1805, found: 295.1804.

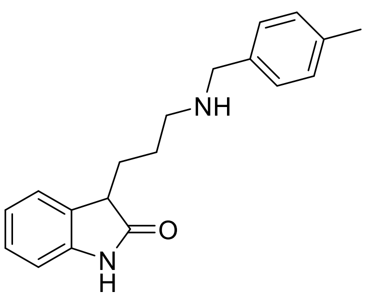

1g

3-(3-((4-methylbenzyl)amino)propyl)indolin-2-one 1g. Yellow oil. ^{1}H NMR (300 MHz, $CDCl_3$) δ 8.04 (br, 1H), 7.22-7.10 (m, 6H), 7.03-6.98 (t, *J* = 7.2 Hz, 1H), 6.85-6.83 (m, 1H), 3.71 (s, 2H), 3.49-3.46 (t, *J* = 6.0 Hz, 1H), 2.65-2.60 (t, *J* = 7.2 Hz, 2H), 2.32 (s, 3H), 2.02-1.97 (m, 2H), 1.72 (br, 1H), 1.64-1.52 (m, 2H). ^{13}C NMR (126 MHz, $CDCl_3$) δ 180.2, 141.6, 136.9, 136.5, 129.5, 129.1, 128.1, 127.8, 124.1, 122.2, 109.6, 53.5, 48.9, 45.7, 28.1, 26.0, 21.1. IR ν_{max} (KBr, film, cm^{-1}): 3204, 3022, 2923, 2857, 1706, 1620, 1486, 751. HRMS (ESI): calcd for $C_{19}H_{23}ON_2^+$ $[M+H]^+$: 295.1805, found: 295.1804.

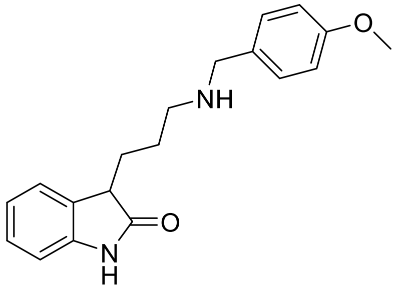

1h

3-(3-((4-methoxybenzyl)amino)propyl)indolin-2-one 1h. Yellow oil. ^{1}H NMR (300 MHz, DMSO-d_6) δ 10.34 (br, 1H), 7.23-7.13 (m, 4H), 6.96-6.91 (t, *J* = 7.5 Hz, 1H), 6.85-6.81 (m, 3H), 3.71 (s, 3H), 3.56 (s, 2H), 3.42-3.38 (t, *J* = 5.7 Hz, 1H), 2.97 (br, 1H), 2.46-2.41(t, *J* = 7.2 Hz, 2H), 1.89-1.79 (m, 2H), 1.43-1.37 (m, 2H). ^{13}C NMR (126 MHz, DMSO-d_6) δ 178.9, 158.0, 142.8, 132.6, 129.7, 129.1, 127.5, 123.9, 121.2, 113.4, 109.1, 54.9, 52.2, 48.3, 45.0, 27.7, 25.5. IR ν_{max} (KBr, film, cm^{-1}): 3197, 2933, 2835, 1698, 1471, 1177, 751. HRMS (ESI): calcd for $C_{19}H_{23}O_2N_2^+$ $[M+H]^+$: 311.1765, found: 311.1747.

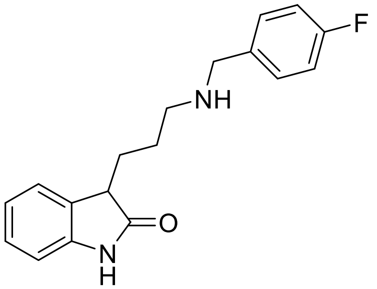

1i

3-(3-((4-fluorobenzyl)amino)propyl)indolin-2-one 1i. Yellow solid, m. p. 101-103 °C. ^{1}H NMR (300 MHz, DMSO-d_6) δ 10.42 (s, 1H), 8.86 (br, 1H), 7.57-7.52 (m, 2H), 7.28-7.15 (m, 4H), 6.98-6.93 (t, *J* = 7.5 Hz, 1H), 6.84-6.82 (d, *J* = 7.8 Hz, 1H), 4.05 (s, 2H), 3.50-3.48 (t, *J* = 5.7 Hz, 1H), 2.87-2.82 (t, *J* = 7.8 Hz, 2H), 1.89-1.82 (m, 2H), 1.67-1.59 (m, 2H). ^{13}C NMR (101 MHz, DMSO-d_6) δ 178.5, 163.4, 161.0, 142.7, 132.3, 132.3, 129.2, 128.6, 127.6, 124.0, 121.2, 115.4, 115.2, 109.2, 49.1, 46.2, 44.5, 26.9, 22.0. ^{19}F NMR (377 MHz, DMSO-d_6) δ -113.9(s). IR ν_{max} (KBr, film, cm^{-1}): 3361, 2920, 2849, 1703, 1471, 1226, 751. HRMS (ESI): calcd for $C_{18}H_{20}ON_2F^+$ $[M+H]^+$: 299.1554, found: 299.1553.

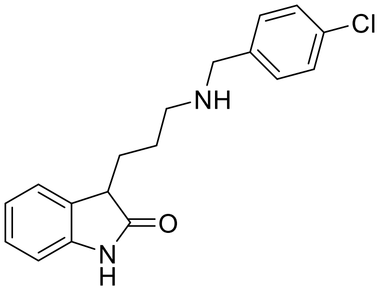

1j

3-(3-((4-chlorobenzyl)amino)propyl)indolin-2-one 1j. Yellow oil. ^{1}H NMR (300 MHz, $CDCl_3$) δ 8.76 (br, 1H), 7.31-7.28 (m, 1H), 7.25-7.17(m, 5H), 7.03-6.98 (t, *J* = 7.5 Hz, 1H), 6.87-6.84 (d, *J* = 7.8 Hz, 1H), 3.72 (s, 2H), 3.49-3.45 (t, *J* = 5.7 Hz, 1H), 2.64-2.59 (t, *J* = 7.2 Hz, 2H), 2.24-2.19 (m, 1H), 2.04-1.97 (dd, *J* = 14.1, 7.8 Hz, 2H), 1.62-1.51 (m, 2H). ^{13}C NMR (126 MHz, $CDCl_3$) δ 180.2, 141.6, 138.3, 132.7, 129.5, 129.4, 128.5, 127.9, 124.1, 122.3, 109.7, 53.0, 48.7, 45.7, 28.0, 25.9. IR ν_{max} (KBr, film, cm^{-1}): 3200, 2932, 1714, 1471, 1015, 751. HRMS (ESI): calcd for $C_{18}H_{20}ON_2Cl^+$ $[M+H]^+$: 315.1259, found: 315.1256.

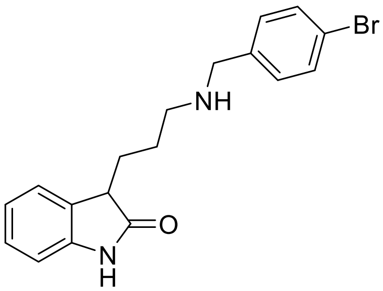

1k

3-(3-((4-bromobenzyl)amino)propyl)indolin-2-one 1k. Yellow solid, m. p. 102-104 °C. ^{1}H NMR (300 MHz, DMSO-d_6) δ 10.41 (s, 1H), 8.33 (br, 1H), 7.60-7.58 (d, *J* = 8.1 Hz, 2H), 7.44-7.41 (d, *J* = 8.1 Hz, 2H), 7.27-7.24 (d, *J* = 7.2 Hz, 1H), 7.20-7.15 (t, *J* = 7.8 Hz, 1H), 6.97-6.92 (t, *J* = 7.5 Hz, 1H), 6.84-6.81 (d, *J* = 7.8 Hz, 1H), 3.96 (s, 2H), 3.48-3.44 (t, *J* = 5.7 Hz, 1H), 2.80-2.74 (t, *J* = 7.5 Hz, 2H), 1.91-1.80 (m, 2H), 1.64-1.55 (m, 2H). ^{13}C NMR (75 MHz, DMSO-d_6) δ 178.6, 142.7, 131.8, 131.3, 129.3, 127.7, 124.0, 121.6, 121.2, 109.2, 49.8, 46.8, 44.7, 26.9, 22.7. IR ν_{max} (KBr, film, cm^{-1}): 3366, 3197, 2922, 2850, 1702, 1622, 1471, 753. HRMS (ESI): calcd for $C_{18}H_{20}ON_2Br^+$ $[M+H]^+$: 359.0754, found: 359.0740.

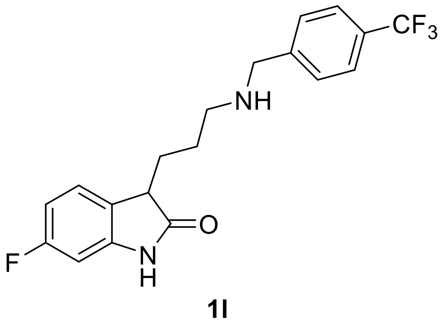

1l

6-fluoro-3-(3-((4-(trifluoromethyl)benzyl)amino)propyl)indolin-2-one 1l. Orange oil. ^{1}H NMR (300 MHz, DMSO-d_6) δ 10.56 (br, 1H), 7.69-7.66 (d, *J* = 7.8 Hz, 2H), 7.58-7.55 (d, *J* = 7.8 Hz, 2H), 7.30-7.25 (m, 1H), 6.81-6.67 (m, 2H), 3.77 (s, 2H), 3.48-3.44 (t, *J* = 6.3Hz, 1H), 2.56 (s, 1H), 1.95-1.87 (m, 3H), 1.49-1.41 (m, 3H). ^{13}C NMR (126 MHz, DMSO-d_6) δ 179.3, 162.9, 161.0, 146.1, 144.3, 144.2, 129.5, 125.5, 125.13, 125.05, 124.8, 107.2, 107.0, 97.4, 97.2,

52.4, 48.5, 44.5, 27.7, 25.5. ^{19}F NMR (377 MHz, DMSO-d_6) δ -60.86(s), -113.93 (s). IR ν_{max} (KBr, film, cm^{-1}): 3633, 2952, 2855, 1717, 1558, 1329, 1020, 849, 737. HRMS (ESI): calcd for $C_{19}H_{19}ON_2F_4^+$ [M+H]$^+$: 367.1428, found: 367.1422.

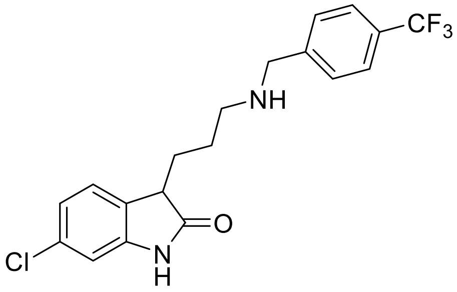

1m

6-chloro-3-(3-((4-(trifluoromethyl)benzyl)amino)propyl)indolin-2-one 1m. Orange oil. ^{1}H NMR (300 MHz, DMSO-d_6) δ 10.57 (br, 1H), 7.71-7.68 (d, *J* = 7.8 Hz, 2H), 7.58-7.56 (d, *J* = 7.8 Hz, 2H), 7.30-7.28 (m, 1H), 7.05-7.02 (m, 1H), 6.89 (s, 1H), 3.77 (s, 2H), 3.52-3.48 (t, *J* = 5.7 Hz, 1H), 2.57 (s, 1H), 1.97-1.86 (m, 3H), 1.50-1.38 (m, 3H). ^{13}C NMR (126 MHz, DMSO-d_6) δ 178.9, 146.1, 144.3, 131.8, 128.6, 128.4, 125.3, 124.83, 124.80, 120.8, 109.2, 52.3, 48.5, 44.6, 27.5, 25.5. ^{19}F NMR (377 MHz, DMSO-d_6) δ -60.79 (s). IR ν_{max} (KBr, film, cm^{-1}): 3419, 3181, 2952, 2800, 1704, 1619, 1326, 1127, 1068, 737. HRMS (ESI): calcd for $C_{19}H_{19}ON_2ClF_3^+$ [M+H]$^+$: 382.1133, found: 383.1126.

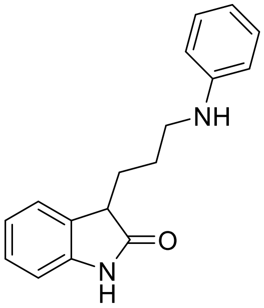

1n

3-(3-(phenylamino)propyl)indolin-2-one 1n. Pale yellow solid, m. p. 105-107 °C. ^{1}H NMR (500 MHz, CDCl$_3$) δ 8.87 (s, 1H), 7.24-7.22 (m, 2H), 7.17-7.14 (t, *J* = 7.5 Hz, 2H), 7.06-7.03 (t, *J* = 7.5 Hz, 1H), 6.93-6.91 (d, *J* = 8.5 Hz, 1H), 6.70-6.67 (m, 1H), 6.57-6.56 (d, *J* = 8.5 Hz, 2H), 3.67 (br, 1H), 3.56-3.54 (t, *J* = 5.5 Hz, 2H), 3.14-3.11 (t, *J* = 7.0 Hz, 2H), 2.13-2.09 (m, 2H), 1.77-1.66 (m, 2H). ^{13}C NMR (126 MHz, CDCl$_3$) δ 180.4, 148.2, 141.6, 129.3, 129.2, 128.0, 124.0, 122.4, 117.2, 112.7, 109.8, 45.7, 43.7, 27.9, 25.7. IR ν_{max} (KBr, film, cm^{-1}): 3368, 3210, 2925, 2855, 1707, 1602, 1471, 749. HRMS (ESI): calcd for $C_{17}H_{19}ON_2^+$ [M+H]$^+$: 267.1492, found: 267.1494.

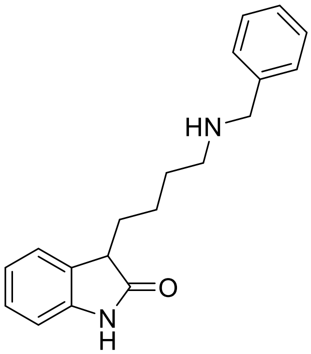

1o

3-(4-(benzylamino)butyl)indolin-2-one 1o. Pale yellow solid, m. p. 172-173 °C. ^{1}H NMR (300 MHz, DMSO-d_6) δ 10.41 (s, 1H), 8.66 (br, 1H), 7.52-7.50 (m, 2H), 7.38-7.36 (m, 3H), 7.26-7.24 (d, J = 7.5 Hz, 1H),7.19-7.14 (t, J = 7.5 Hz, 1H), 6.96-6.91 (t, J = 7.2 Hz, 1H), 6.84-6.82 (d, J = 7.5 Hz, 1H), 3.99 (s, 2H), 3.43-3.39 (m, 1H), 2.77- 2.71 (t, J = 7.5 Hz, 2H), 1.85-1.76 (m, 2H), 1.65-1.60 (m, 2H), 1.32-1.24 (m, 2H). ^{13}C NMR (101 MHz, DMSO-d_6) δ 178.7, 142.7, 133.3, 129.7, 129.5, 128.42, 128.38, 127.5, 123.9, 121.1, 109.1, 50.2, 46.4, 44.9, 29.5, 25.8, 22.6. IR ν_{max} (KBr, film, cm^{-1}): 3359, 2920, 2849, 1702, 1472, 751. HRMS (ESI): calcd for $C_{19}H_{23}ON_2^+$ [M+H]$^+$: 295.1805, found: 295.1802.

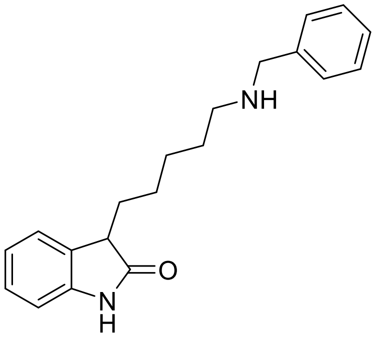

1p

3-(5-(benzylamino)pentyl)indolin-2-one 1p. Yellow oil. ^{1}H NMR (300 MHz, $CDCl_3$) δ 8.47 (br, 1H), 7.34-7.28 (m, 5H), 7.24-7.20 (m, 2H), 7.05-7.00 (m, 1H), 6.90-6.88 (d, J = 8.1 Hz, 1H), 3.80 (s, 2H), 3.49-3.45 (t, J = 6.0 Hz, 1H), 2.65-2.60 (t, J = 6.9 Hz, 2H), 2.22 (br, 1H), 2.00-1.95 (m, 2H), 1.55-1.48 (m, 2H), 1.45-1.36 (m, 4H). ^{13}C NMR (126 MHz, $CDCl_3$) δ 180.2, 141.5, 139.7, 129.7, 128.4, 128.2, 127.8, 127.0, 124.1, 122.2, 109.6, 53.8, 49.0, 45.9, 30.4, 29.5, 27.2, 25.5. IR ν_{max} (KBr, film, cm^{-1}): 3197, 3061, 2830, 2856, 1683, 1506, 1471, 749. HRMS (ESI): calcd for $C_{20}H_{25}ON_2^+$ [M+H]$^+$: 309.1961, found: 309.1959.

3. General procedure for synthesis of 2

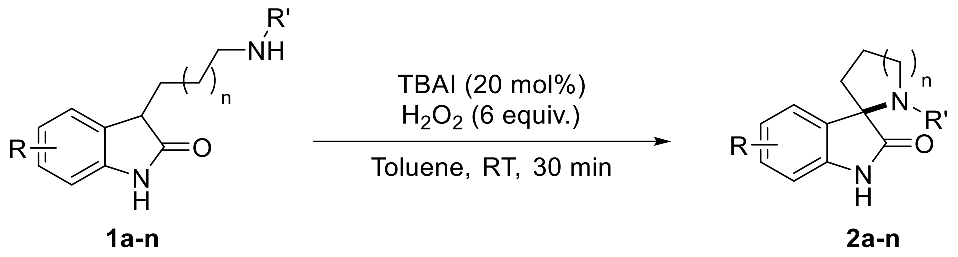

To the mixture of oxindole **1** (0.10 mmol) and TBAI (20 mol%) in toluene (0.5 mL) was added 35% H_2O_2 (6 equiv.), the reaction mixture was stirred at room temperature until completion the reaction. After that time, the mixture was quenched by saturated sodium thiosulfate solution (1 mL) and diluted with dichloromethane (10 mL). The organic layer was washed by water (10 mL * 2), dried over anhydrous sodium sulfate and evaporated to afford the crude product. The crude was purified by silica column chromatography (elute: petroleum ether/ethyl acetate 2/1) to give the pure desired products **2**.

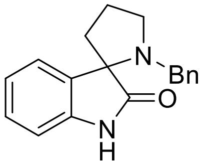

2a

1'-benzylspiro[indoline-3,2'-pyrrolidin]-2-one 2a. White solid, 21.9 mg (from 0.10 mmol), 79% yield, m. p. 154-156 °C. ^{1}H NMR (500 MHz, DMSO-d_6) δ 10.28 (s, 1H), 7.35-7.33 (d, J = 7.5 Hz, 1H), 7.26-7.22 (m, 2H), 7.20-7.18 (m, 4H), 7.03-7.00 (t, J = 7.5 Hz, 1H), 6.80-6.79 (d, J = 7.5 Hz, 1H), 3.31-3.25 (m, 2H), 2.98-2.91 (m, 2H), 2.14-2.06 (m, 2H), 2.04-1.98 (m, 2H). ^{13}C NMR (126 MHz, DMSO-d_6) δ 179.7, 142.4, 139.3, 130.7, 128.7, 128.1, 127.9, 126.8, 123.8, 121.9, 109.4, 70.8, 53.1, 50.4, 35.8, 21.7. IR ν_{max} (KBr, film, cm^{-1}): 3207, 3061, 3028, 2925, 2852, 1706, 1620, 1470, 749. HRMS (ESI): calcd for $C_{18}H_{17}ON_2^-$ [M-H]$^-$: 277.1364, found: 277.1364.

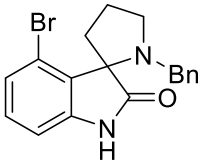

2b

1'-benzyl-4-bromospiro[indoline-3,2'-pyrrolidin]-2-one 2b. White solid, 17.6 mg (from 0.10 mmol), 49% yield, m. p. 169-171 °C. ^{1}H NMR (500 MHz, $CDCl_3$) δ 8.74 (s, 1H), 7.32-7.31 (d, J = 7.5 Hz, 2H), 7.26-7.15 (m, 4H), 7.08-7.05 (m, 1H), 6.81-6.80 (dd, J = 7.5, 1.0 Hz, 1H), 3.59-3.49 (m, 2H), 3.17-3.10 (m, 2H), 2.68-2.63 (m, 1H), 2.25- 2.18 (m, 3H). ^{13}C NMR (126 MHz, $CDCl_3$) δ 181.3, 143.2, 139.5, 130.1, 128.7, 128.5, 128.0, 127.4, 126.8, 119.9, 108.9, 72.5, 53.4, 51.1, 32.8, 23.1. IR ν_{max} (KBr, film, cm^{-1}): 3213, 3086, 3027, 2964, 2831, 1717, 1613, 1447, 736. HRMS (ESI): calcd for $C_{18}H_{18}ON_2Br^+$ [M+H]$^+$: 357.0597, found: 354.0594.

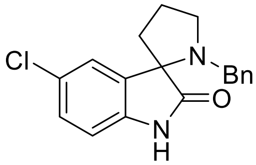

2c

1'-benzyl-5-chlorospiro[indoline-3,2'-pyrrolidin]-2-one 2c.White solid, 14.3 mg (from 0.10 mmol), 46% yield, m. p. 149-151 °C. ^{1}H NMR (500 MHz, $CDCl_3$) δ 8.47 (s, 1H), 7.34 (s, 1H), 7.25-7.19 (m, 6H), 6.79-6.78 (d, J = 8.0 Hz, 1H), 3.52-3.45 (m, 2H), 3.18-3.13 (m, 1H), 3.10-3.07 (m, 1H), 2.35-2.31 (m, 1H), 2.24-2.21 (m, 1H), 2.17-2.08 (m, 2H). ^{13}C NMR (126 MHz, $CDCl_3$) δ 181.2, 139.5, 138.9, 133.4, 128.6, 128.5, 128.2, 128.1, 127.0, 124.6, 110.8, 71.7, 53.9, 51.4, 37.0, 22.3. IR ν_{max} (KBr, film, cm^{-1}): 3213, 3063, 3029, 2963, 2840, 1717, 1619, 1475, 733. HRMS (ESI): calcd for $C_{18}H_{18}ON_2Br^+$ $[M+H]^+$: 313.1102, found: 313.1102.

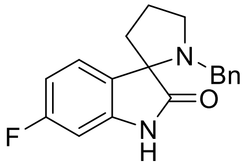

2d

1'-benzyl-6-fluorospiro[indoline-3,2'-pyrrolidin]-2-one 2d.White solid, 15.3 mg (from 0.10 mmol), 52% yield, m. p. 141-143 °C. ^{1}H NMR (300 MHz, $CDCl_3$) δ 8.88 (s, 1H), 7.33-7.29 (m, 1H), 7.23-7.15 (m, 5H), 6.81-6.74 (m, 1H), 6.65-6.62 (dd, J = 14.5, 2.1 Hz, 1H), 3.52-3.41 (m, 2H), 3.22-3.06 (m, 2H), 2.35-2.04 (m, 4H). ^{13}C NMR (126 MHz, $CDCl_3$) δ 182.1, 164.1, 162.2, 142.5, 142.4, 139.0, 128.5, 128.1, 126.9, 126.7, 126.6, 125.3, 125.2, 109.2, 109.0, 98.7, 98.4, 71.2, 53.8, 51.2, 36.7, 22.1. ^{19}F NMR (377 MHz, $CDCl_3$) δ -111.7 (s). IR ν_{max} (KBr, film, cm^{-1}): 3226, 3063, 3029, 2965, 2836, 1717, 1622, 1456, 733. HRMS (ESI): calcd for $C_{18}H_{18}ON_2F^+$ $[M+H]^+$: 297.1398, found: 297.1400.

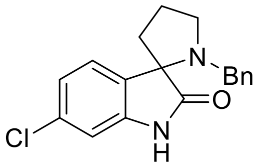

2e

1'-benzyl-6-chlorospiro[indoline-3,2'-pyrrolidin]-2-one 2e.White solid, 19.0 mg (from 0.10 mmol), 61% yield, m. p. 170-172 °C. ^{1}H NMR (500 MHz, $CDCl_3$) δ 8.67 (s, 1H), 7.30-7.28 (m, 1H), 7.25-7.21 (m, 2H), 7.20-7.18 (m, 3H), 7.08-7.05 (m, 1H), 6.89 (s, 1H), 3.50-3.42 (m, 2H), 3.19-3.14 (m, 1H), 3.10-3.06 (m, 1H), 2.35-2.29 (m, 1H), 2.27-2.22 (m, 1H), 2.17-2.07 (m, 2H). ^{13}C NMR (126 MHz, $CDCl_3$) δ 181.6, 142.2, 139.0, 134.3, 129.8, 128.5, 128.1, 127.0, 125.2, 122.8, 110.5, 71.3, 53.9, 51.2, 36.7, 22.2. IR ν_{max} (KBr, film, cm^{-1}): 3232, 3064, 3029, 2965, 2834, 1717, 1615, 1455, 732. HRMS (ESI): calcd for $C_{18}H_{18}ON_2Cl^+$ $[M+H]^+$: 313.1102, found: 313.1101.

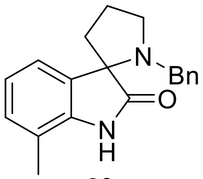

2f

1'-benzyl-7-methylspiro[indoline-3,2'-pyrrolidin]-2-one 2f.White solid, 20.5 mg (from 0.10 mmol), 70% yield, m. p. 148-150 °C. ^{1}H NMR (500 MHz, $CDCl_3$) δ 8.83 (s, 1H), 7.23-7.16 (m, 6H), 7.07-7.05 (m, 1H), 7.03-7.00 (m, 1H), 3.51-3.42 (m, 2H), 3.20-3.15 (m, 1H), 3.10-3.06 (m, 1H), 2.35-2.31 (m, 1H), 2.29 (s, 3H), 2.27-2.21 (m, 1H), 2.19-2.14 (m, 1H), 2.11-2.08 (m, 1H). ^{13}C NMR (126 MHz, $CDCl_3$) δ 182.0, 139.9, 139.4, 130.9, 130.0, 128.5, 128.0, 126.8, 122.7, 121.5, 119.0, 72.0, 54.0, 51.2, 36.7, 22.2, 16.2. IR ν_{max} (KBr, film, cm^{-1}): 3280, 3061, 3028, 2964, 2837, 1704, 1627, 1458, 732. HRMS (ESI): calcd for $C_{19}H_{21}ON_2^+$ $[M+H]^+$: 293.1648, found: 293.1648.

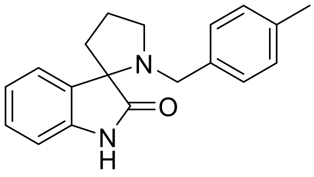

2g

1'-(4-methylbenzyl)spiro[indoline-3,2'-pyrrolidin]-2-one 2g.White solid, 22.7 mg (from 0.10 mmol), 79% yield, m. p. 96-98 °C. ^{1}H NMR (500 MHz, $CDCl_3$) δ 9.03 (s, 1H), 7.37-7.36 (d, *J* = 7.0 Hz, 1H), 7.23-7.20 (t, *J* = 8.0 Hz, 1H), 7.09-7.06 (m, 3H), 7.03-7.01 (m, 2H), 6.89-6.87 (d, *J* = 8.0 Hz, 1H), 3.45-3.38 (m, 2H), 3.20-3.15 (m, 1H), 3.09 -3.05 (m, 1H), 2.35-2.30 (m, 1H), 2.26 (s, 3H), 2.22-2.04 (m, 3H). ^{13}C NMR (126 MHz, $CDCl_3$) δ 182.0, 141.3, 136.3, 136.2, 131.4, 128.7, 128.6, 128.4, 124.1, 122.7, 109.9, 71.7, 53.5, 51.1, 36.6, 22.2, 21.0. IR ν_{max} (KBr, film, cm^{-1}): 3215, 3025, 2971, 2830, 1706, 1620, 1471, 750. HRMS (ESI): calcd for $C_{19}H_{21}ON_2^+$ $[M+H]^+$: 293.1648, found: 293.1647.

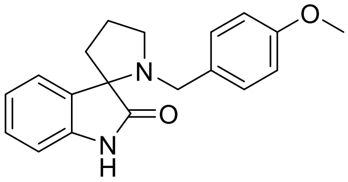

2h

1'-(4-methoxybenzyl)spiro[indoline-3,2'-pyrrolidin]-2-one 2h.White solid, 17.1 mg (from 0.10 mmol), 56% yield, m. p. 135-137 °C. ^{1}H NMR (500 MHz, $CDCl_3$) δ 8.52 (s, 1H), 7.37-7.36 (d, *J* = 7.0 Hz, 1H), 7.24-7.21 (t, *J* = 7.5 Hz, 1H), 7.10-7.07 (m, 3H), 6.87-6.85 (d, *J* = 8.0 Hz, 1H), 6.77-6.75 (d, *J* = 8.0 Hz, 2H), 3.73 (s, 3H), 3.44-3.36 (m, 2H), 3.20-3.15 (m, 1H), 3.09-3.05 (m, 1H), 2.35-2.30 (m, 1H), 2.24-2.21 (m, 1H), 2.18-2.07 (m, 2H). ^{13}C NMR (126 MHz, $CDCl_3$) δ 181.6, 158.5, 141.2, 131.5, 131.4, 129.7, 128.6, 124.2, 122.7, 113.4, 109.8, 71.5, 55.1, 53.3, 51.2, 36.7, 22.2. IR ν_{max} (KBr, film, cm^{-1}): 3251, 2962, 2834, 1700, 1622, 1471, 751. HRMS (ESI): calcd for $C_{19}H_{19}ON_2^-$ $[M-H]^-$: 307.1452, found: 307.1454.

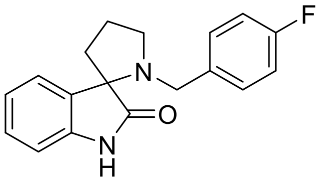

2i

1'-(4-fluorobenzyl)spiro[indoline-3,2'-pyrrolidin]-2-one 2i.White solid, 10.9 mg (from 0.10 mmol), 37% yield, m. p. 126-127 °C. ^{1}H NMR (500 MHz, $CDCl_3$) δ 7.89 (s, 1H), 7.36-7.35 (d, *J* = 7.0 Hz, 1H), 7.24-7.21 (td, *J* = 7.5, 1.0 Hz, 1H), 7.16-7.13 (m, 2H), 7.10-7.07 (t, *J* = 7.5 Hz, 1H), 6.92-6.88 (m, 2H), 6.84-6.82 (d, *J* = 7.5 Hz, 1H), 3.46-3.39 (m, 2H), 3.17-3.13 (m, 1H), 3.07-3.03 (m, 1H), 2.35-2.30 (m, 1H), 2.28-2.20 (m, 1H), 2.18-2.13 (m, 1H), 2.11-2.06 (m, 1H). ^{13}C NMR (126 MHz, $CDCl_3$) δ 181.6, 162.8, 160.9, 141.1, 134.9, 131.3, 130.1, 130.0, 128.7, 124.1, 122.8, 114.8, 114.7, 109.9, 71.5, 53.2, 51.3, 36.7, 22.2. ^{19}F NMR (377 MHz, $CDCl_3$) δ -116.1 (s). IR ν_{max} (KBr, film, cm^{-1}): 3213, 3086, 2964, 2836, 1717, 1622, 1471, 750. HRMS (ESI): calcd for $C_{18}H_{18}ON_2F^+$ $[M+H]^+$: 297.1398, found: 297.1395.

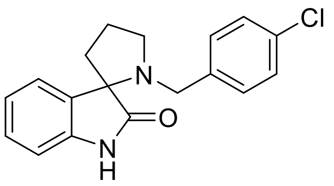

2j

1'-(4-chlorobenzyl)spiro[indoline-3,2'-pyrrolidin]-2-one 2j. White solid, 23.3 mg (from 0.10 mmol), 74% yield, m. p. 140-142 °C. ^{1}H NMR (500 MHz, $CDCl_3$) δ 8.54 (s, 1H), 7.36-7.34 (d, *J* = 7.5 Hz, 1H), 7.25-7.21 (m, 1H), 7.19-7.18 (m, 2H), 7.13-7.12 (m, 2H), 7.10-7.07 (td, *J* = 7.5, 0.5 Hz, 1H), 6.88-6.86 (d, *J* = 7.5 Hz, 1H), 3.46-3.39 (m, 2H), 3.17-3.13 (m, 1H), 3.07-3.04 (m, 1H), 2.36-2.31 (m, 1H), 2.28-2.21 (m, 1H), 2.19-2.13 (m, 1H), 2.12-2.04 (m, 1H). ^{13}C NMR (126 MHz, $CDCl_3$) δ 181.5, 141.1, 137.8, 132.5, 131.2, 129.8, 128.7, 128.1, 124.1, 122.7, 109.9, 71.5, 53.2, 51.3, 36.7, 22.2. IR ν_{max} (KBr, film, cm^{-1}): 3212, 2925, 2849, 1705, 1622, 1471, 750. HRMS (ESI): calcd for $C_{18}H_{18}ON_2Cl^+$ $[M+H]^+$: 313.1102, found: 313.1099.

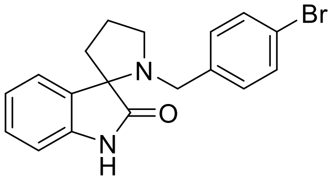

2k

1'-(4-bromobenzyl)spiro[indoline-3,2'-pyrrolidin]-2-one 2k. White solid, 19.6 mg (from 0.10 mmol), 55% yield, m. p. 140-142 °C. ^{1}H NMR (500 MHz, $CDCl_3$) δ 8.36 (s, 1H), 7.35-7.33 (m, 3H), 7.24-7.21 (t, *J* = 7.5 Hz, 1H), 7.09-7.07 (m, 3H), 6.86-6.85 (d, *J* = 8.0 Hz, 1H), 3.43-3.38 (m, 2H), 3.17-3.12 (q, *J* = 7.5 Hz, 1H), 3.07-3.03 (m, 1H), 2.35-2.30 (m, 1H), 2.28-2.20 (m, 1H), 2.19-2.13 (m, 1H), 2.12-2.08 (m, 1H). ^{13}C NMR (126 MHz, $CDCl_3$) δ 181.3, 141.1, 138.3, 131.2, 131.1, 130.2, 128.7, 124.2, 122.9, 120.6, 109.8, 71.5, 53.3, 51.3, 36.7, 22.3. IR ν_{max} (KBr, film, cm^{-1}): 3216, 3090, 2925, 2851, 1706, 1621, 1470, 750. HRMS (ESI): calcd for $C_{18}H_{18}ON_2Br^+$ $[M+H]^+$: 357.0597, found: 357.0594.

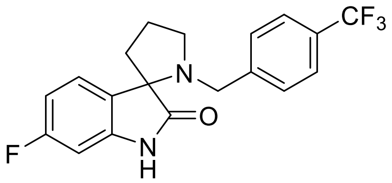

2l

6-fluoro-1'-(4-(trifluoromethyl)benzyl)spiro[indoline-3,2'-pyrrolidin]-2-one 2l. Syrup, 20.5 mg (from 0.10 mmol), 56% yield. ^{1}H NMR (300 MHz, $CDCl_3$) δ 8.84 (br, 1H), 7.50-7.47 (d, J = 7.8 Hz, 2H), 7.33-7.27 (m, 3H), 6.79-6.74 (m, 1H), 6.65-6.62 (mz, 1H), 3.51 (s, 2H), 3.19-3.04 (m, 2H), 2.35-2.10 (m, 4H). ^{13}C NMR (126 MHz, $CDCl_3$) δ 181.9, 164.2, 162.23, 143.2, 142.5, 142.4, 129.4, 129.1, 128.8, 128.6, 126.38, 126.36, 125.5, 125.3, 125.2, 125.1, 125.03, 125.00, 124.97, 109.4, 109.2, 98.7, 98.5, 713, 53.4, 51.3, 36.7, 22.2. ^{19}F NMR (377 MHz, $CDCl_3$) δ -62.4 (s), -111.3 (s). IR ν_{max} (KBr, film, cm^{-1}): 3235, 2964, 2842, 1717, 1619, 1458, 1326, 1125, 1067, 1019, 810. HRMS (ESI): calcd for $C_{19}H_{17}ON_2F_4^+$ $[M+H]^+$: 365.1272, found: 365.1266.

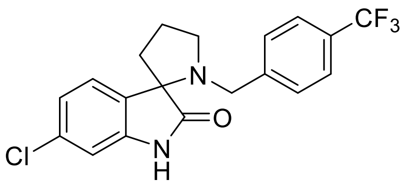

2m

6-chloro-1'-(4-(trifluoromethyl)benzyl)spiro[indoline-3,2'-pyrrolidin]-2-one 2m. Syrup, 19.7 mg (from 0.10 mmol), 52% yield. ^{1}H NMR (300 MHz, $CDCl_3$) δ 8.75 (br, 1H), 7.51-7.47 (m, 2H), 7.33-7.27 (m, 3H), 7.09-7.06 (m, 1H), 6.91-6.90 (m, 1H), 3.51 (s, 2H), 3.17-3.07 (m, 2H), 2.33-2.13 (m, 4H). ^{13}C NMR (126 MHz, $CDCl_3$) δ 181.5, 143.2, 142.2, 134.5, 129.5, 128.6, 125.09, 125.05, 125.0, 123.0, 110.6, 71.3, 53.5, 51.3, 36.7, 22.3. ^{19}F NMR (377 MHz, $CDCl_3$) δ -62.4 (s). IR ν_{max} (KBr, film, cm^{-1}):3232, 2963, 2938, 1713, 1616, 1486, 1325, 1124, 1066, 812. HRMS (ESI): calcd for $C_{19}H_{17}ON_2ClF_3^+$ $[M+H]^+$: 381.0976, found: 381.0971.

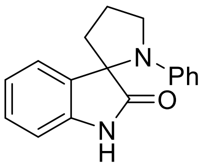

2n

1'-phenylspiro[indoline-3,2'-pyrrolidin]-2-one 2n. White solid, 19.0 mg (from 0.10 mmol), 72% yield, m. p. 140-142 °C. ^{1}H NMR (400 MHz, $CDCl_3$) δ 9.03 (s, 1H), 7.22-7.18 (td, *J* = 7.6, 0.8 Hz, 1H), 7.13-7.11 (d, *J* = 7.6 Hz, 1H), 7.05-7.01 (m, 2H), 7.00-6.96 (m, 1H), 6.89-6.86 (d, *J* = 8.0 Hz, 1H), 6.63-6.59 (t, *J* = 7.2 Hz, 1H), 6.28-6.26 (d, *J* = 8.0 Hz, 2H), 3.85-3.82 (m, 2H), 2.57-2.52 (m, 1H), 2.46-2.37 (m, 1H), 2.33-2.18 (m, 2H). ^{13}C NMR (126 MHz, $CDCl_3$) δ 181.5, 145.3, 139.2, 132.1, 129.0, 128.6, 123.02, 122.99, 117.0, 112.7, 110.8, 69.7, 50.5, 41.8, 23.0. IR ν_{max} (KBr, film, cm^{-1}): 3202, 3092, 3059, 2922, 2851, 1717, 1505, 1469, 746. HRMS (ESI): calcd for $C_{17}H_{15}ON_2^-$ $[M-H]^-$: 263.1190, found: 263.1191.

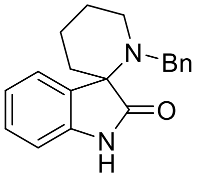

2o

1'-benzylspiro[indoline-3,2'-piperidin]-2-one 2o. White solid, 9.8 mg (from 0.10 mmol), 34% yield, m. p. 167-169 °C. ^{1}H NMR (500 MHz, $CDCl_3$) δ 7.50 (br, 1H), 7.48-7.46 (d, *J* = 7.5 Hz, 1H), 7.26-7.25 (m, 4H), 7.23-7.18 (m, 2H), 7.09-7.06 (t, *J* = 7.5 Hz, 1H), 6.83-6.82 (d, *J* = 7.5 Hz, 1H), 3.38-3.36 (d, *J* = 13.0 Hz, 1H), 3.20-3.19 (d, *J* = 13.0 Hz, 1H), 3.16-3.11 (m, 1H), 2.71-2.67 (m, 1H), 2.11-2.04 (m, 1H), 1.96-1.88 (m, 2H), 1.76-1.72 (m, 1H), 1.69-1.62 (m, 2H). ^{13}C NMR (101 MHz, $CDCl_3$) δ 180.7, 140.2, 139.4, 133.1, 128.5, 128.4, 128.0, 126.8, 124.1, 122.7, 109.7, 66.3, 56.3, 46.1, 35.3, 25.6, 19.1. IR ν_{max} (KBr, film, cm^{-1}): 3210, 3061, 3028, 2929, 2851, 1702, 1619, 1472, 754. HRMS (ESI): calcd for $C_{19}H_{19}ON_2^-$ $[M-H]^-$: 291.1503, found: 291.1504.

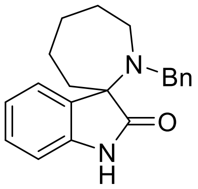

2p

1-benzylspiro[azepane-2,3'-indolin]-2'-one 2p. White solid, 4.7 mg (from 0.10 mmol), 15% yield, m. p. 177-179 °C. ^{1}H NMR (500 MHz, $CDCl_3$) δ 7.67 (s, 1H), 7.62-7.61 (d, *J* = 7.5 Hz, 1H), 7.32-7.30 (m, 2H), 7.27 (s, 1H), 7.25 (s, 1H), 7.24-7.17 (m, 2H), 7.08-7.05 (t, *J* = 7.5 Hz, 1H), 6.85-6.83 (d, *J* = 7.5 Hz, 1H), 3.55-3.50 (dd, *J* = 15.0, 10.5 Hz, 1H), 3.44-3.42 (d, *J* = 13.5 Hz, 1H), 3.24-3.21 (d, *J* = 13.0 Hz, 1H), 2.68-2.64 (dd, *J* = 15.0, 6.0 Hz, 1H), 2.19-2.05 (m, 2H), 1.92-1.84 (m, 3H), 1.60 (m, 1H) 1.47-1.40 (m, 2H). ^{13}C NMR (126 MHz, $CDCl_3$) δ 182.4, 140.3, 139.8, 134.8, 128.5, 128.2, 128.0, 126.8, 124.0, 122.7, 109.7, 69.6, 56.5, 47.2, 38.3, 32.4, 30.1, 22.7. IR ν_{max} (KBr, film, cm^{-1}): 3207, 3028, 2925, 2853, 1704, 1651, 1469, 747. HRMS (ESI): calcd for $C_{20}H_{23}ON_2^+$ $[M+H]^+$: 307.1805, found: 307.1808.

4. Reduction of 2a

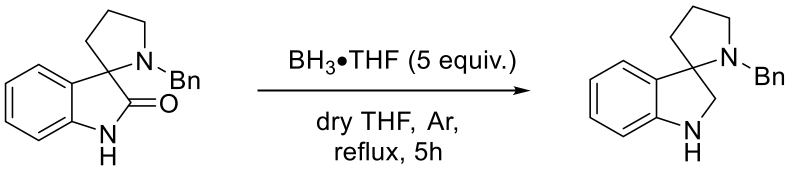

1'-benzylspiro[indoline-3,2'-pyrrolidin]-2-one **2a** (0.5 mmol, 139.0 mg) was dissolved in dry THF (10 mL), B_2H_6 (2.5 mmol, 2.5 mL, 1M in THF) was added slowly under the Ar. The mixture was heated to reflux for 5 hours. And then to the vigorously stirring mixture were added methanol (5 ml) at 0 °C. After being stirred at 0 °C 10 minutes, the mixture was warmed to room temperature and reflux for another 30 minutes. After this time, the solvent was removed under vacuum and residue was purified by silica column chromatography (elute: dichloromethane /methanol 10/1, with 1‰ NH_4OH) to afford the desired product 1'-benzylspiro[indoline-3,2'-pyrrolidine] **3** as an orange solid.

1'-benzylspiro[indoline-3,2'-pyrrolidine] 3. Orange solid, 70.0 mg (from 0.50 mmol), 53% yield, m. p. 79-81 °C. ^{1}H NMR (500 MHz, $CDCl_3$) δ 8.14 (s, 1H), 7.59-7.58 (d, *J* = 7.5 Hz, 1H), 7.31-7.30 (m, 4H), 7.25-7.23 (m, 1H), 7.18-7.15 (t, *J* = 7.5 Hz, 1H), 7.11-7.09 (t, *J* = 7.5 Hz, 1H), 6.89 (s, 1H), 3.77 (s, 2H), 2.81-2.78 (t, *J* = 7.5 Hz, 4H), 2.73 (m, 2H), 2.06 (s, 1H), 1.95-1.92 (t, *J* = 7.5 Hz, 2H). ^{13}C NMR (126 MHz, $CDCl_3$) δ 140.2, 136.3, 128.4, 128.2, 127.4, 126.9, 121.8, 121.2, 119.0, 118.8, 116.1, 111.0, 62.5, 53.9, 49.1, 30.2, 30.0, 22.8. IR ν_{max} (KBr, film, cm^{-1}): 3414, 3241, 3057, 2926, 2849, 1456, 1098, 741, 697. HRMS (ESI): calcd for $C_{18}H_{21}N_2^+$ $[M+H]^+$: 265.1699, found: 265.1695.

5. Control experiment

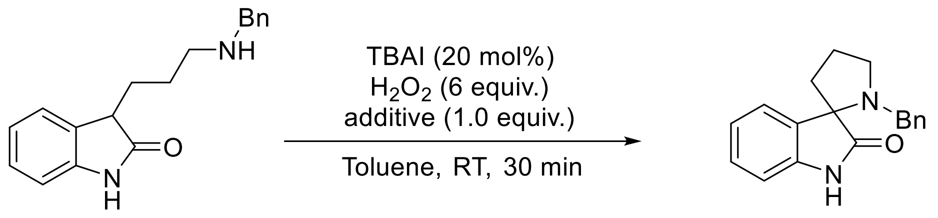

1a **2a**

entry	additive	yield
1	TEMOP	52%
2	BHT	72%
3	HQ	51%

3-(3-(benzylamino)propyl)indolin-2-one **1a** (0.10 mmol, 28.0 mg), TBAI (0.02 mmol, 7.4 mg) and additive (0.1 mmol) was dissolved in toluene (0.5 mL). 35% of H_2O_2 (0.6 mmol, 52.0 μL) was added and the reaction mixture was stirred at room temperature for 0.5 h. After that time, the mixture was quenched by saturated sodium thiosulfate solution (1 mL) and diluted with dichloromethane (10 mL). The organic layer was washed by water (10 mL * 2), dried over anhydrous sodium sulfate and evaporated to afford the crude product. The crude was purified by silica column chromatography (elute: petroleum ether/ethyl acetate 2/1) to give the pure products **2a**.

6. X-ray crystallographic data

X-ray crystallography of compound 2k (CCDC 1847718)

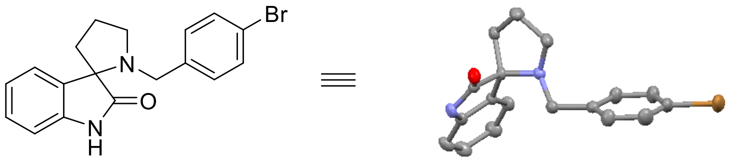

Table 1. Crystal data and structure refinement for mx6777.

Identification code	mx6777	
Empirical formula	C18 H17 Br N2 O	
Formula weight	357.24	
Temperature	173.15 K	
Wavelength	0.71073 Å	
Crystal system	Monoclinic	
Space group	P 1 21/c 1	
Unit cell dimensions	a = 9.7607(14) Å	α= 90°.
	b = 17.628(3) Å	β= 90.749(3)°.
	c = 18.635(4) Å	γ = 90°.

Volume	3206.1(10) Å^{3}
Z	8
Density (calculated)	1.480 Mg/m^{3}
Absorption coefficient	2.567 mm^{-1}
F(000)	1456
Crystal size	0.224 x 0.172 x 0.131 mm^{3}
Theta range for data collection	1.590 to 27.507°.
Index ranges	-12<=h<=12, -22<=k<=22, -24<=l<=24
Reflections collected	23623
Independent reflections	7302 [R(int) = 0.0895]
Completeness to theta = 25.242°	99.4 %
Absorption correction	None
Max. and min. transmission	1.0000 and 0.7611
Refinement method	Full-matrix least-squares on F^{2}
Data / restraints / parameters	7302 / 0 / 397
Goodness-of-fit on F^{2}	1.123
Final R indices [I>2sigma(I)]	R1 = 0.0737, wR2 = 0.1523
R indices (all data)	R1 = 0.0920, wR2 = 0.1642
Extinction coefficient	n/a
Largest diff. peak and hole	0.427 and -0.918 e.Å^{-3}

Table 2. Atomic coordinates (x 10^4) and equivalent isotropic displacement parameters (Å^{2}x 10^3) for mx6777. U(eq) is defined as one third of the trace of the orthogonalized U^{ij} tensor.

	x	y	z	U(eq)
Br1	5295(1)	10162(1)	6385(1)	49(1)
N1	1974(3)	6772(2)	6451(2)	25(1)
O1	1417(3)	5564(2)	5288(1)	26(1)
C1	1101(4)	6111(2)	6502(2)	22(1)
N2	-703(3)	5576(2)	5810(2)	26(1)
C2	658(4)	5727(2)	5787(2)	21(1)
C3	-1294(4)	5879(2)	6432(2)	23(1)
C4	-2649(4)	5853(2)	6647(2)	29(1)
C5	-2966(4)	6204(2)	7290(2)	32(1)
C6	-1963(4)	6569(2)	7704(2)	27(1)
C7	-616(4)	6583(2)	7478(2)	24(1)
C8	-276(4)	6235(2)	6842(2)	21(1)
C9	3404(4)	6520(2)	6352(2)	32(1)
C10	3435(4)	5689(2)	6595(2)	31(1)
C11	2025(4)	5551(2)	6919(2)	24(1)
C12	1523(4)	7374(2)	5971(2)	30(1)
C13	2385(4)	8076(2)	6066(2)	28(1)
C14	2887(5)	8469(2)	5487(2)	34(1)
C15	3743(5)	9097(3)	5580(2)	39(1)
C16	4082(5)	9332(2)	6262(2)	32(1)
C17	3575(6)	8962(3)	6850(2)	44(1)
C18	2714(6)	8336(3)	6755(2)	44(1)
Br1A	-74(1)	-88(1)	3548(1)	56(1)
C1A	3969(4)	3266(2)	5857(2)	21(1)
O1A	3564(3)	4384(2)	5069(1)	28(1)
N1A	3084(3)	2720(2)	5490(2)	24(1)
C2A	4359(4)	3982(2)	5413(2)	24(1)

N2A	5735(3)	4088(2)	5482(2)	25(1)
C3A	6356(4)	3480(2)	5850(2)	22(1)
C4A	7733(4)	3354(3)	5982(2)	32(1)
C5A	8092(4)	2676(3)	6323(2)	35(1)
C6A	7103(5)	2151(3)	6528(2)	36(1)
C7A	5719(4)	2301(2)	6403(2)	27(1)
C8A	5361(4)	2965(2)	6058(2)	21(1)
C9A	1660(4)	2993(2)	5527(2)	30(1)
C10A	1641(4)	3535(3)	6167(2)	37(1)
C11A	3076(4)	3513(2)	6490(2)	27(1)
C12A	3533(4)	2500(2)	4775(2)	27(1)
C13A	2683(4)	1859(2)	4472(2)	27(1)
C14A	2184(5)	1890(3)	3775(2)	35(1)
C15A	1365(5)	1308(3)	3499(2)	41(1)
C16A	1062(5)	701(3)	3925(2)	37(1)
C17A	1553(5)	646(3)	4616(2)	40(1)
C18A	2369(5)	1223(2)	4889(2)	36(1)

Table 3. Bond lengths [Å] and angles [°] for mx6777.

Br1-C16	1.894(4)
N1-C1	1.448(5)
N1-C9	1.478(5)
N1-C12	1.453(5)
O1-C2	1.231(4)
C1-C2	1.551(5)
C1-C8	1.509(5)
C1-C11	1.540(5)
N2-H2	0.8800
N2-C2	1.355(5)
N2-C3	1.408(5)
C3-C4	1.387(5)
C3-C8	1.394(5)
C4-H4	0.9500
C4-C5	1.388(6)
C5-H5	0.9500
C5-C6	1.396(6)
C6-H6	0.9500
C6-C7	1.386(6)
C7-H7	0.9500
C7-C8	1.380(5)
C9-H9A	0.9900
C9-H9B	0.9900
C9-C10	1.534(6)
C10-H10A	0.9900
C10-H10B	0.9900
C10-C11	1.529(6)
C11-H11A	0.9900
C11-H11B	0.9900
C12-H12A	0.9900

C12-H12B	0.9900
C12-C13	1.505(6)
C13-C14	1.379(6)
C13-C18	1.395(6)
C14-H14	0.9500
C14-C15	1.397(6)
C15-H15	0.9500
C15-C16	1.372(6)
C16-C17	1.373(6)
C17-H17	0.9500
C17-C18	1.397(6)
C18-H18	0.9500
Br1A-C16A	1.907(4)
C1A-N1A	1.457(5)
C1A-C2A	1.559(5)
C1A-C8A	1.502(5)
C1A-C11A	1.539(5)
O1A-C2A	1.226(4)
N1A-C9A	1.473(5)
N1A-C12A	1.461(5)
C2A-N2A	1.360(5)
N2A-H2A	0.8800
N2A-C3A	1.406(5)
C3A-C4A	1.381(5)
C3A-C8A	1.388(5)
C4A-H4A	0.9500
C4A-C5A	1.398(6)
C5A-H5A	0.9500
C5A-C6A	1.394(6)
C6A-H6A	0.9500
C6A-C7A	1.393(6)
C7A-H7A	0.9500

C7A-C8A	1.378(5)
C9A-H9AA	0.9900
C9A-H9AB	0.9900
C9A-C10A	1.529(6)
C10A-H10C	0.9900
C10A-H10D	0.9900
C10A-C11A	1.517(6)
C11A-H11C	0.9900
C11A-H11D	0.9900
C12A-H12C	0.9900
C12A-H12D	0.9900
C12A-C13A	1.507(5)
C13A-C14A	1.384(6)
C13A-C18A	1.399(6)
C14A-H14A	0.9500
C14A-C15A	1.394(6)
C15A-H15A	0.9500
C15A-C16A	1.367(7)
C16A-C17A	1.373(6)
C17A-H17A	0.9500
C17A-C18A	1.384(6)
C18A-H18A	0.9500

C1-N1-C9	108.9(3)
C1-N1-C12	117.0(3)
C12-N1-C9	115.0(3)
N1-C1-C2	117.0(3)
N1-C1-C8	116.0(3)
N1-C1-C11	101.9(3)
C8-C1-C2	100.6(3)
C8-C1-C11	113.6(3)
C11-C1-C2	107.9(3)

C2-N2-H2	124.3
C2-N2-C3	111.4(3)
C3-N2-H2	124.3
O1-C2-C1	125.9(3)
O1-C2-N2	125.3(3)
N2-C2-C1	108.7(3)
C4-C3-N2	128.8(3)
C4-C3-C8	122.2(3)
C8-C3-N2	108.9(3)
C3-C4-H4	121.4
C3-C4-C5	117.3(4)
C5-C4-H4	121.4
C4-C5-H5	119.3
C4-C5-C6	121.4(4)
C6-C5-H5	119.3
C5-C6-H6	119.9
C7-C6-C5	120.1(4)
C7-C6-H6	119.9
C6-C7-H7	120.2
C8-C7-C6	119.5(3)
C8-C7-H7	120.2
C3-C8-C1	109.7(3)
C7-C8-C1	130.6(3)
C7-C8-C3	119.5(4)
N1-C9-H9A	110.7
N1-C9-H9B	110.7
N1-C9-C10	105.4(3)
H9A-C9-H9B	108.8
C10-C9-H9A	110.7
C10-C9-H9B	110.7
C9-C10-H10A	110.8
C9-C10-H10B	110.8

H10A-C10-H10B	108.9
C11-C10-C9	104.7(3)
C11-C10-H10A	110.8
C11-C10-H10B	110.8
C1-C11-H11A	111.2
C1-C11-H11B	111.2
C10-C11-C1	102.9(3)
C10-C11-H11A	111.2
C10-C11-H11B	111.2
H11A-C11-H11B	109.1
N1-C12-H12A	109.4
N1-C12-H12B	109.4
N1-C12-C13	111.3(3)
H12A-C12-H12B	108.0
C13-C12-H12A	109.4
C13-C12-H12B	109.4
C14-C13-C12	121.6(4)
C14-C13-C18	118.3(4)
C18-C13-C12	120.0(4)
C13-C14-H14	119.4
C13-C14-C15	121.3(4)
C15-C14-H14	119.4
C14-C15-H15	120.3
C16-C15-C14	119.4(4)
C16-C15-H15	120.3
C15-C16-Br1	119.2(3)
C15-C16-C17	120.7(4)
C17-C16-Br1	120.1(3)
C16-C17-H17	120.1
C16-C17-C18	119.7(4)
C18-C17-H17	120.1
C13-C18-C17	120.5(4)

C13-C18-H18	119.7
C17-C18-H18	119.7
N1A-C1A-C2A	115.7(3)
N1A-C1A-C8A	114.4(3)
N1A-C1A-C11A	102.0(3)
C8A-C1A-C2A	101.1(3)
C8A-C1A-C11A	115.4(3)
C11A-C1A-C2A	108.8(3)
C1A-N1A-C9A	108.5(3)
C1A-N1A-C12A	114.8(3)
C12A-N1A-C9A	115.0(3)
O1A-C2A-C1A	126.0(4)
O1A-C2A-N2A	126.0(4)
N2A-C2A-C1A	108.0(3)
C2A-N2A-H2A	124.5
C2A-N2A-C3A	111.1(3)
C3A-N2A-H2A	124.5
C4A-C3A-N2A	128.4(4)
C4A-C3A-C8A	121.9(4)
C8A-C3A-N2A	109.7(3)
C3A-C4A-H4A	121.4
C3A-C4A-C5A	117.1(4)
C5A-C4A-H4A	121.4
C4A-C5A-H5A	119.3
C6A-C5A-C4A	121.5(4)
C6A-C5A-H5A	119.3
C5A-C6A-H6A	120.0
C7A-C6A-C5A	120.1(4)
C7A-C6A-H6A	120.0
C6A-C7A-H7A	120.7
C8A-C7A-C6A	118.6(4)
C8A-C7A-H7A	120.7

C3A-C8A-C1A	109.5(3)
C7A-C8A-C1A	129.8(3)
C7A-C8A-C3A	120.8(4)
N1A-C9A-H9AA	110.7
N1A-C9A-H9AB	110.7
N1A-C9A-C10A	105.1(3)
H9AA-C9A-H9AB	108.8
C10A-C9A-H9AA	110.7
C10A-C9A-H9AB	110.7
C9A-C10A-H10C	110.6
C9A-C10A-H10D	110.6
H10C-C10A-H10D	108.7
C11A-C10A-C9A	105.8(3)
C11A-C10A-H10C	110.6
C11A-C10A-H10D	110.6
C1A-C11A-H11C	111.1
C1A-C11A-H11D	111.1
C10A-C11A-C1A	103.5(3)
C10A-C11A-H11C	111.1
C10A-C11A-H11D	111.1
H11C-C11A-H11D	109.0
N1A-C12A-H12C	109.3
N1A-C12A-H12D	109.3
N1A-C12A-C13A	111.7(3)
H12C-C12A-H12D	107.9
C13A-C12A-H12C	109.3
C13A-C12A-H12D	109.3
C14A-C13A-C12A	120.5(4)
C14A-C13A-C18A	118.4(4)
C18A-C13A-C12A	121.1(4)
C13A-C14A-H14A	119.7
C13A-C14A-C15A	120.7(4)

C15A-C14A-H14A	119.7
C14A-C15A-H15A	120.3
C16A-C15A-C14A	119.3(4)
C16A-C15A-H15A	120.3
C15A-C16A-Br1A	119.1(3)
C15A-C16A-C17A	121.6(4)
C17A-C16A-Br1A	119.2(4)
C16A-C17A-H17A	120.5
C16A-C17A-C18A	119.0(4)
C18A-C17A-H17A	120.5
C13A-C18A-H18A	119.5
C17A-C18A-C13A	121.0(4)
C17A-C18A-H18A	119.5

Symmetry transformations used to generate equivalent atoms:

Table 4. Anisotropic displacement parameters ($Å^2$x 10^3) for mx6777. The anisotropic displacement factor exponent takes the form: -2 ^{2}[h^2 $a^{*2}U^{11}$ + ... + 2 h k a* b* U^{12}]

	U^{11}	U^{22}	U^{33}	U^{23}	U^{13}	U^{12}
Br1	57(1)	36(1)	53(1)	-12(1)	17(1)	-20(1)
N1	24(2)	22(2)	29(2)	-2(1)	1(1)	-4(1)
O1	29(2)	25(1)	24(1)	-5(1)	2(1)	-2(1)
C1	23(2)	20(2)	22(2)	-3(1)	-1(1)	-1(2)
N2	25(2)	33(2)	19(2)	-4(1)	-3(1)	-7(2)
C2	26(2)	19(2)	19(2)	0(1)	-1(1)	2(2)
C3	27(2)	26(2)	16(2)	0(1)	3(1)	-4(2)
C4	24(2)	38(2)	25(2)	-2(2)	-1(2)	-9(2)
C5	29(2)	37(2)	30(2)	-2(2)	5(2)	-4(2)
C6	33(2)	27(2)	23(2)	-2(2)	3(2)	4(2)
C7	27(2)	23(2)	22(2)	-1(1)	-4(2)	-1(2)
C8	25(2)	18(2)	20(2)	0(1)	1(1)	-1(2)
C9	24(2)	34(2)	38(2)	-3(2)	4(2)	-6(2)
C10	26(2)	33(2)	34(2)	1(2)	-1(2)	-1(2)
C11	26(2)	23(2)	23(2)	-2(1)	-5(2)	-1(2)
C12	34(2)	29(2)	26(2)	3(2)	-1(2)	-3(2)
C13	33(2)	24(2)	25(2)	-3(2)	2(2)	2(2)
C14	45(3)	31(2)	25(2)	-1(2)	2(2)	-4(2)
C15	50(3)	36(2)	30(2)	5(2)	11(2)	-8(2)
C16	38(2)	20(2)	39(2)	-3(2)	5(2)	-6(2)
C17	70(4)	35(3)	28(2)	-1(2)	4(2)	-14(2)
C18	68(4)	33(2)	30(2)	2(2)	7(2)	-21(2)
Br1A	57(1)	59(1)	51(1)	-28(1)	12(1)	-28(1)
C1A	22(2)	18(2)	24(2)	0(1)	-2(1)	-3(2)
O1A	30(2)	23(1)	32(2)	6(1)	-7(1)	-1(1)
N1A	21(2)	25(2)	27(2)	-1(1)	2(1)	-1(1)
C2A	26(2)	23(2)	23(2)	-4(2)	-4(2)	2(2)

N2A	27(2)	22(2)	27(2)	6(1)	0(1)	-8(1)
C3A	25(2)	25(2)	16(2)	2(1)	-2(1)	-2(2)
C4A	21(2)	43(2)	30(2)	10(2)	-1(2)	-5(2)
C5A	24(2)	50(3)	31(2)	11(2)	-6(2)	4(2)
C6A	34(2)	36(2)	36(2)	12(2)	-1(2)	2(2)
C7A	29(2)	25(2)	26(2)	7(2)	0(2)	-2(2)
C8A	20(2)	21(2)	21(2)	0(1)	-1(1)	-3(2)
C9A	22(2)	31(2)	38(2)	-4(2)	-2(2)	-2(2)
C10A	23(2)	41(3)	46(3)	-8(2)	2(2)	2(2)
C11A	25(2)	25(2)	31(2)	-2(2)	0(2)	-5(2)
C12A	26(2)	27(2)	29(2)	-1(2)	3(2)	-3(2)
C13A	25(2)	26(2)	29(2)	-3(2)	5(2)	1(2)
C14A	43(3)	32(2)	30(2)	3(2)	-4(2)	-3(2)
C15A	46(3)	50(3)	26(2)	-5(2)	-4(2)	-7(2)
C16A	35(2)	38(2)	37(2)	-14(2)	6(2)	-11(2)
C17A	48(3)	32(2)	40(2)	-5(2)	3(2)	-12(2)
C18A	44(3)	34(2)	29(2)	-3(2)	-1(2)	-8(2)

Table 5. Hydrogen coordinates (x 10^4) and isotropic displacement parameters ($Å^2$x 10^3) for mx6777.

	x	y	z	U(eq)
H2	-1158	5322	5478	31
H4	-3331	5604	6365	35
H5	-3885	6195	7452	38
H6	-2204	6808	8141	33
H7	68	6830	7760	29
H9A	4042	6830	6647	38
H9B	3666	6563	5842	38
H10A	3589	5347	6183	37
H10B	4170	5605	6958	37
H11A	2030	5664	7439	29
H11B	1723	5021	6842	29
H12A	553	7498	6068	36
H12B	1581	7197	5468	36
H14	2647	8309	5015	41
H15	4088	9360	5176	46
H17	3809	9131	7320	53
H18	2351	8085	7161	53
H2A	6178	4485	5317	30
H4A	8406	3714	5846	38
H5A	9031	2569	6417	42
H6A	7374	1690	6753	43
H7A	5037	1953	6553	32
H9AA	1021	2565	5601	36
H9AB	1394	3260	5079	36
H10C	1406	4056	6008	44
H10D	960	3368	6523	44

H11C	3136	3142	6888	33
H11D	3355	4018	6670	33
H12C	4506	2342	4800	33
H12D	3464	2943	4450	33
H14A	2401	2312	3480	42
H15A	1022	1333	3021	49
H17A	1336	218	4904	48
H18A	2720	1187	5365	43

Table 6. Torsion angles [°] for mx6777.

Br1-C16-C17-C18	-178.1(4)
N1-C1-C2-O1	47.3(5)
N1-C1-C2-N2	-134.5(3)
N1-C1-C8-C3	134.7(3)
N1-C1-C8-C7	-49.9(5)
N1-C1-C11-C10	-39.7(3)
N1-C9-C10-C11	-7.4(4)
N1-C12-C13-C14	134.0(4)
N1-C12-C13-C18	-44.8(5)
C1-N1-C9-C10	-18.6(4)
C1-N1-C12-C13	170.1(3)
N2-C3-C4-C5	-179.7(4)
N2-C3-C8-C1	-4.6(4)
N2-C3-C8-C7	179.4(3)
C2-C1-C8-C3	7.4(4)
C2-C1-C8-C7	-177.2(4)
C2-C1-C11-C10	84.1(3)
C2-N2-C3-C4	179.4(4)
C2-N2-C3-C8	-0.9(4)
C3-N2-C2-O1	-176.0(4)
C3-N2-C2-C1	5.8(4)
C3-C4-C5-C6	0.0(6)
C4-C3-C8-C1	175.2(3)
C4-C3-C8-C7	-0.8(6)
C4-C5-C6-C7	-0.4(6)
C5-C6-C7-C8	0.2(6)
C6-C7-C8-C1	-174.6(4)
C6-C7-C8-C3	0.4(6)
C8-C1-C2-O1	173.9(4)
C8-C1-C2-N2	-7.9(4)

C8-C1-C11-C10	-165.3(3)
C8-C3-C4-C5	0.6(6)
C9-N1-C1-C2	-80.9(4)
C9-N1-C1-C8	160.4(3)
C9-N1-C1-C11	36.5(4)
C9-N1-C12-C13	-60.3(4)
C9-C10-C11-C1	28.7(4)
C11-C1-C2-O1	-66.8(5)
C11-C1-C2-N2	111.3(3)
C11-C1-C8-C3	-107.6(4)
C11-C1-C8-C7	67.7(5)
C12-N1-C1-C2	51.5(4)
C12-N1-C1-C8	-67.1(4)
C12-N1-C1-C11	168.9(3)
C12-N1-C9-C10	-152.1(3)
C12-C13-C14-C15	-176.8(4)
C12-C13-C18-C17	176.5(4)
C13-C14-C15-C16	-0.5(7)
C14-C13-C18-C17	-2.4(7)
C14-C15-C16-Br1	177.8(4)
C14-C15-C16-C17	-0.8(7)
C15-C16-C17-C18	0.5(8)
C16-C17-C18-C13	1.1(8)
C18-C13-C14-C15	2.1(7)
Br1A-C16A-C17A-C18A	-179.5(4)
C1A-N1A-C9A-C10A	-22.2(4)
C1A-N1A-C12A-C13A	172.2(3)
C1A-C2A-N2A-C3A	6.9(4)
O1A-C2A-N2A-C3A	-173.8(4)
N1A-C1A-C2A-O1A	48.2(5)
N1A-C1A-C2A-N2A	-132.5(3)
N1A-C1A-C8A-C3A	132.1(3)

N1A-C1A-C8A-C7A	-48.8(5)
N1A-C1A-C11A-C10A	-37.5(4)
N1A-C9A-C10A-C11A	-2.6(5)
N1A-C12A-C13A-C14A	133.7(4)
N1A-C12A-C13A-C18A	-45.7(5)
C2A-C1A-N1A-C9A	-80.5(4)
C2A-C1A-N1A-C12A	49.7(4)
C2A-C1A-C8A-C3A	7.1(4)
C2A-C1A-C8A-C7A	-173.8(4)
C2A-C1A-C11A-C10A	85.3(4)
C2A-N2A-C3A-C4A	175.2(4)
C2A-N2A-C3A-C8A	-2.3(4)
N2A-C3A-C4A-C5A	-176.0(4)
N2A-C3A-C8A-C1A	-3.5(4)
N2A-C3A-C8A-C7A	177.3(3)
C3A-C4A-C5A-C6A	-0.6(7)
C4A-C3A-C8A-C1A	178.7(4)
C4A-C3A-C8A-C7A	-0.5(6)
C4A-C5A-C6A-C7A	-0.9(7)
C5A-C6A-C7A-C8A	1.7(6)
C6A-C7A-C8A-C1A	179.9(4)
C6A-C7A-C8A-C3A	-1.0(6)
C8A-C1A-N1A-C9A	162.6(3)
C8A-C1A-N1A-C12A	-67.2(4)
C8A-C1A-C2A-O1A	172.4(4)
C8A-C1A-C2A-N2A	-8.4(4)
C8A-C1A-C11A-C10A	-162.0(3)
C8A-C3A-C4A-C5A	1.3(6)
C9A-N1A-C12A-C13A	-60.9(4)
C9A-C10A-C11A-C1A	24.6(4)
C11A-C1A-N1A-C9A	37.4(4)
C11A-C1A-N1A-C12A	167.6(3)

C11A-C1A-C2A-O1A	-65.8(5)
C11A-C1A-C2A-N2A	113.4(3)
C11A-C1A-C8A-C3A	-110.1(4)
C11A-C1A-C8A-C7A	69.0(5)
C12A-N1A-C9A-C10A	-152.2(3)
C12A-C13A-C14A-C15A	-178.2(4)
C12A-C13A-C18A-C17A	178.0(4)
C13A-C14A-C15A-C16A	-0.3(7)
C14A-C13A-C18A-C17A	-1.4(7)
C14A-C15A-C16A-Br1A	179.4(4)
C14A-C15A-C16A-C17A	-0.6(8)
C15A-C16A-C17A-C18A	0.4(7)
C16A-C17A-C18A-C13A	0.6(7)
C18A-C13A-C14A-C15A	1.2(7)

Symmetry transformations used to generate equivalent atoms:

Table 7. Hydrogen bonds for mx6777 [Å and °].

D-H...A	d(D-H)	d(H...A)	d(D...A)	<(DHA)
N2-H2...O1#1	0.88	2.13	2.946(4)	154.1
N2-H2...O1A#1	0.88	2.60	3.220(4)	128.3
N2A-H2A...O1#2	0.88	2.62	3.203(4)	124.8
N2A-H2A...O1A#2	0.88	2.13	2.965(4)	157.0

Symmetry transformations used to generate equivalent atoms:

#1 -x,-y+1,-z+1 #2 -x+1,-y+1,-z+1

7. NMR spectra

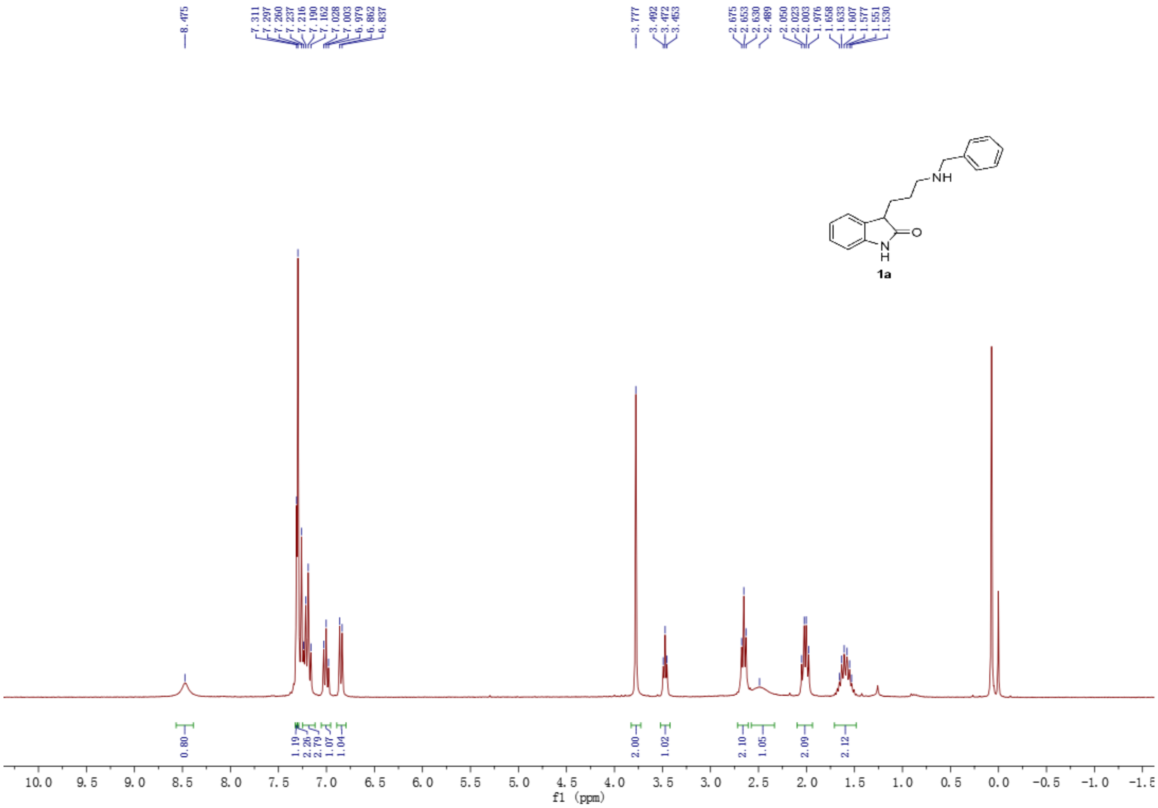

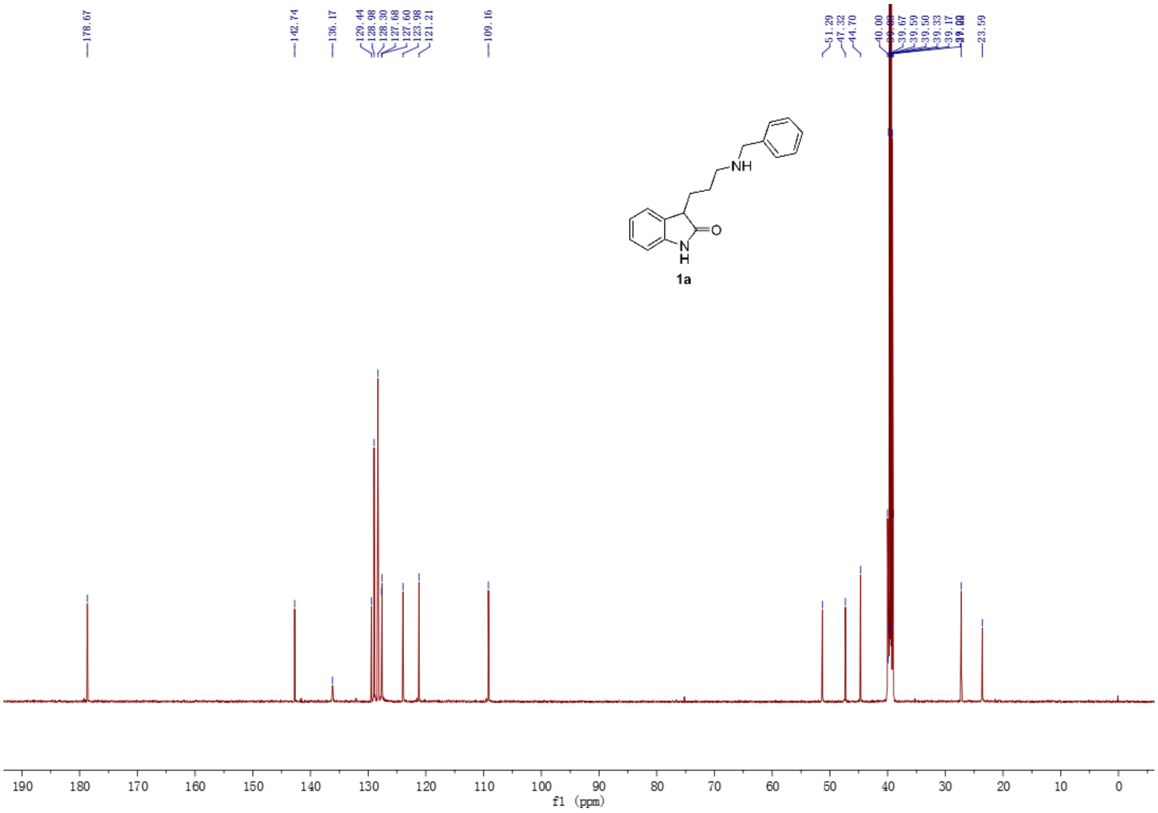

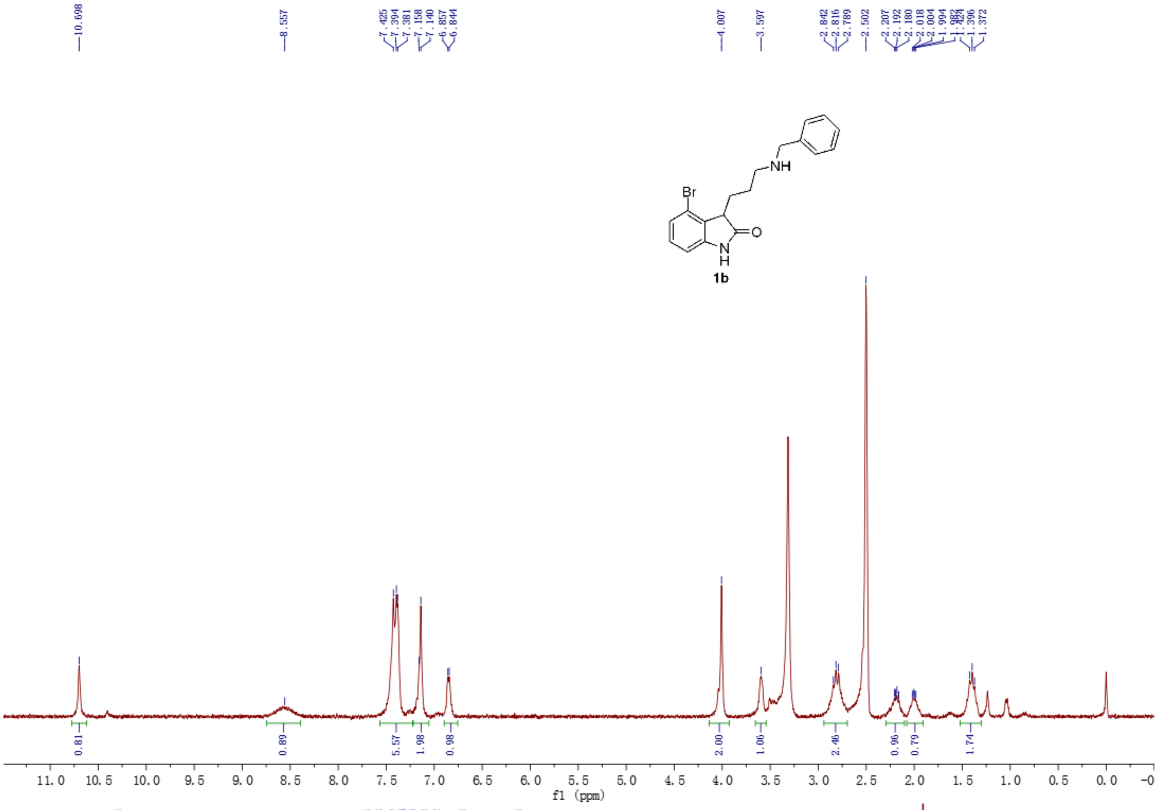
10.698
8.557
7.425
7.394
7.381
7.158
7.140
6.857
6.844
4.007
3.597
2.842
2.816
2.789
2.502
2.207
2.192
2.180
2.018
2.004
1.994
1.396
1.372
Br
NH
O
H
N
1b
0.81
0.89
5.57
1.98
0.98
2.00
1.06
2.46
0.96
0.79
1.74
f1 (ppm)

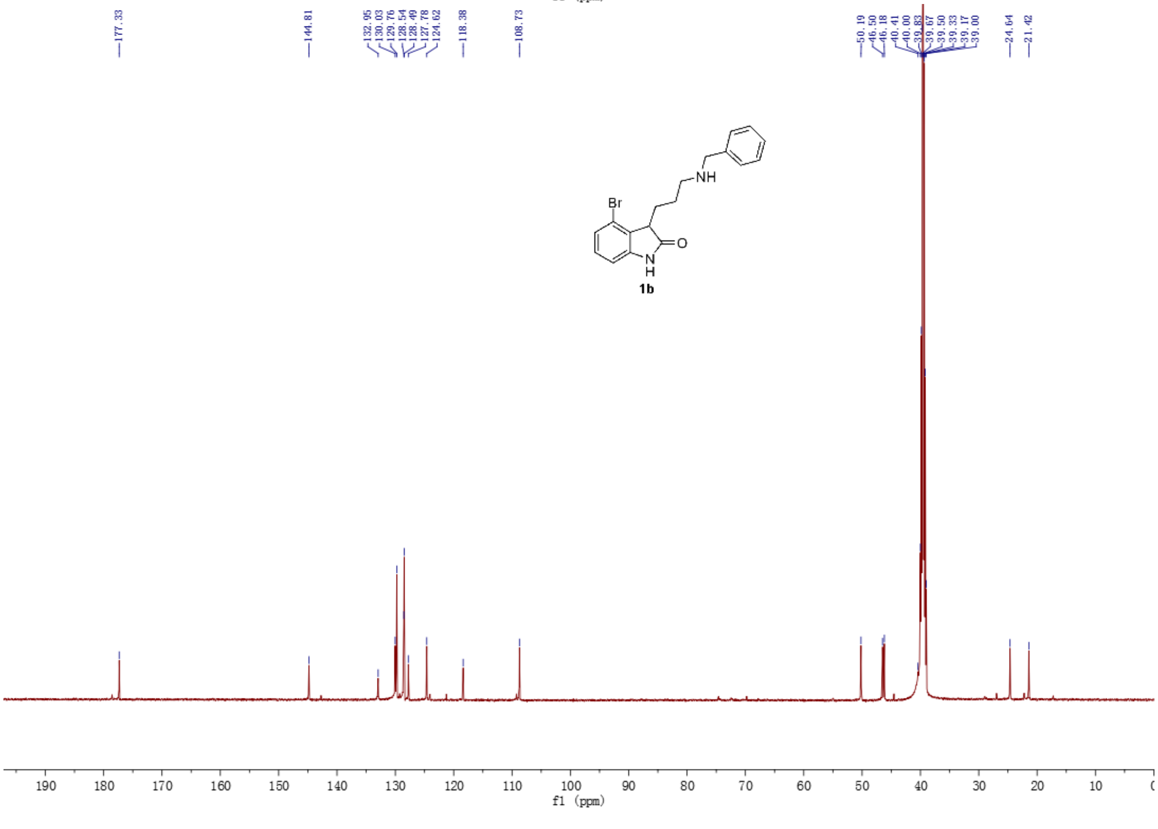
177.33
144.81
132.95
130.03
129.76
128.54
128.49
127.78
124.62
118.38
108.73
50.19
46.50
46.18
40.41
40.00
39.67
39.50
39.33
39.17
39.00
24.64
21.42
Br
NH
O
H
N
1b
f1 (ppm)

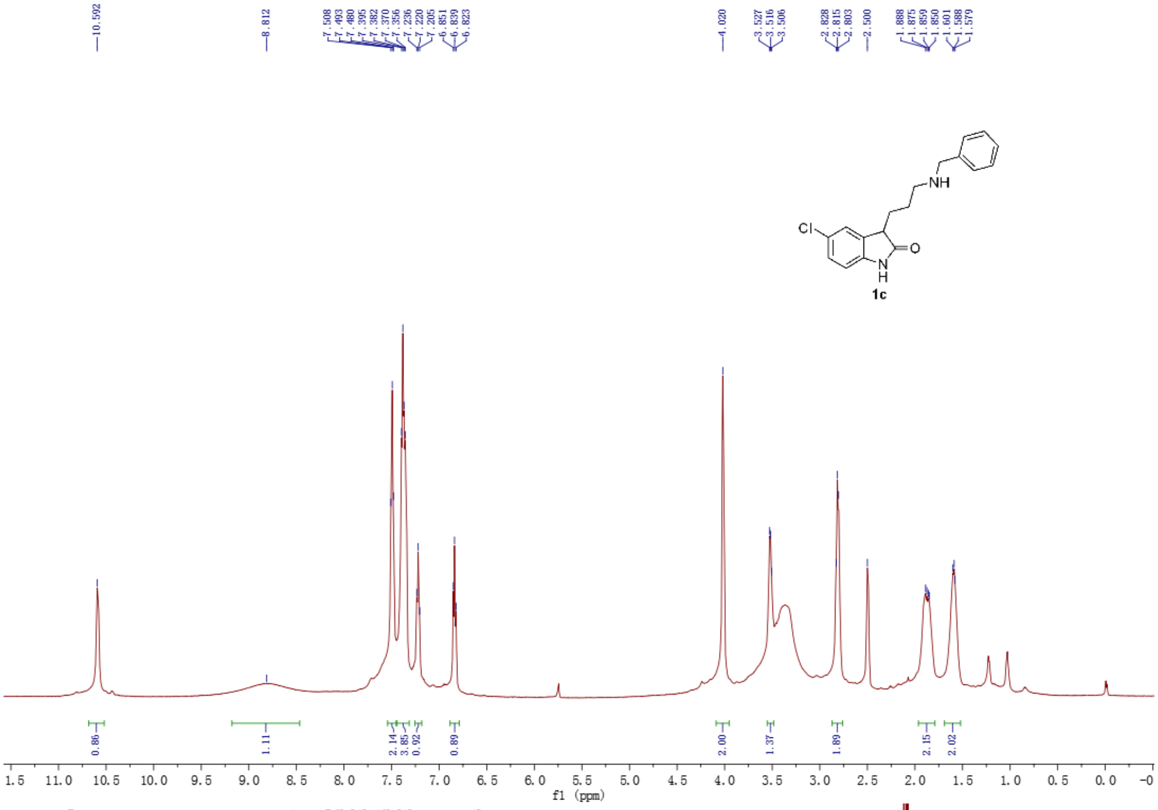

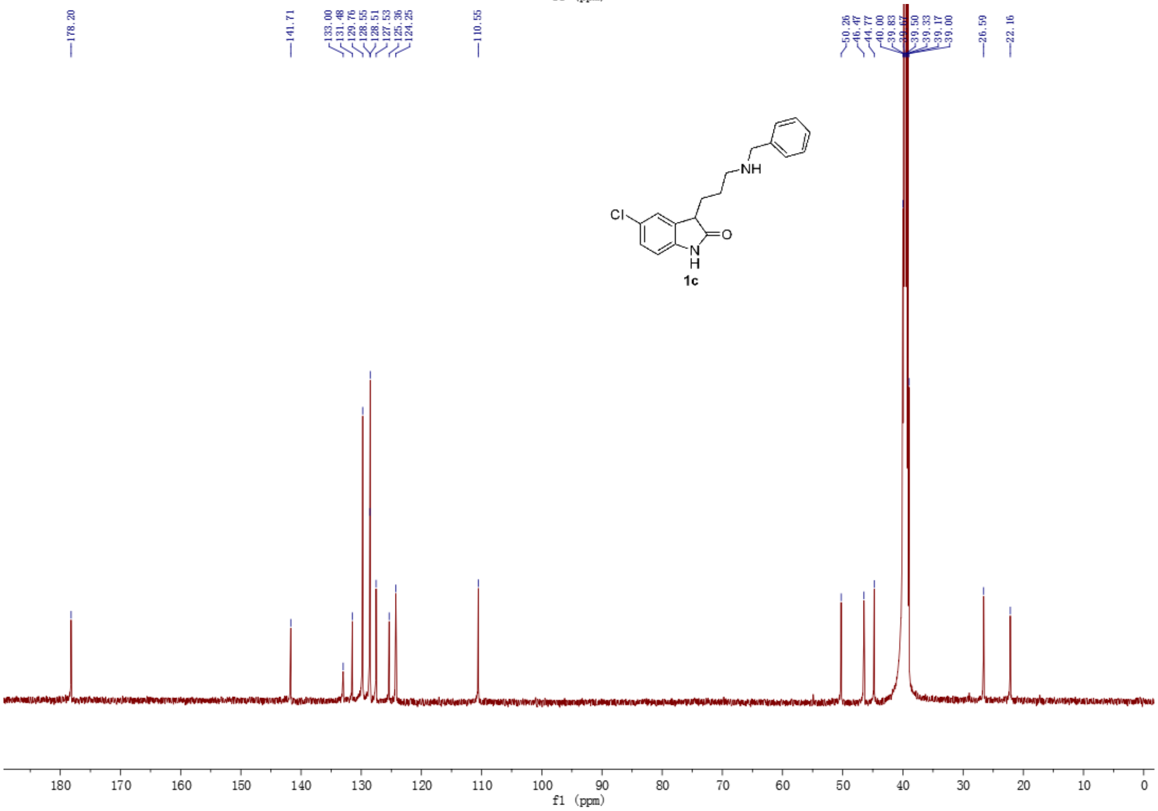

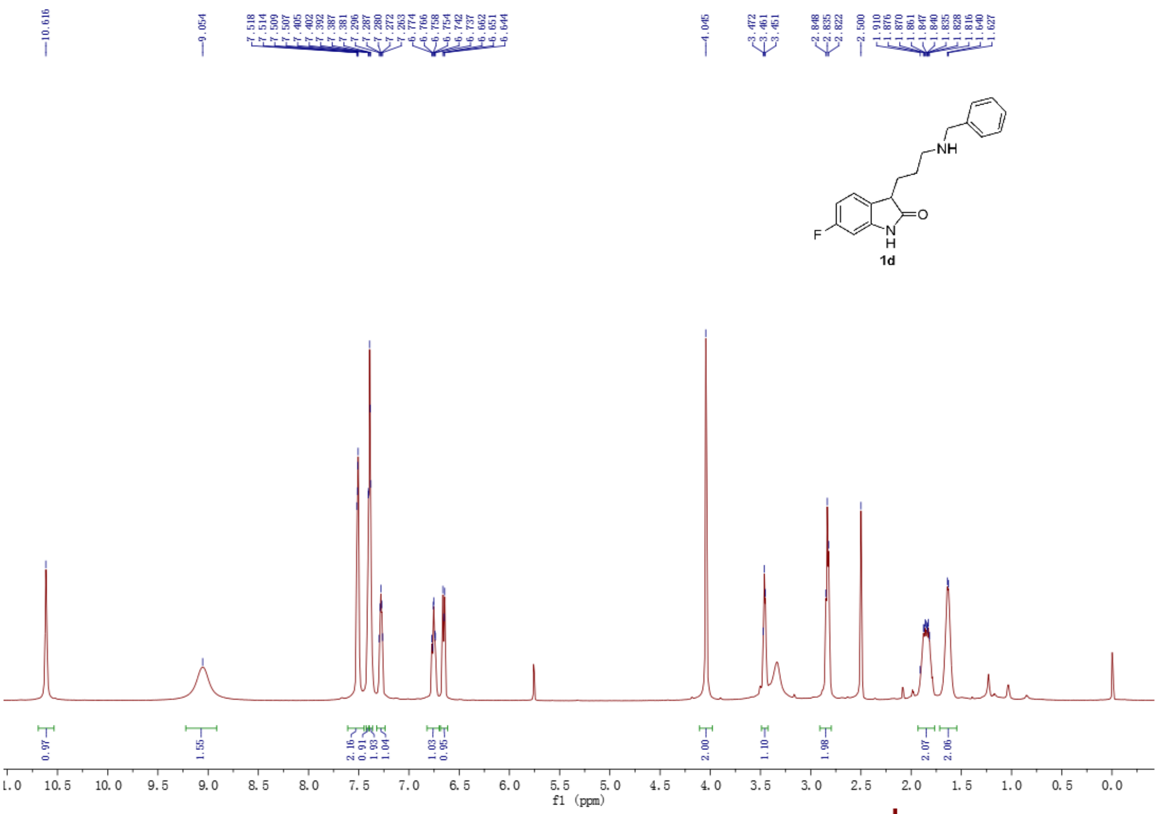

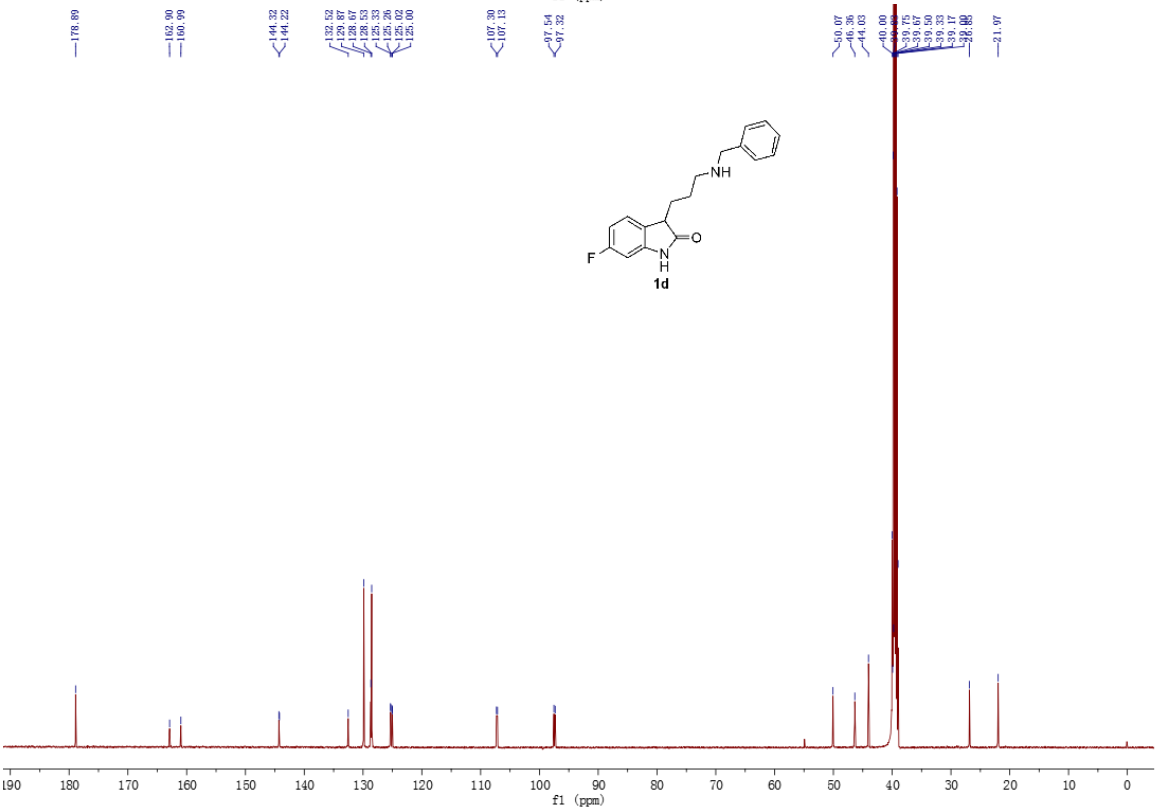

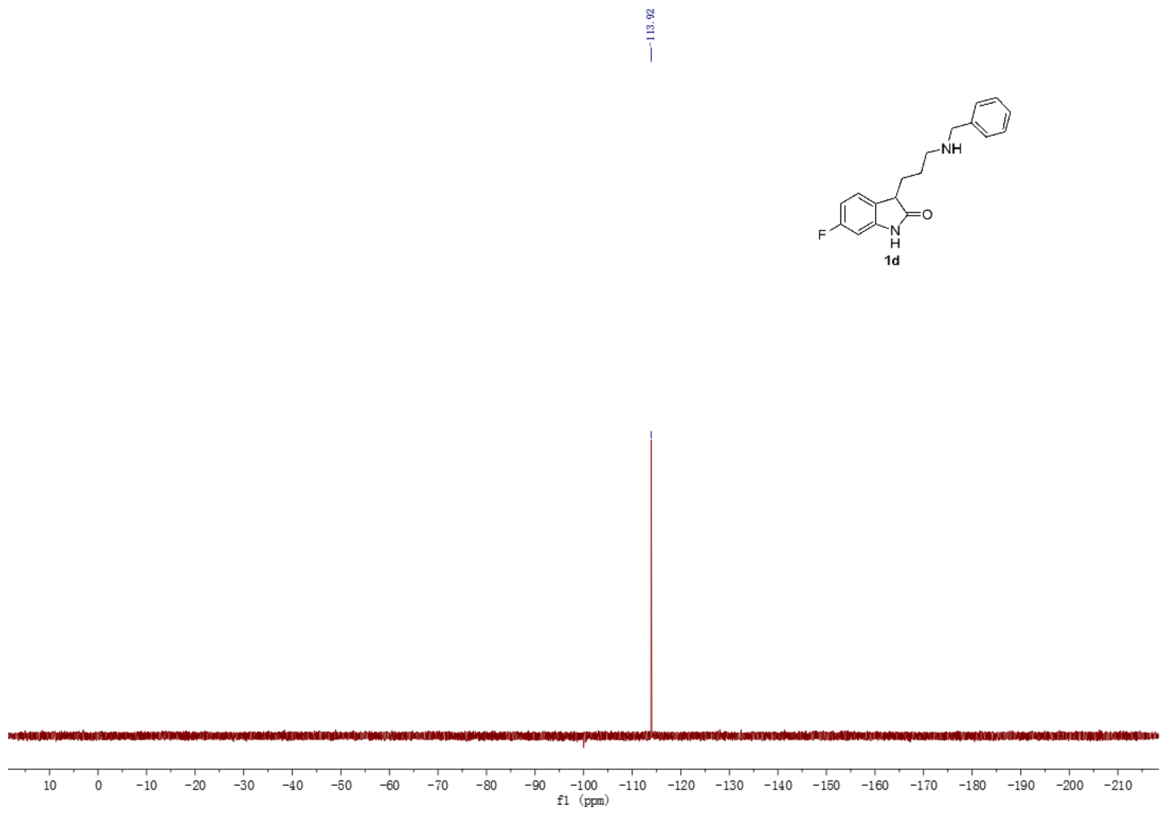
-113.92
NH
F
N
H
O
1d
f1 (ppm)

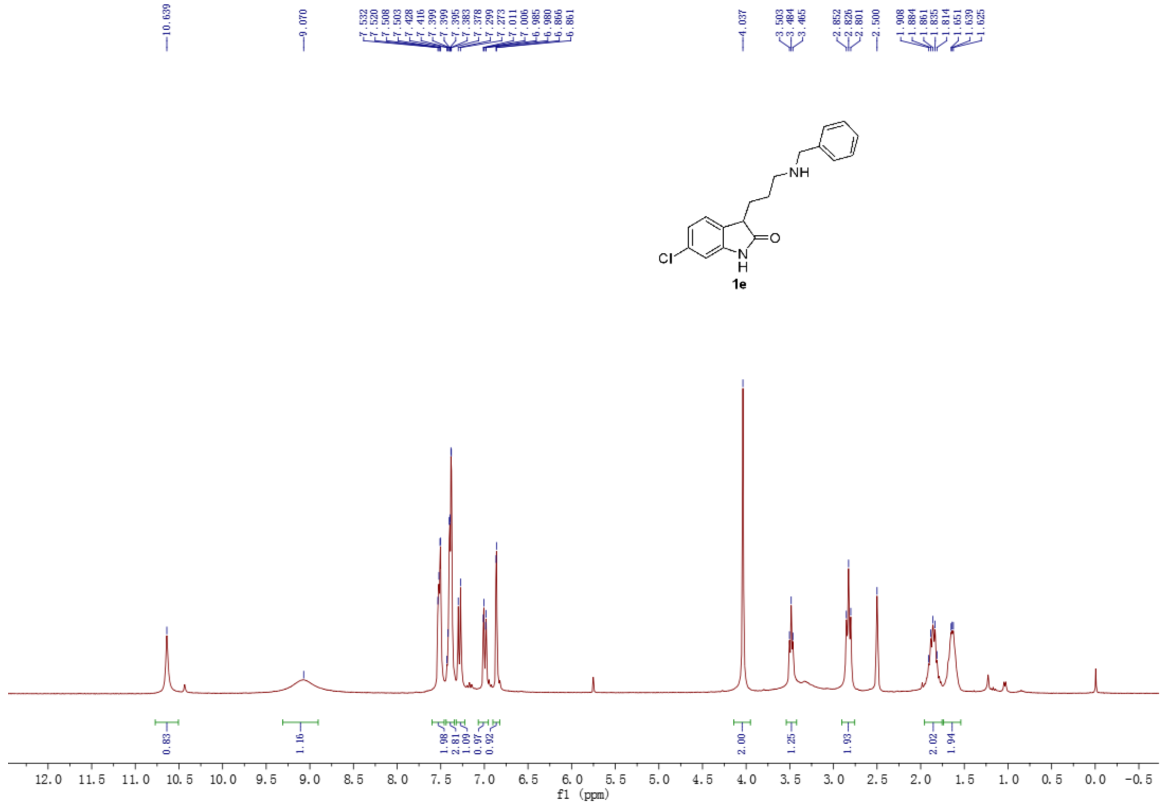
10.639
9.070
7.532
7.520
7.508
7.503
7.428
7.416
7.399
7.399
7.395
7.383
7.378
7.299
7.273
7.011
7.006
6.985
6.980
6.866
6.861
4.037
3.503
3.484
3.465
2.852
2.826
2.801
2.500
1.908
1.884
1.861
1.835
1.814
1.651
1.639
1.625
NH
Cl
N
H
O
1e
0.83
1.16
1.98
2.81
1.09
0.97
0.92
2.00
1.25
1.93
2.02
1.94
f1 (ppm)

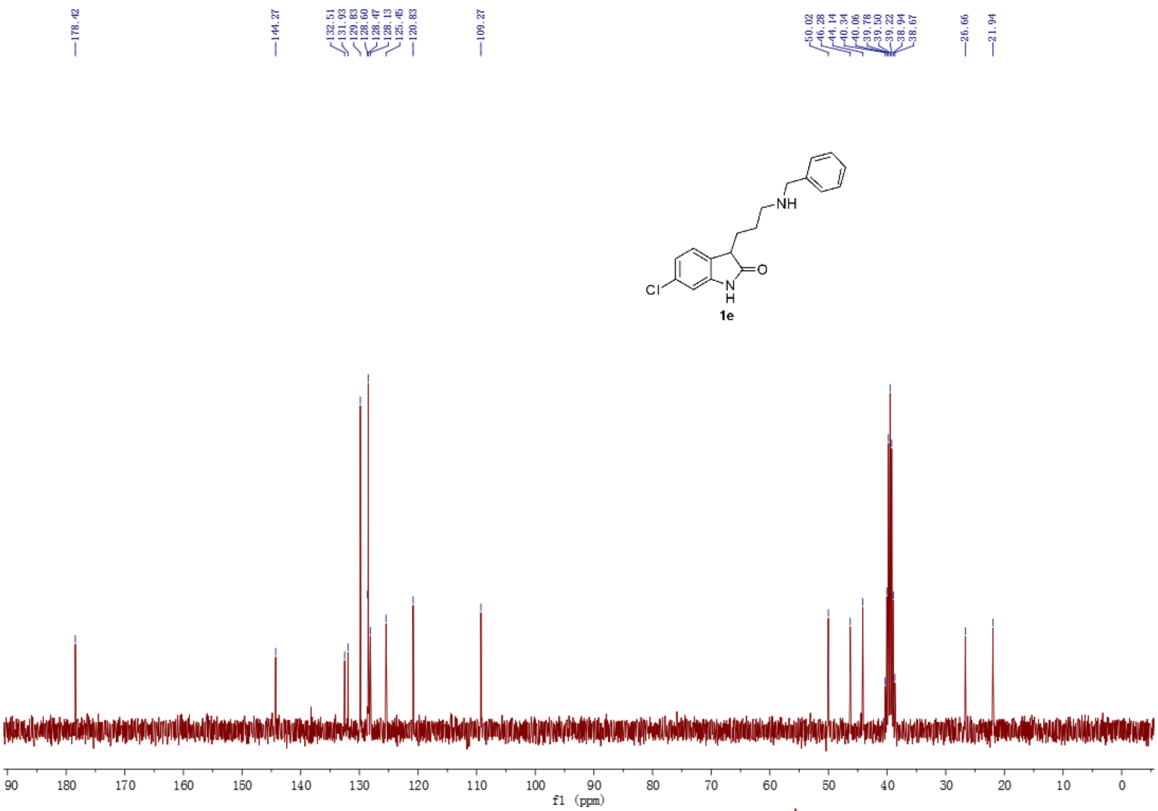
178.42
144.27
132.51
131.93
129.83
128.60
128.47
128.13
125.45
120.83
109.27
50.02
46.28
44.14
40.34
40.06
39.78
39.50
39.22
38.94
38.67
26.66
21.94
NH
O
Cl
N
H
1e
f1 (ppm)

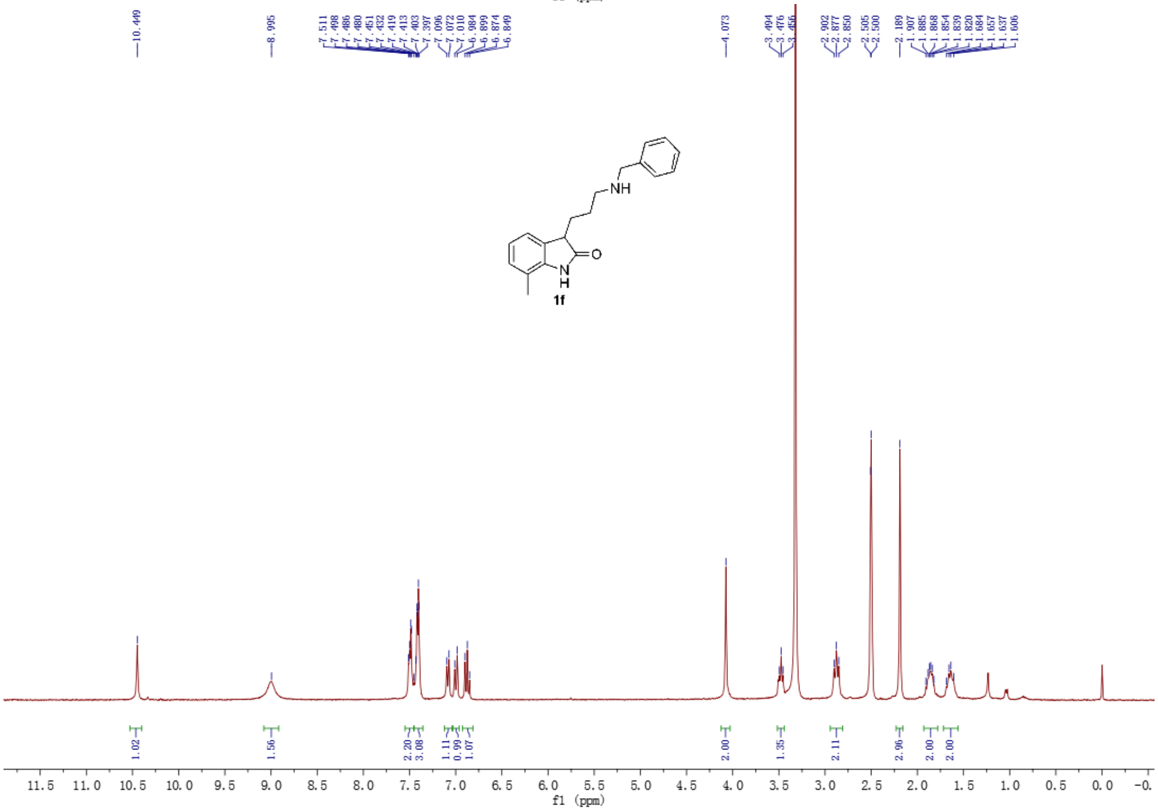
10.449
8.995
7.511
7.498
7.486
7.480
7.451
7.432
7.419
7.413
7.403
7.397
7.096
7.072
7.010
6.984
6.899
6.874
6.849
4.073
3.494
3.476
2.902
2.877
2.850
2.505
2.500
2.189
1.907
1.885
1.868
1.854
1.839
1.820
1.684
1.657
1.637
1.606
NH
O
N
H
1f
1.02
1.56
2.20
3.08
1.11
0.99
1.07
2.00
1.35
2.11
2.96
2.00
2.00
f1 (ppm)

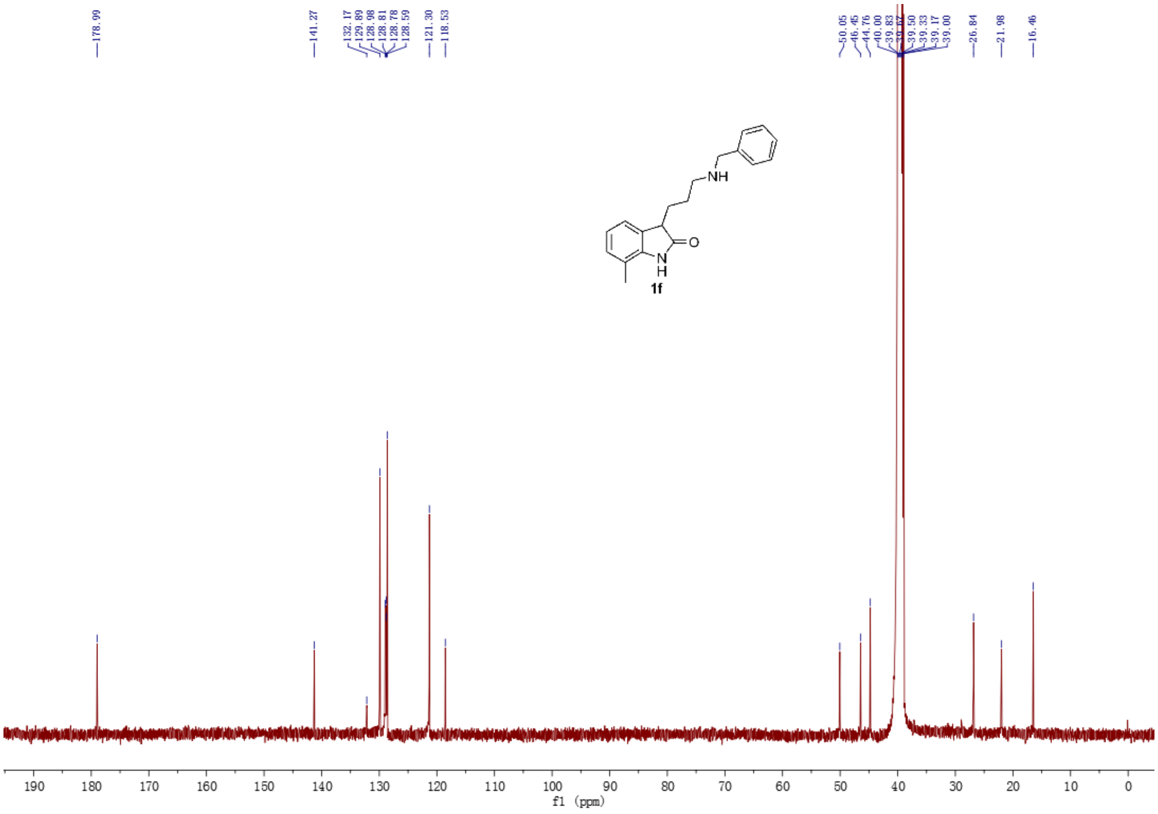
178.99
141.27
132.17
129.89
128.98
128.81
128.78
128.59
121.30
118.53
50.05
46.45
44.76
40.00
39.83
39.50
39.33
39.17
39.00
26.84
21.98
16.46
NH
O
N
H
1f
190 180 170 160 150 140 130 120 110 100 90 80 70 60 50 40 30 20 10 0
f1 (ppm)

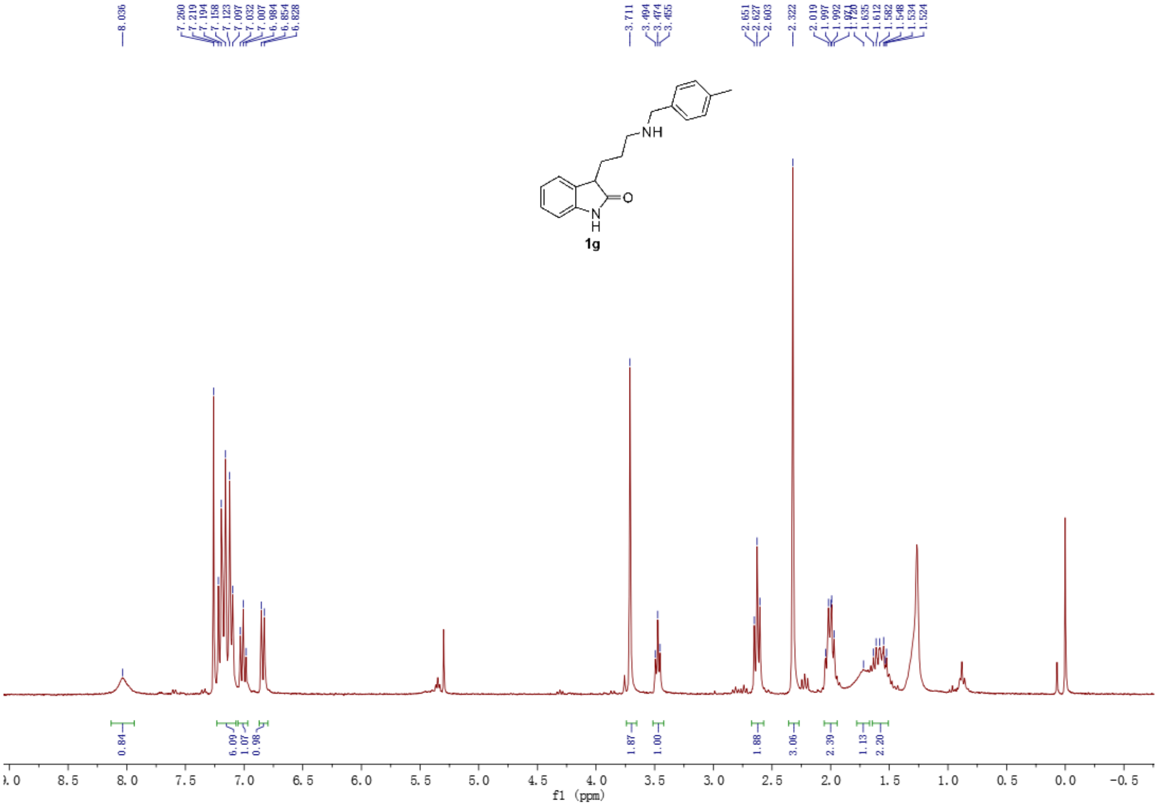
8.036
7.260
7.219
7.194
7.158
7.123
7.097
7.032
7.007
6.984
6.854
6.828
3.711
3.494
3.474
3.455
2.651
2.627
2.603
2.322
2.019
1.997
1.992
1.635
1.612
1.582
1.558
1.534
1.524
NH
O
N
H
1g
0.84
6.09
1.07
0.98
1.87
1.00
1.88
3.06
2.39
1.13
2.20
8.5 8.0 7.5 7.0 6.5 6.0 5.5 5.0 4.5 4.0 3.5 3.0 2.5 2.0 1.5 1.0 0.5 0.0 -0.5
f1 (ppm)

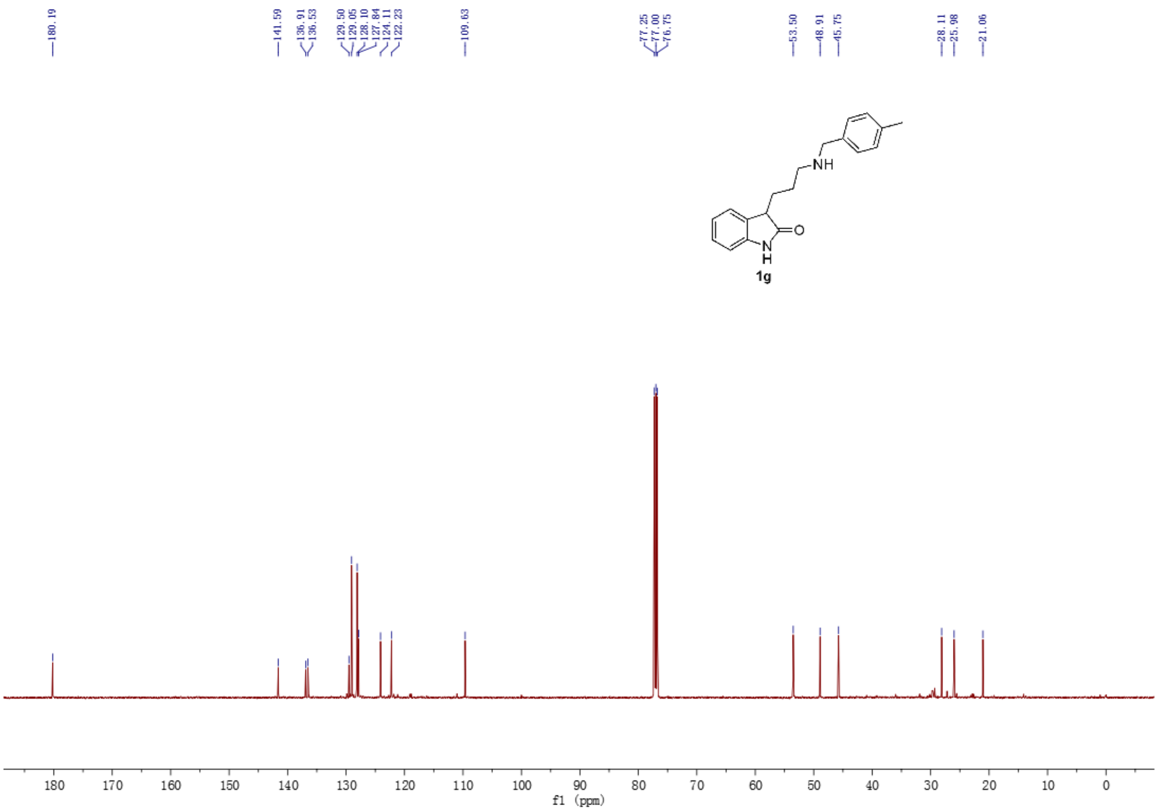

1g

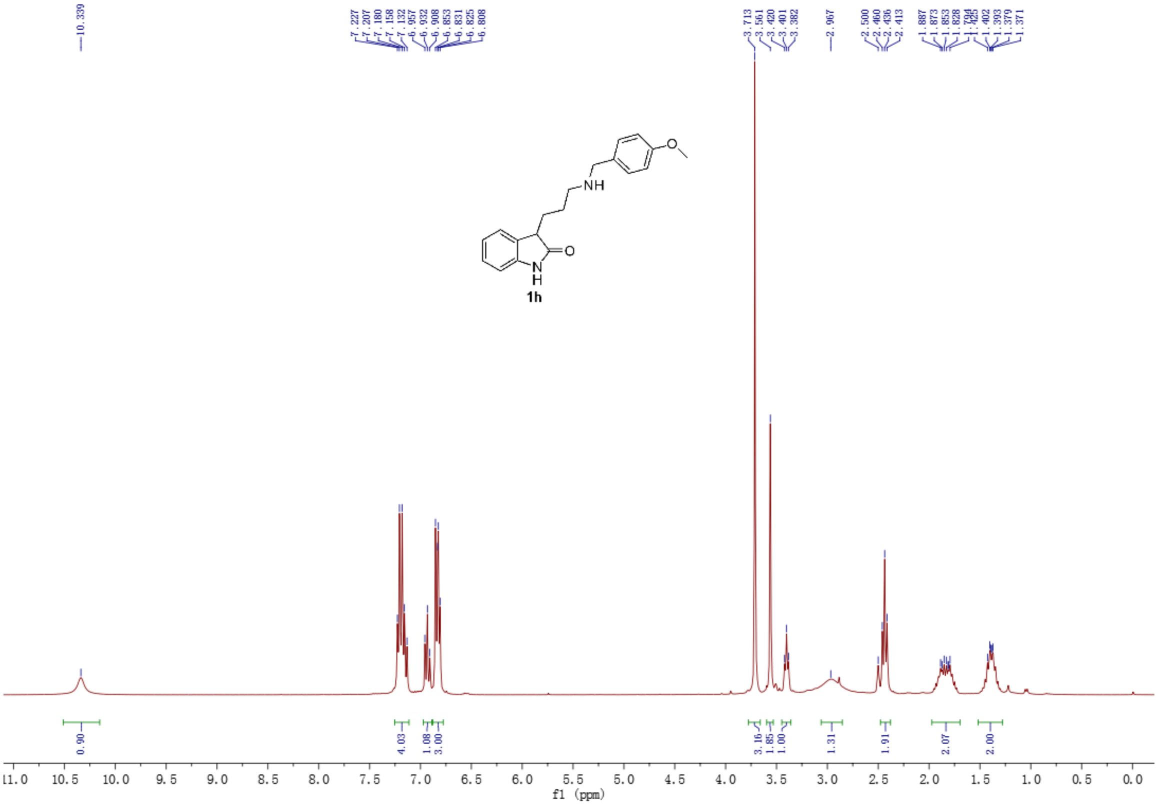

1h

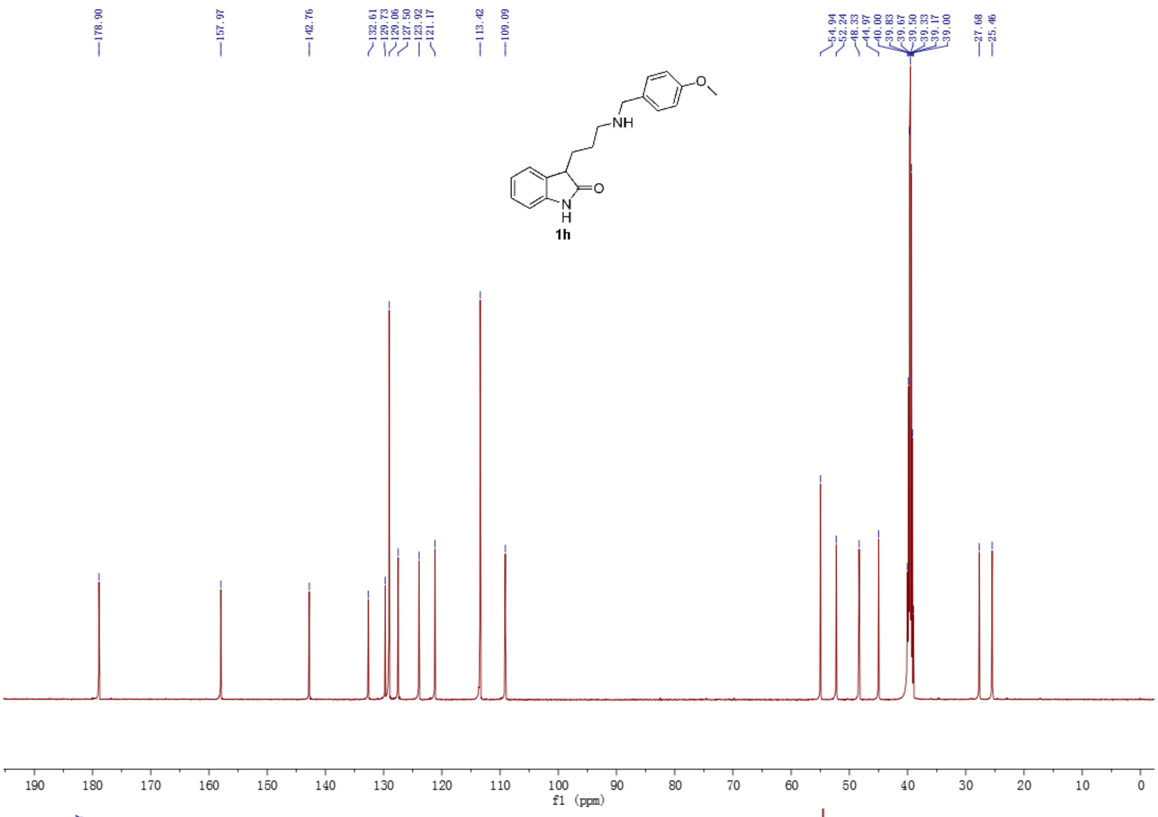

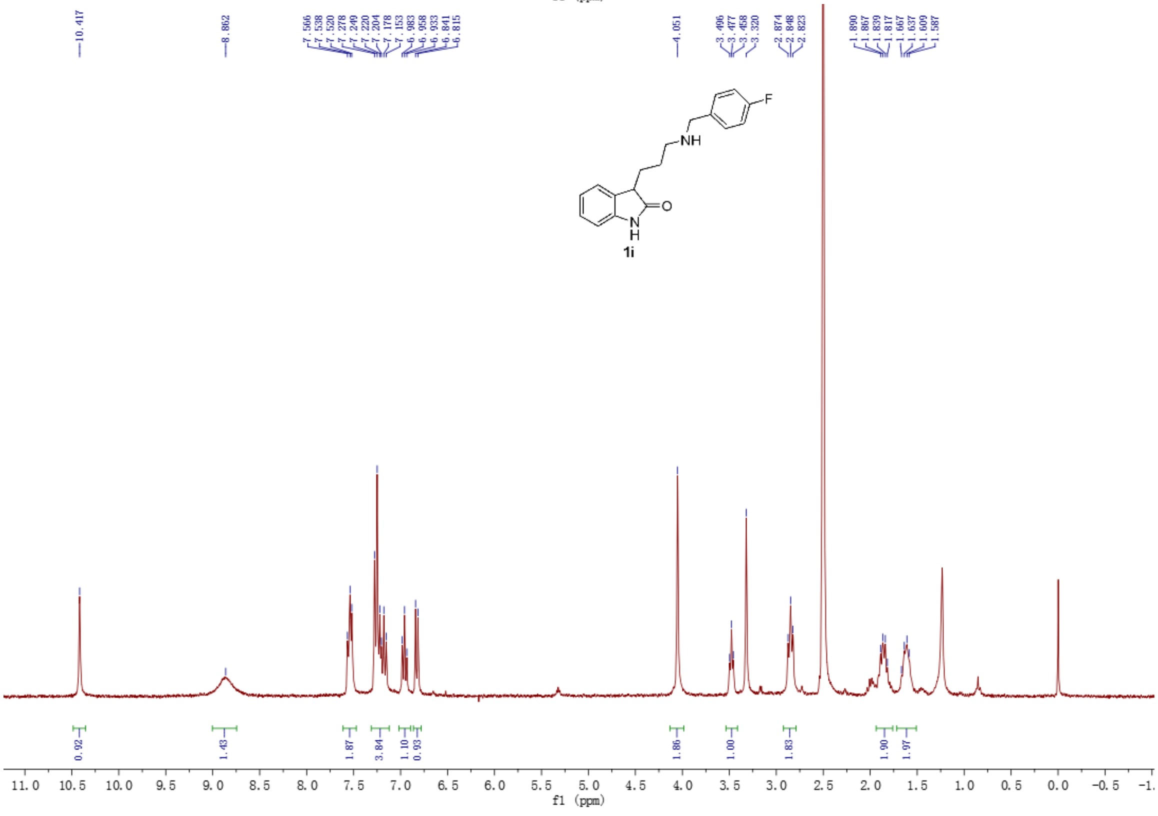

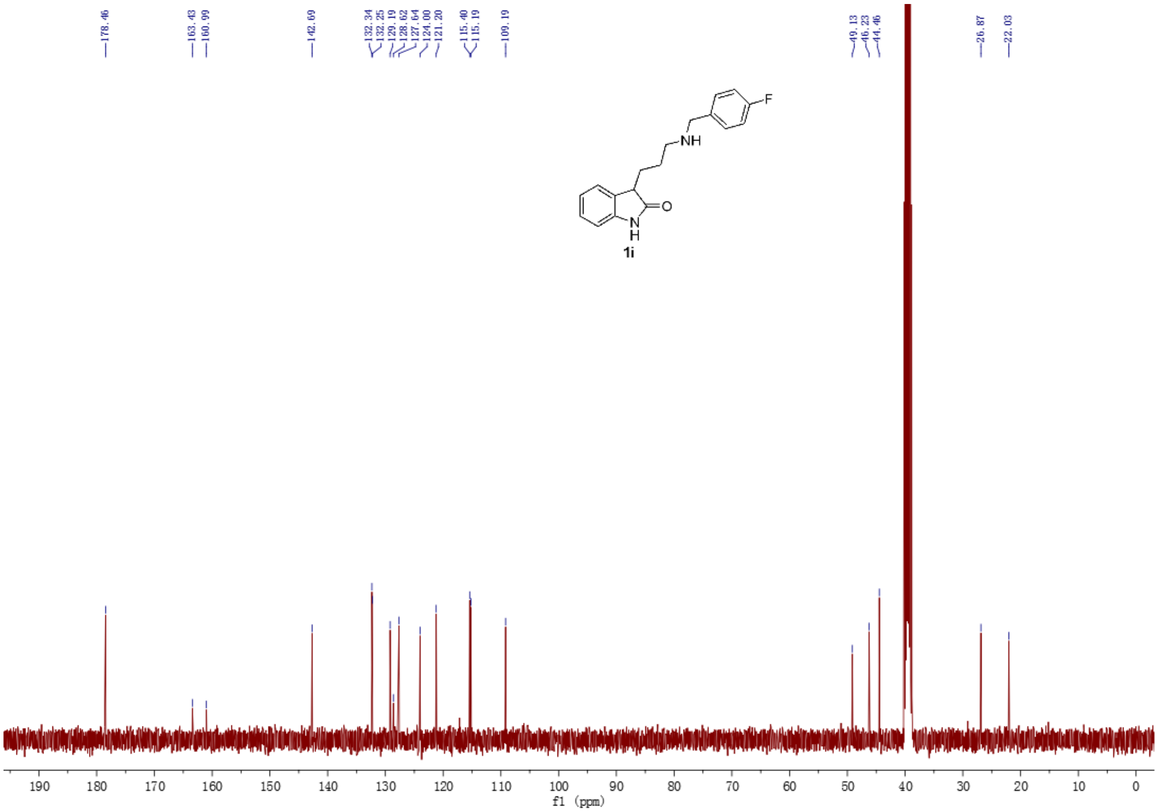
178.46
163.43
160.99
142.69
132.34
132.25
129.19
128.62
127.64
124.00
121.20
115.40
115.19
109.19
49.13
46.23
44.46
26.87
22.03
NH
F
O
N
H
1i
f1 (ppm)

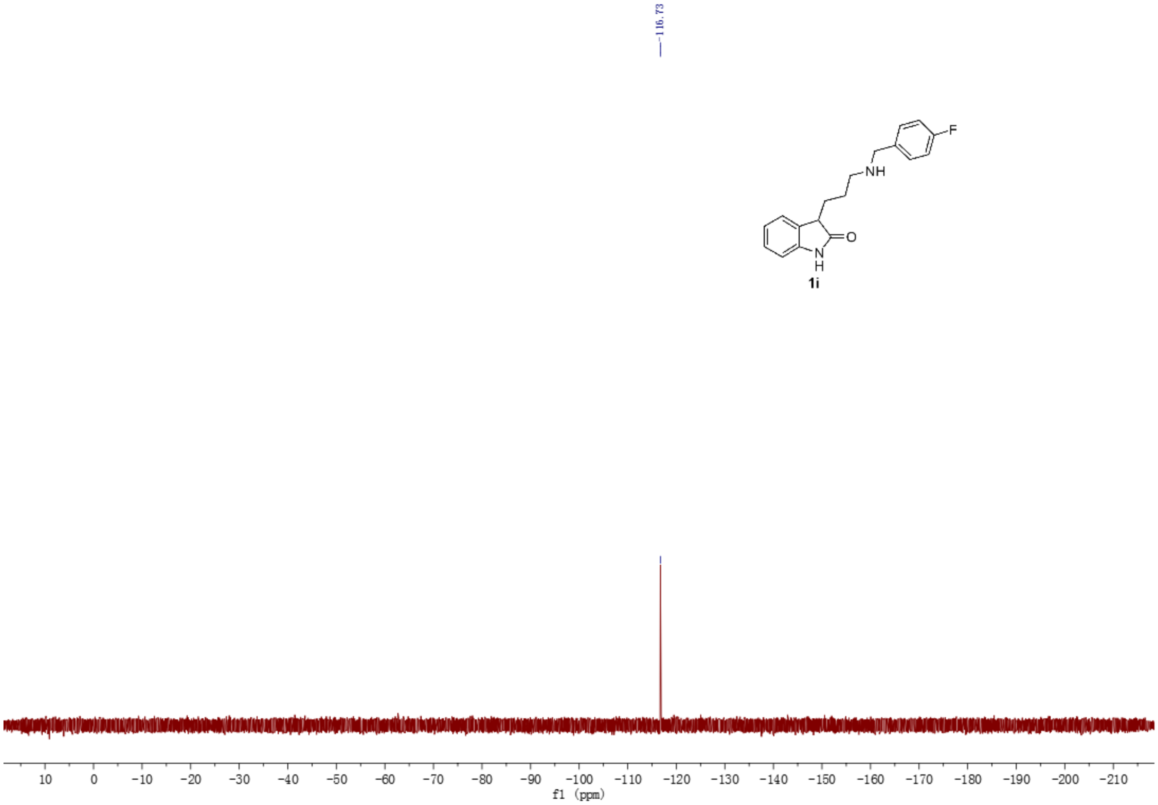
-116.73
NH
F
O
N
H
1i
f1 (ppm)

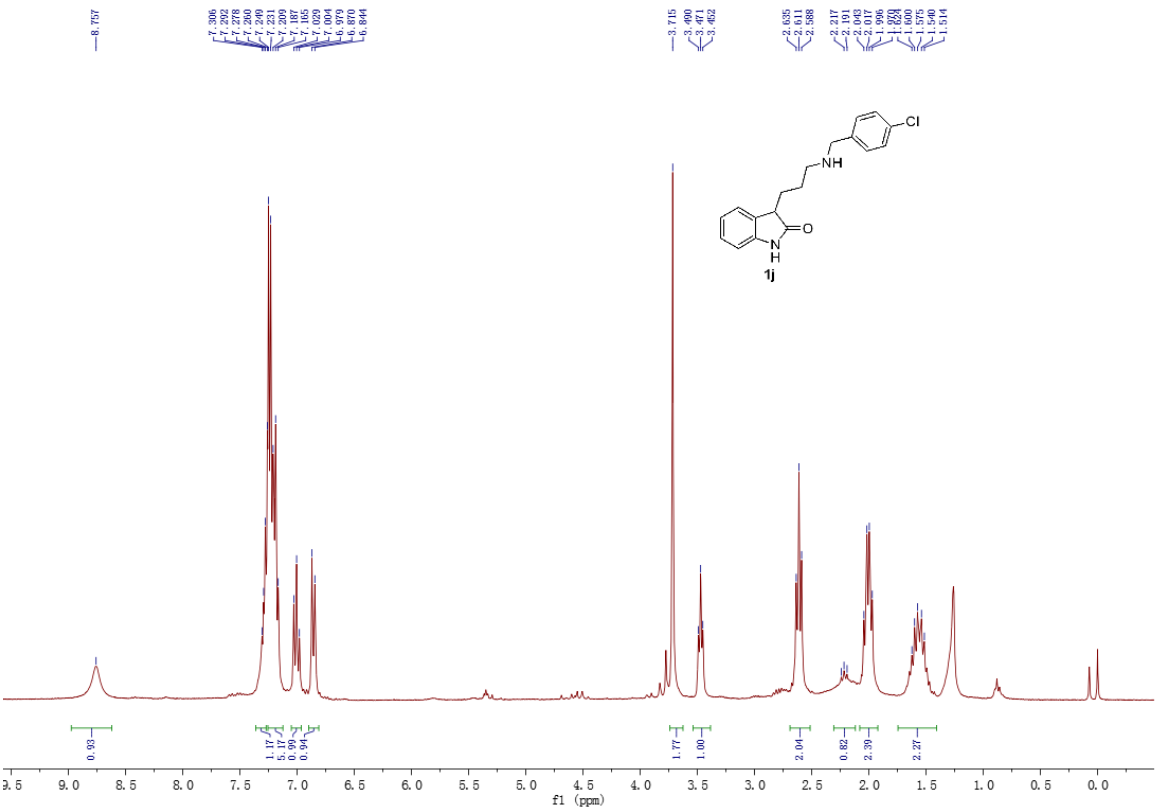
8.757
7.306
7.292
7.278
7.260
7.249
7.231
7.209
7.187
7.165
7.029
7.004
6.979
6.870
6.844
3.715
3.490
3.471
3.452
2.635
2.611
2.588
2.217
2.191
2.043
2.017
1.996
1.600
1.575
1.540
1.514
Cl
NH
O
N
H
1j
0.93
1.17
5.17
0.99
0.94
1.77
1.00
2.04
0.82
2.39
2.27
f1 (ppm)

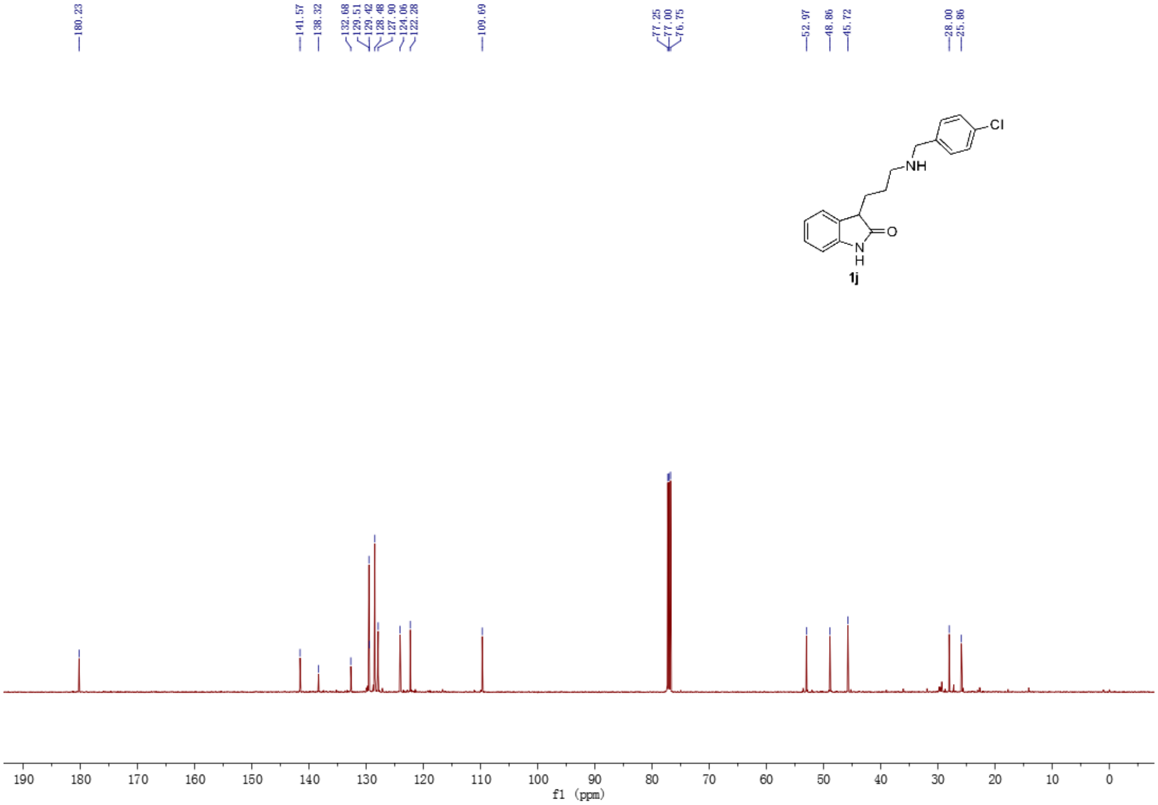
180.23
141.57
138.32
132.68
129.51
129.42
128.48
127.90
124.06
122.28
109.69
77.25
77.00
76.75
52.97
48.86
45.72
28.00
25.86
Cl
NH
O
N
H
1j
f1 (ppm)

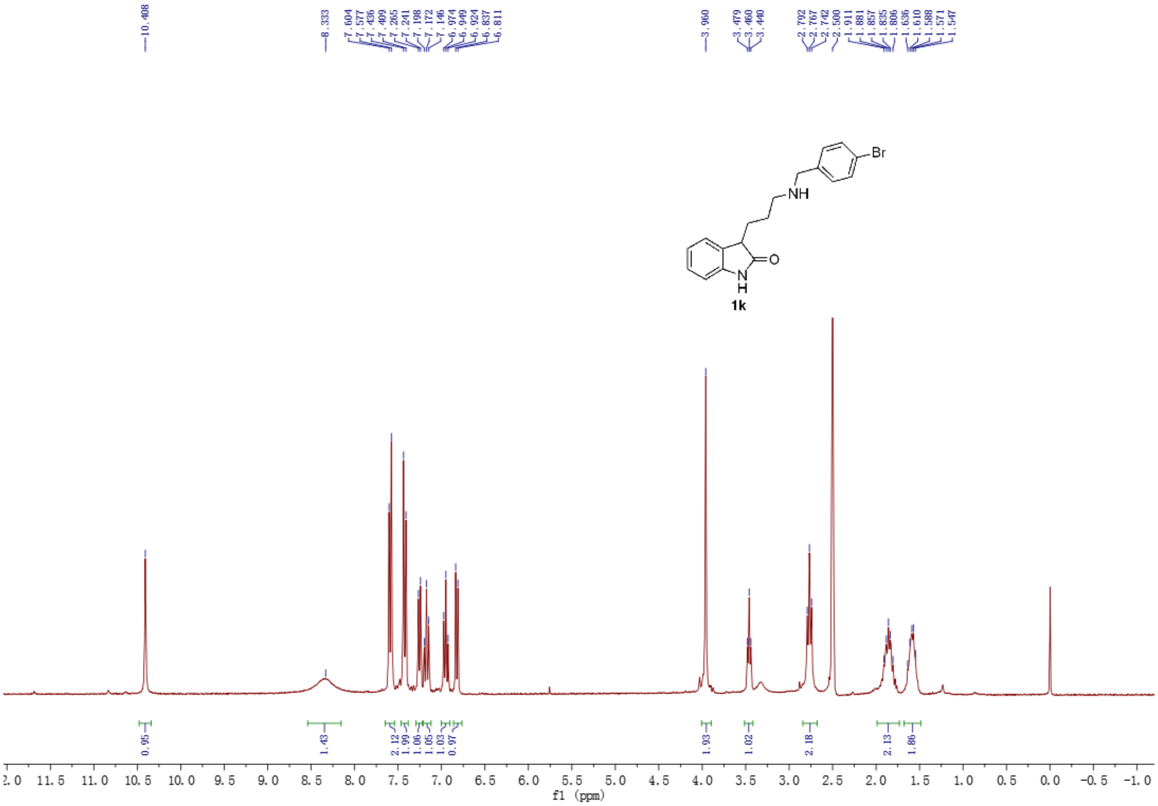
10.408
8.333
7.604
7.577
7.436
7.409
7.265
7.241
7.198
7.172
7.146
6.974
6.949
6.924
6.837
6.811
3.960
3.479
3.460
3.440
2.792
2.767
2.742
2.500
1.911
1.881
1.857
1.835
1.806
1.636
1.610
1.588
1.571
1.547
Br
NH
O
N
H
1k
0.95
1.43
2.12
1.99
1.06
1.05
1.03
0.97
1.93
1.02
2.18
2.13
1.86
f1 (ppm)

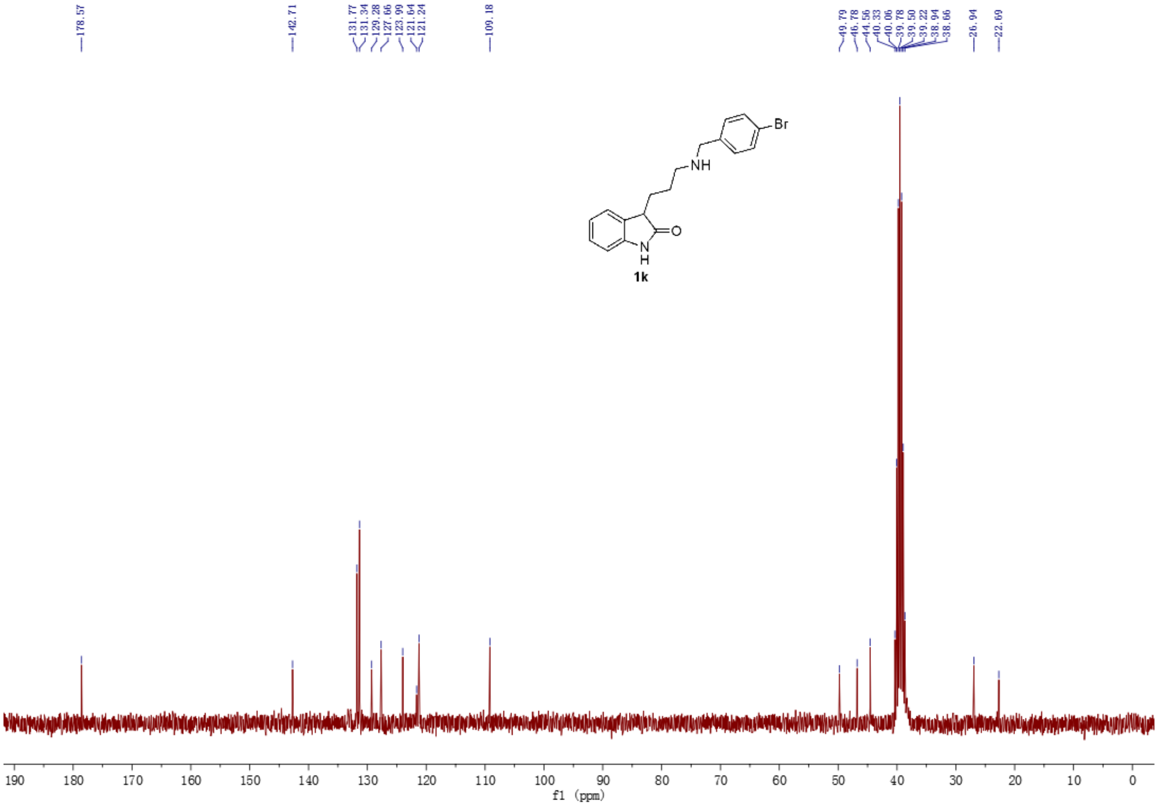
178.57
142.71
131.77
131.34
129.28
127.66
123.99
121.64
121.24
109.18
49.79
46.78
44.56
40.33
40.06
39.78
39.50
39.22
38.94
38.66
26.94
22.69
Br
NH
O
N
H
1k
f1 (ppm)

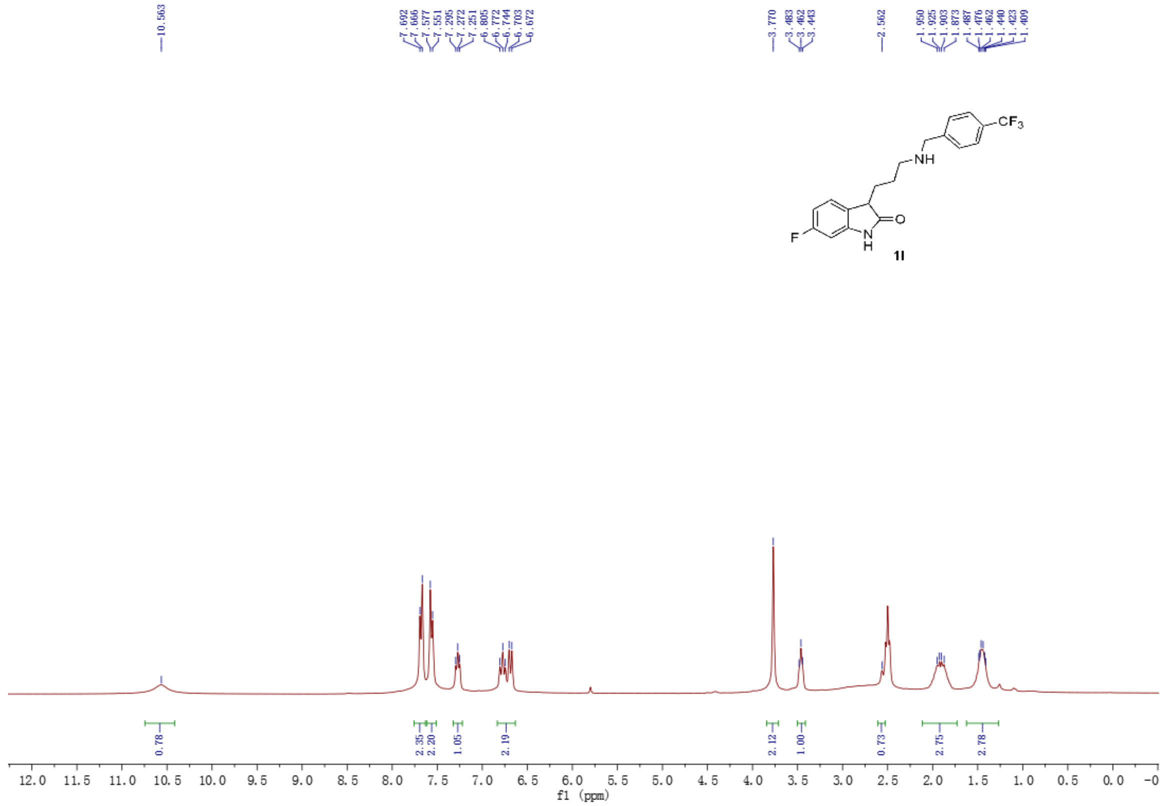
10.563
7.692 7.666 7.577 7.551 7.295 7.272 7.251 6.805 6.772 6.744 6.703 6.672
3.770 3.483 3.462 3.443
2.562
1.950 1.925 1.903 1.873 1.487 1.476 1.462 1.440 1.423 1.409
CF3
NH
F
N
H
O
1l
0.78
2.35 2.20
1.05
2.19
2.12
1.00
0.73
2.75
2.78
f1 (ppm)

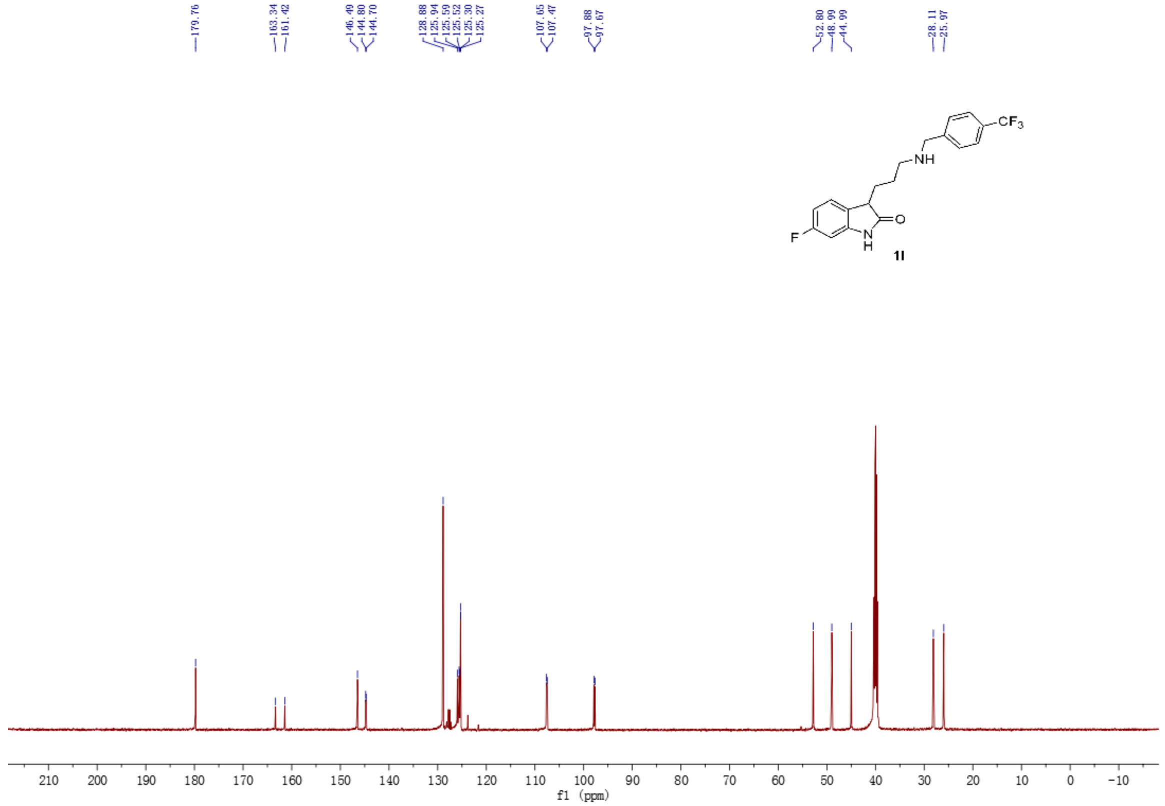
179.76
163.34 161.42
146.49 144.80 144.70
128.88 125.94 125.59 125.52 125.30 125.27
107.65 107.47
97.88 97.67
52.80 48.99 44.99
28.11 25.97
CF3
NH
F
N
H
O
1l
f1 (ppm)

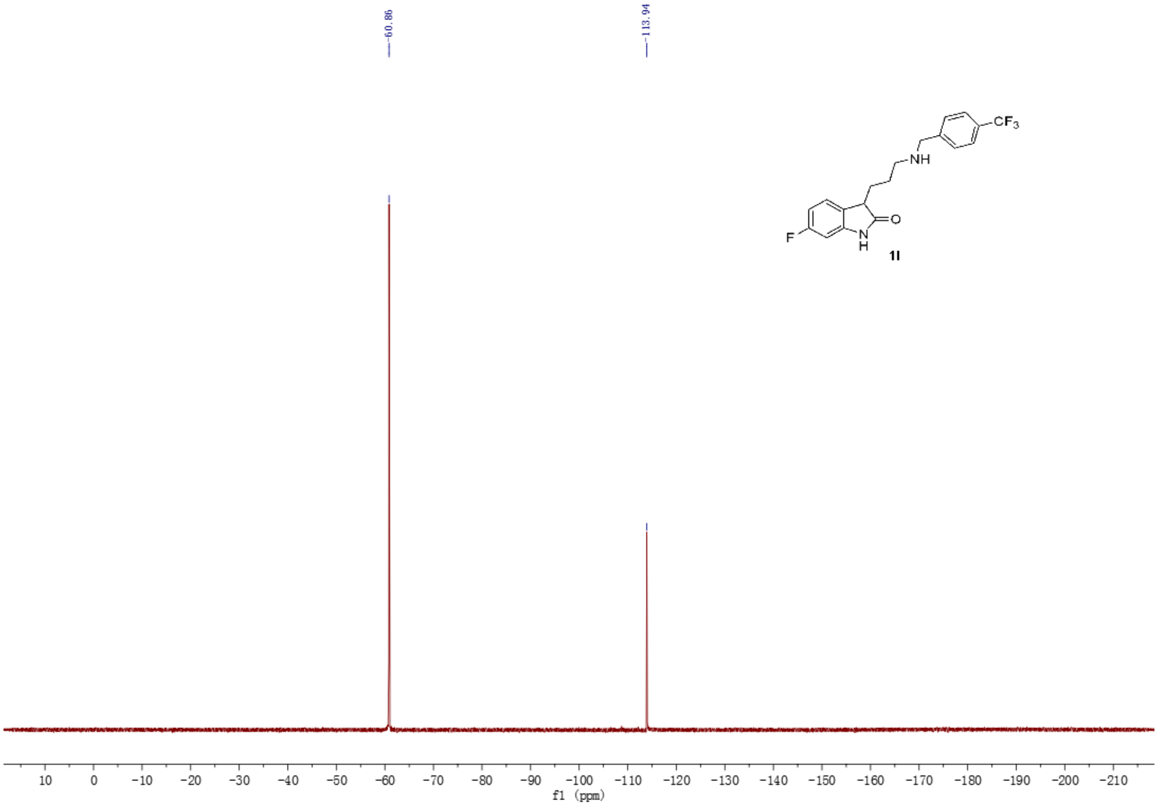
-60.86
-113.94
CF3
NH
O
F
H
1l
f1 (ppm)

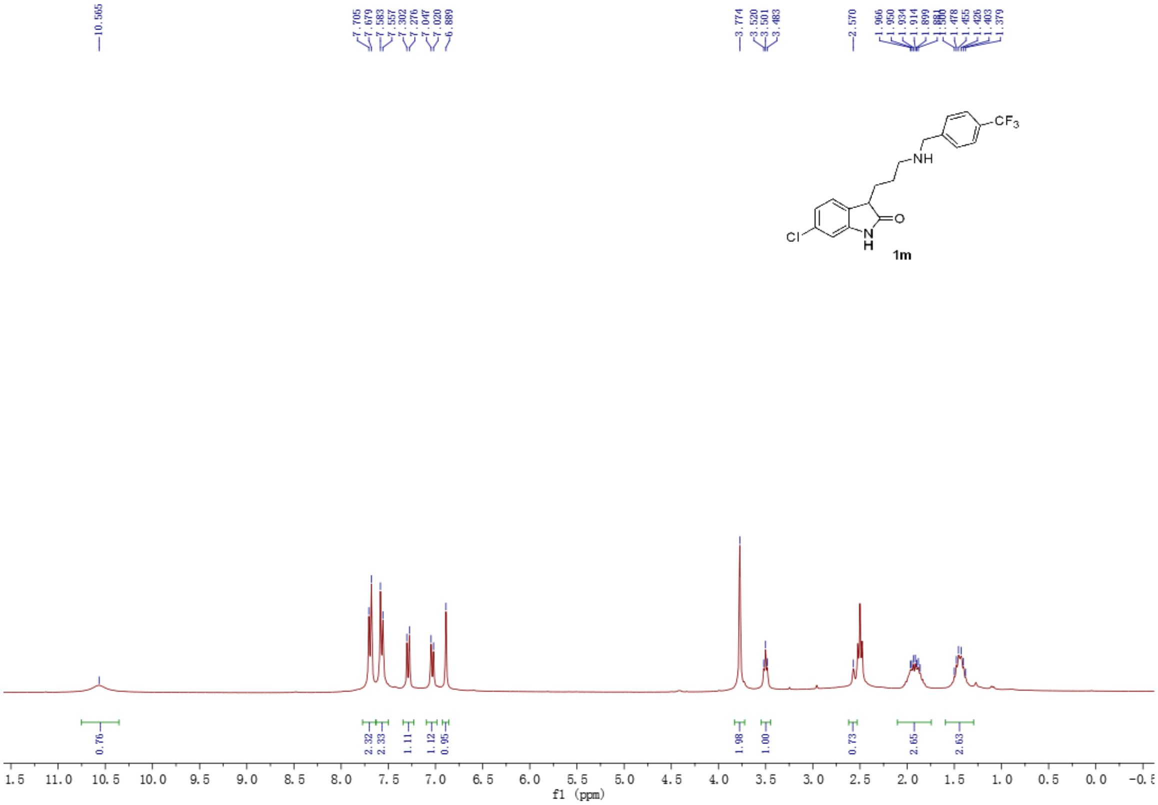
CF3
NH
O
Cl
H
1m
f1 (ppm)

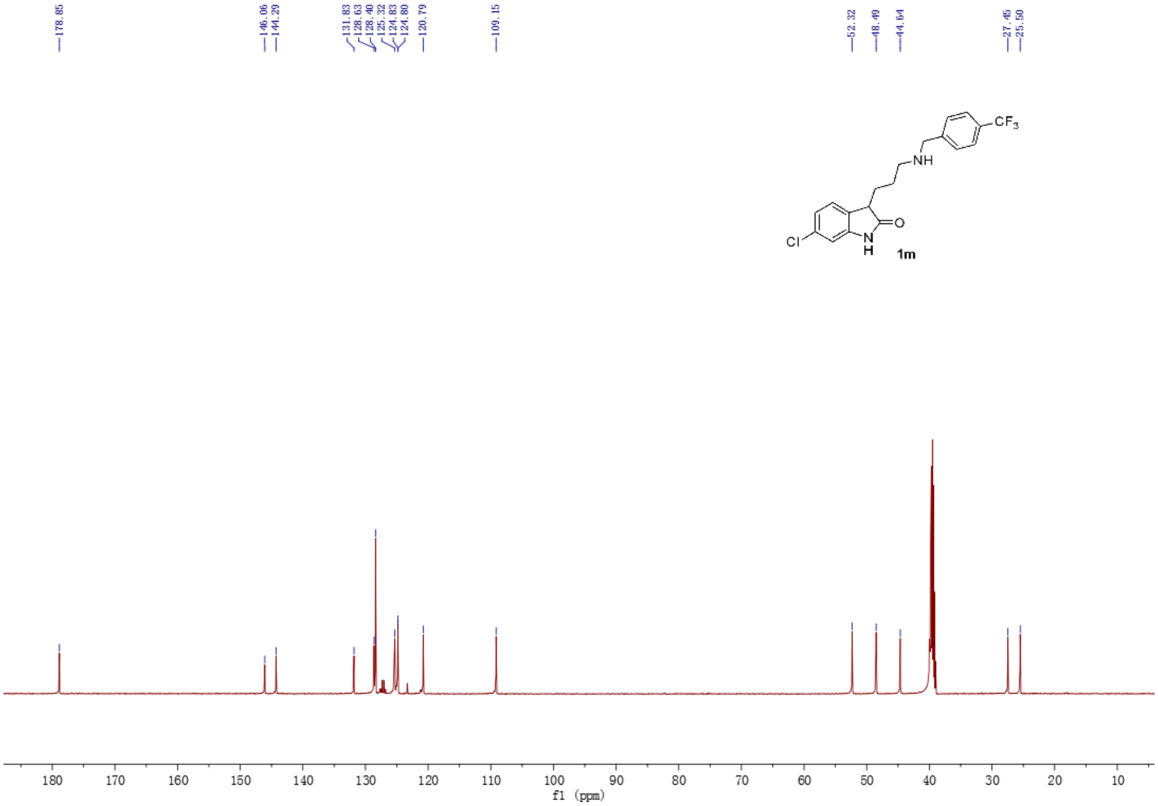
178.85
146.06
144.29
131.83
128.63
128.40
125.32
124.83
124.80
120.79
109.15
52.32
48.49
44.64
27.45
25.50
CF3
NH
Cl
N
H
O
1m
180
170
160
150
140
130
120
110
100
90
80
70
60
50
40
30
20
10
f1 (ppm)

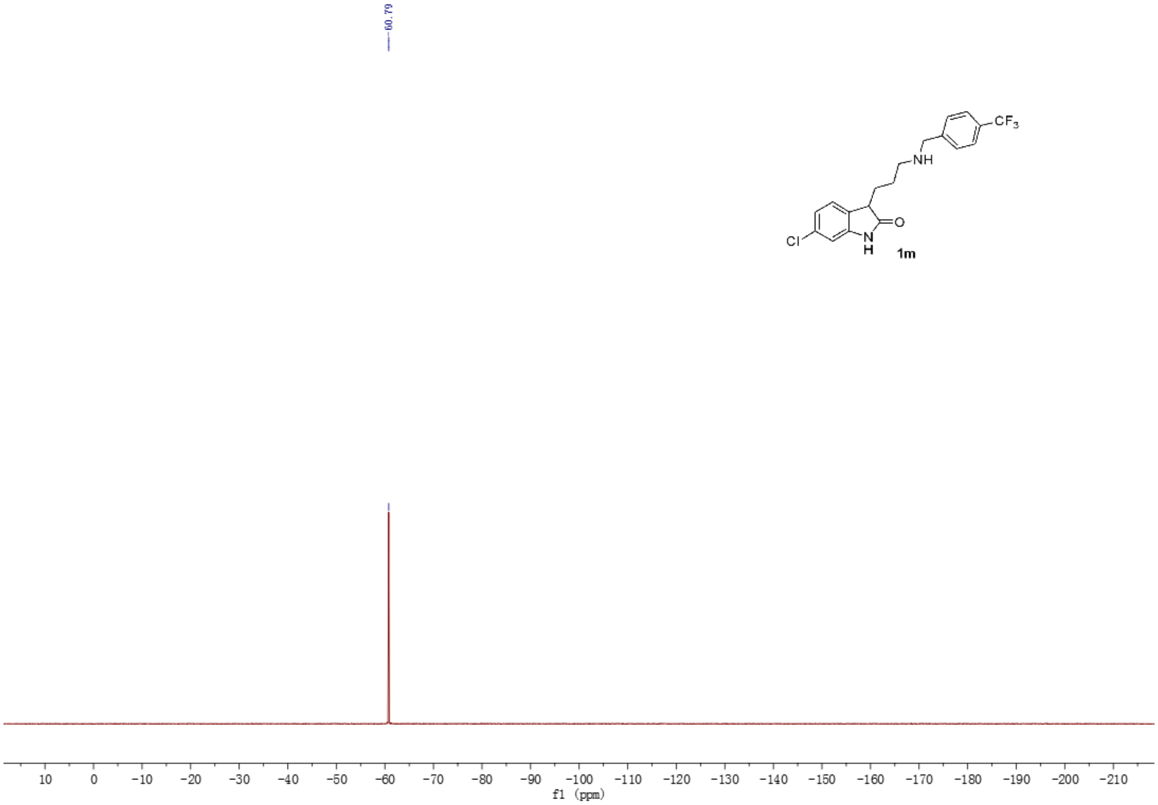
-60.79
CF3
NH
Cl
N
H
O
1m
10
0
-10
-20
-30
-40
-50
-60
-70
-80
-90
-100
-110
-120
-130
-140
-150
-160
-170
-180
-190
-200
-210
f1 (ppm)

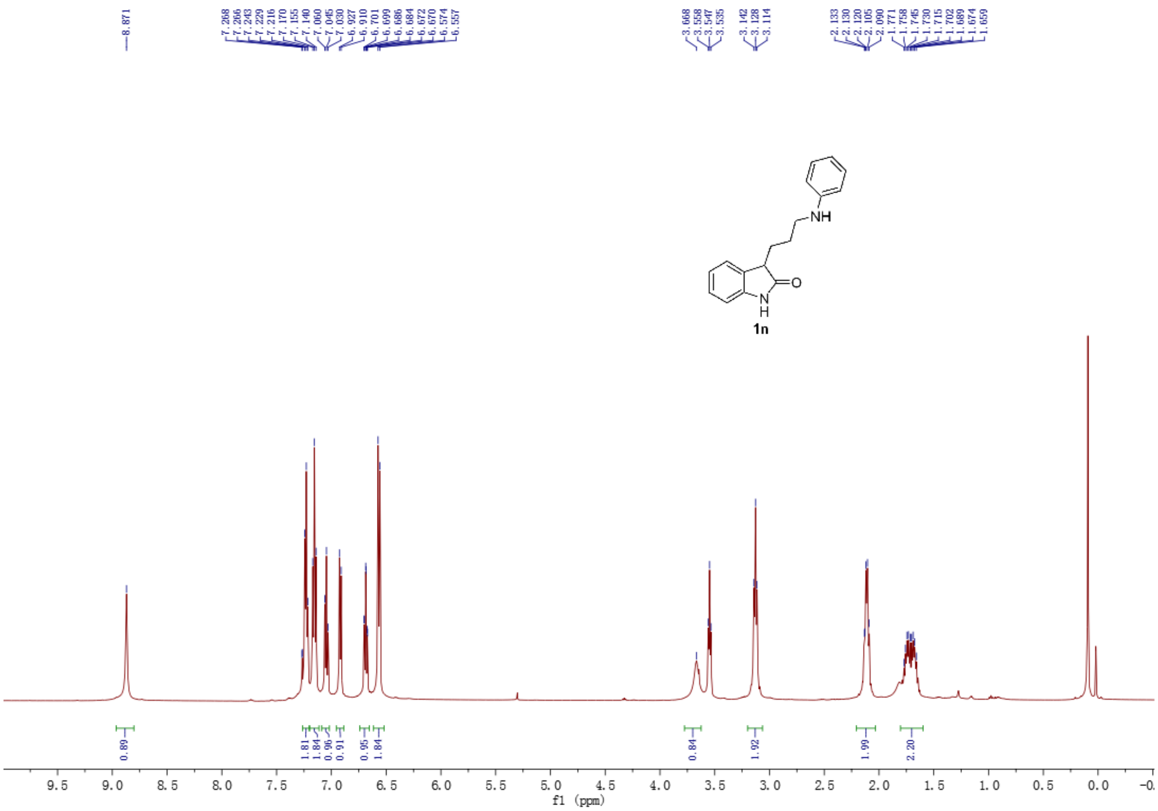
NH
O
N
H
1n
f1 (ppm)

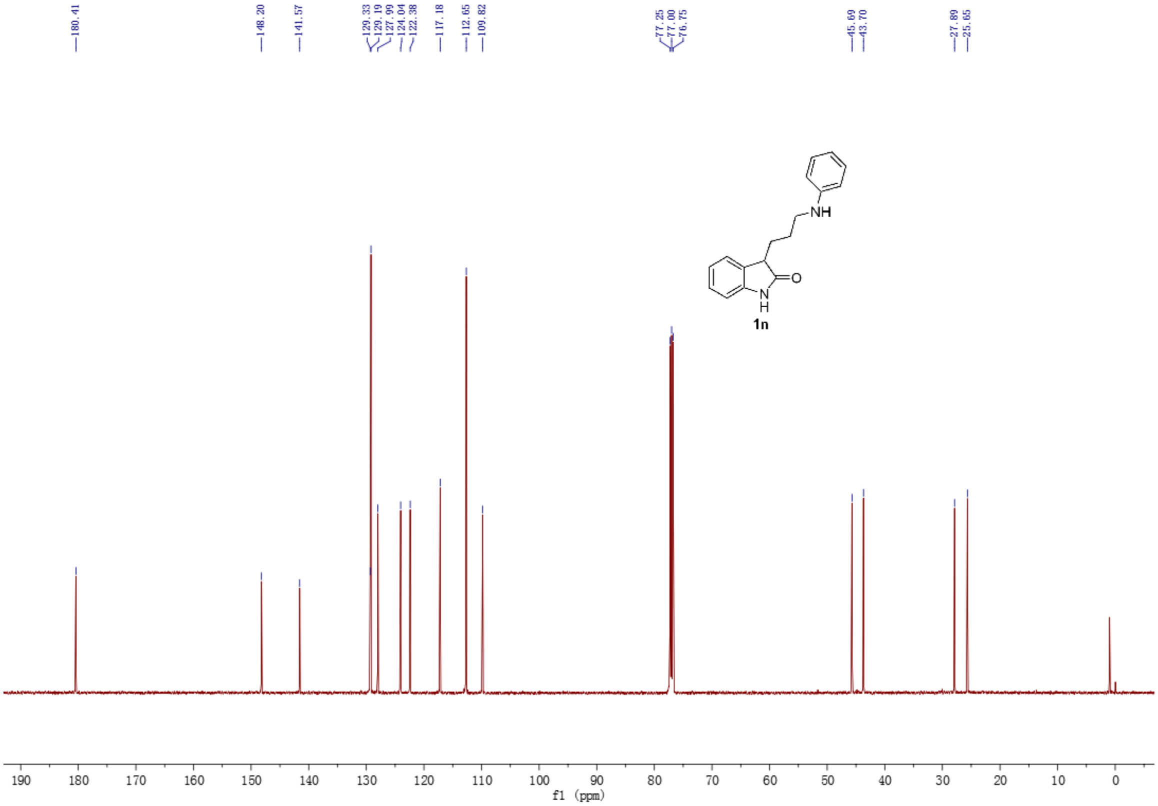
180.41
148.20
141.57
129.33
129.19
127.99
124.04
122.38
117.18
112.65
109.82
77.25
77.00
76.75
45.69
43.70
27.89
25.65
NH
O
N
H
1n
f1 (ppm)

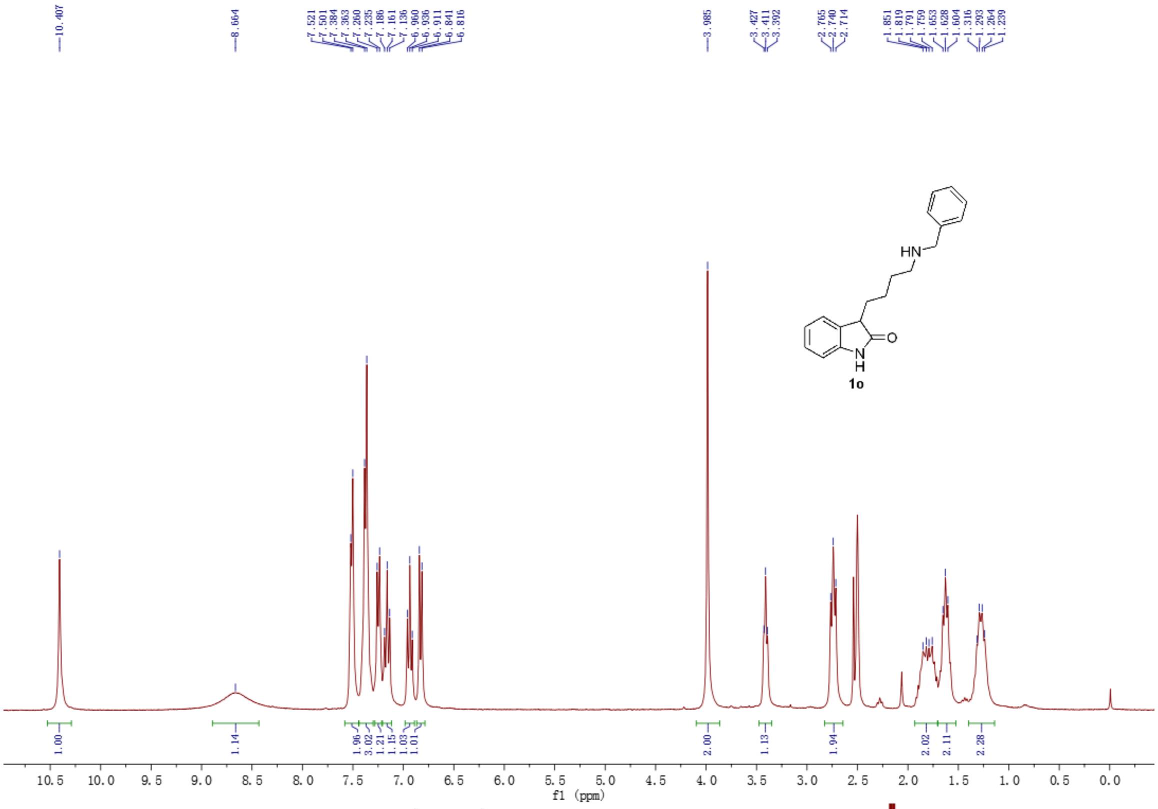
HN
O
N
H
1o
f1 (ppm)

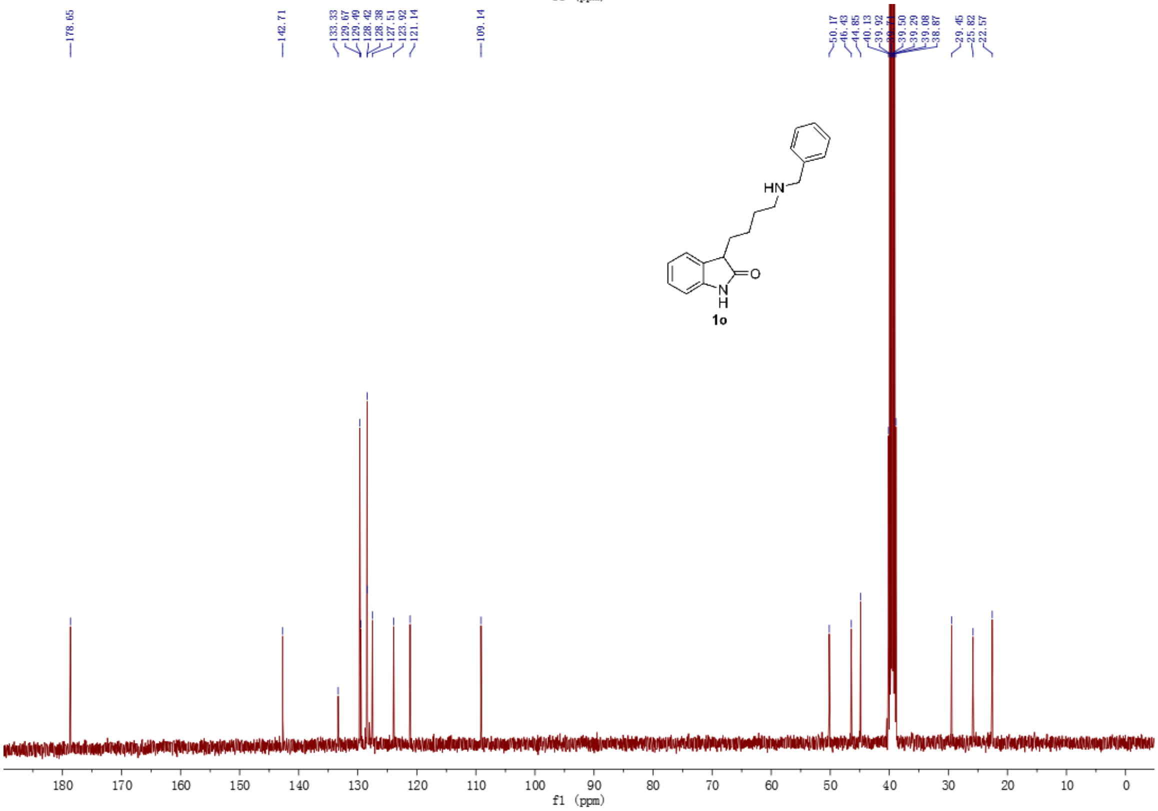
HN
O
N
H
1o
f1 (ppm)

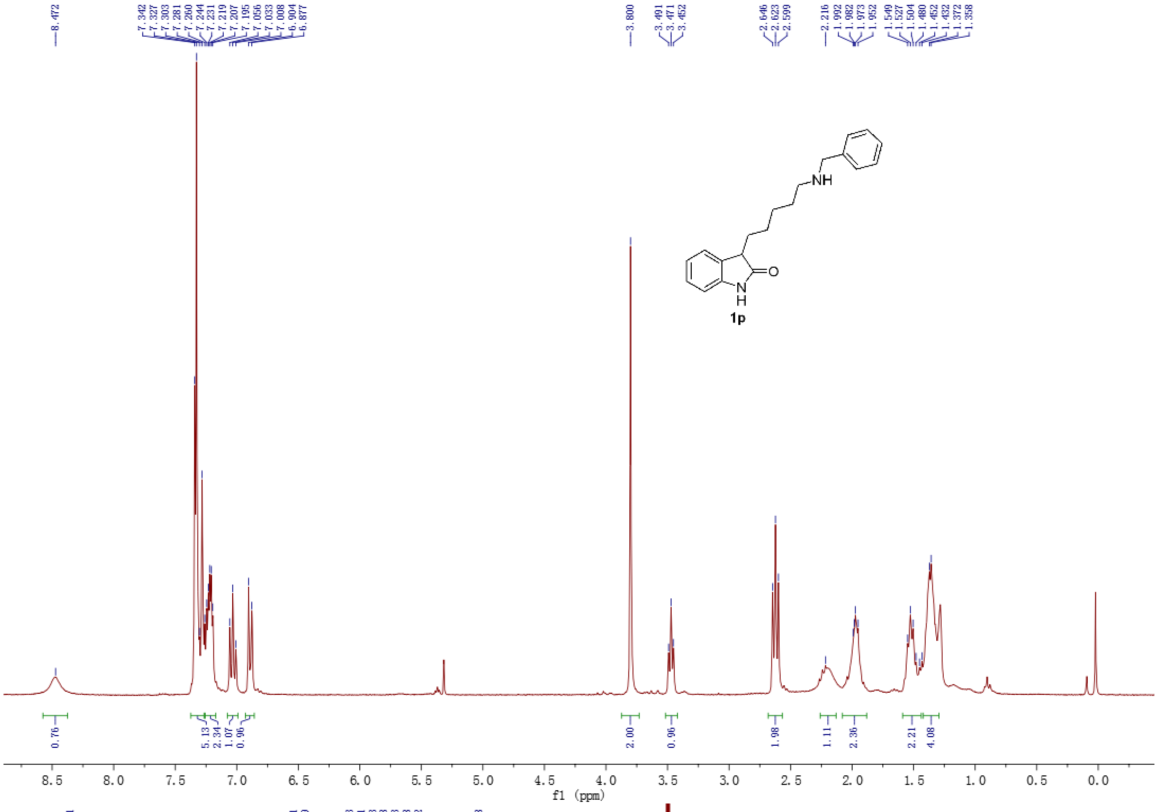
8.472
7.342 7.327 7.303 7.281 7.260 7.244 7.231 7.219 7.207 7.195 7.056 7.033 7.008 6.904 6.877
3.800
3.491 3.471 3.452
2.646 2.623 2.599
2.216 1.992 1.982 1.973 1.952
1.549 1.527 1.504 1.480 1.452 1.432 1.372 1.358
NH
O
N
H
1p
0.76 5.13 2.34 1.07 0.96 2.00 0.96 1.98 1.11 2.36 2.21 4.08
f1 (ppm)

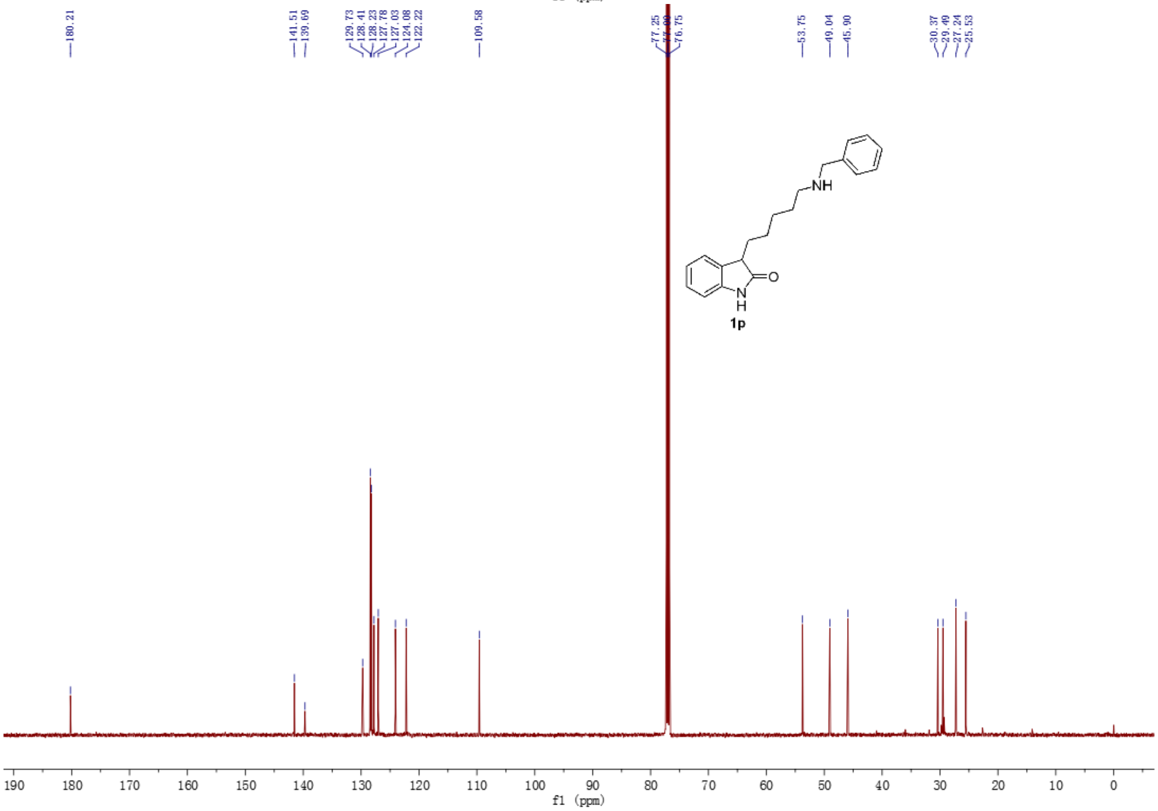
180.21
141.51 139.69
129.73 128.41 128.23 127.78 127.03 124.08 122.22
109.58
77.25 76.75
53.75 49.04 45.90
30.37 29.49 27.24 25.53
NH
O
N
H
1p
f1 (ppm)

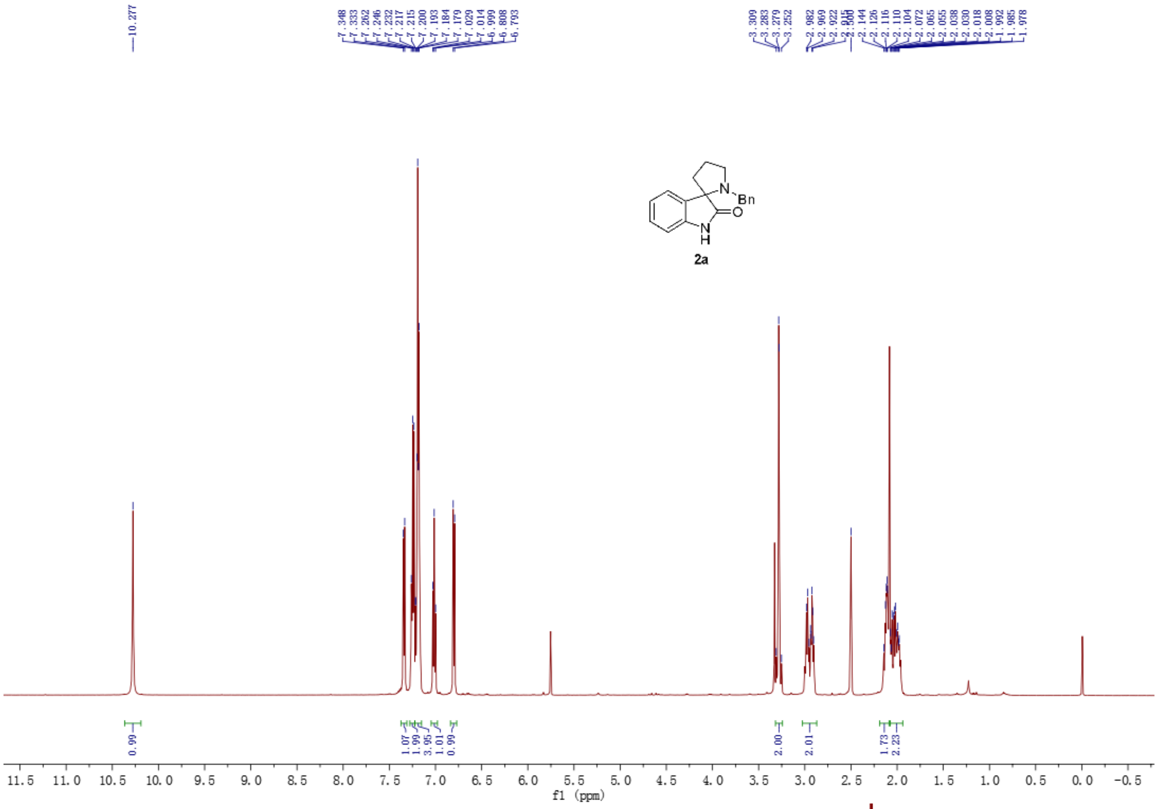
10.277
7.348
7.333
7.262
7.246
7.232
7.217
7.215
7.200
7.193
7.184
7.179
7.029
7.014
6.999
6.808
6.793
3.283
3.279
3.252
2.982
2.969
2.922
2.144
2.126
2.116
2.110
2.104
2.072
2.065
2.055
2.038
2.030
2.018
2.008
1.992
1.985
1.978
N
Bn
O
H
2a
0.99
1.07
1.99
3.95
1.01
0.99
2.00
2.01
1.73
2.23
11.5 11.0 10.5 10.0 9.5 9.0 8.5 8.0 7.5 7.0 6.5 6.0 5.5 5.0 4.5 4.0 3.5 3.0 2.5 2.0 1.5 1.0 0.5 0.0 -0.5
f1 (ppm)

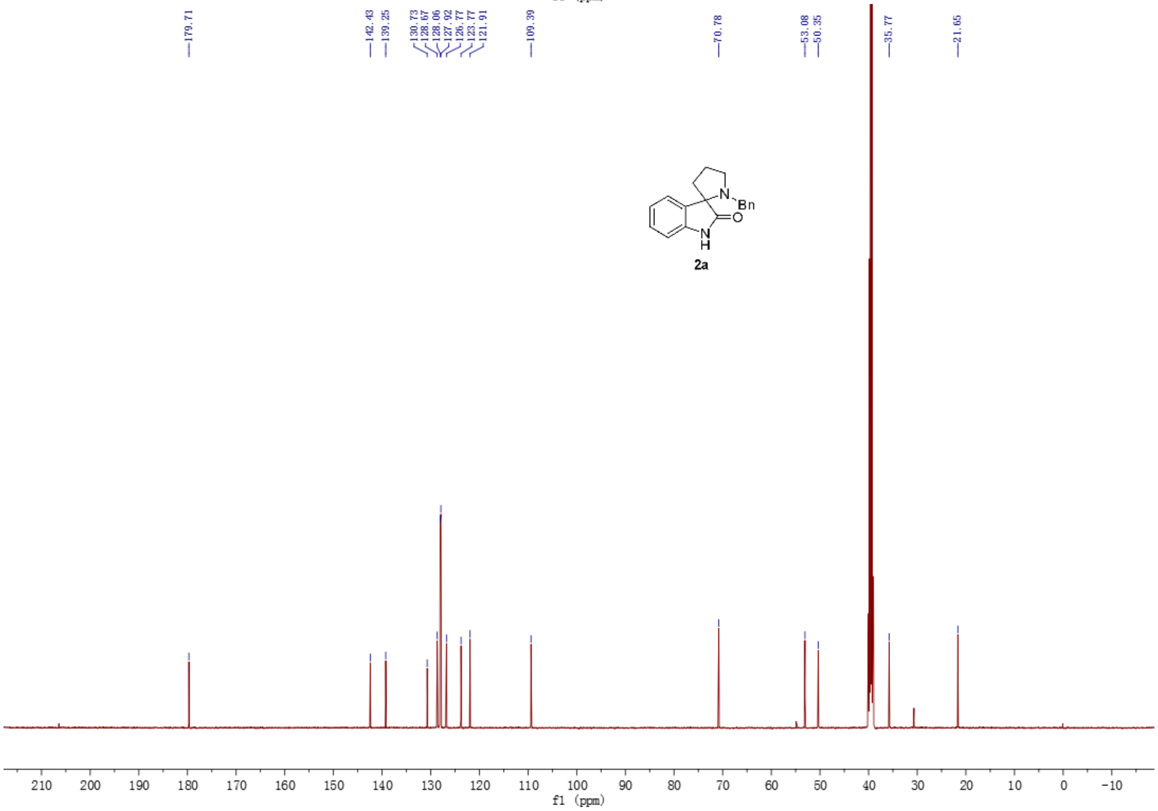
179.71
142.43
139.25
130.73
128.67
128.06
127.92
126.77
123.77
121.91
109.39
70.78
53.08
50.35
35.77
21.65
N
Bn
O
H
2a
210 200 190 180 170 160 150 140 130 120 110 100 90 80 70 60 50 40 30 20 10 0 -10
f1 (ppm)

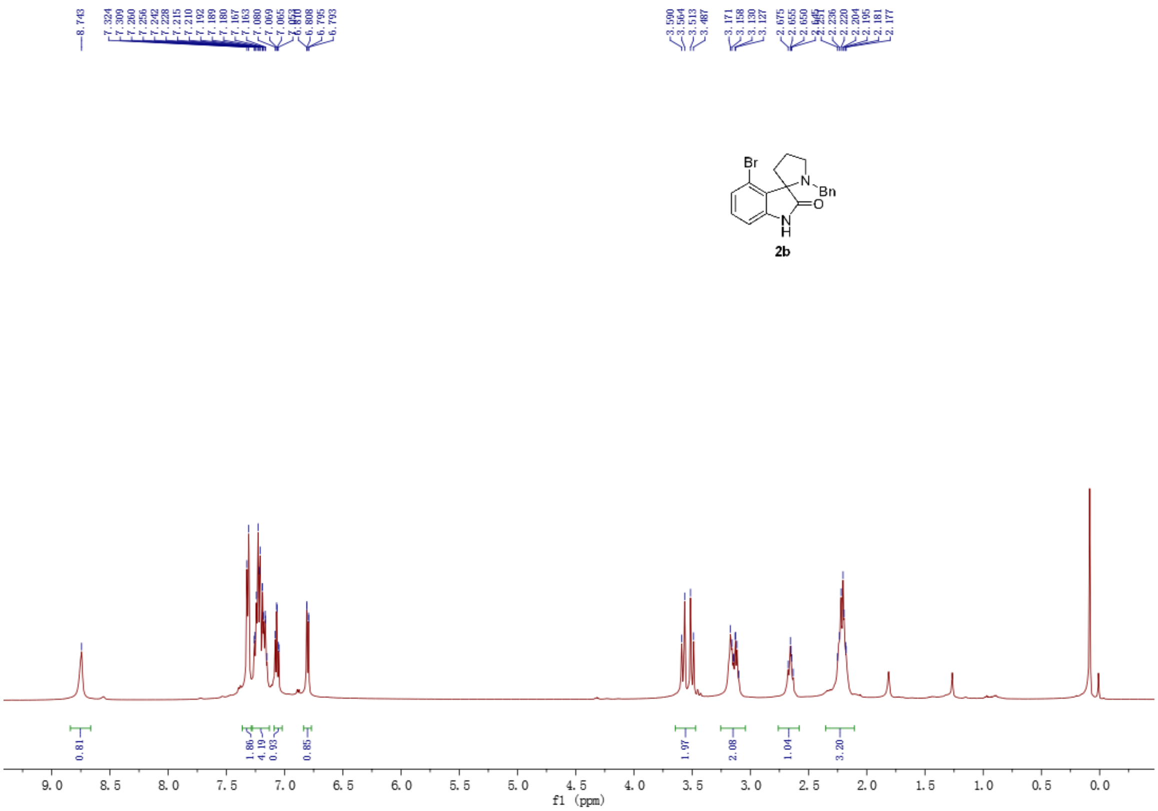
8.743
7.324 7.309 7.260 7.256 7.242 7.228 7.215 7.210 7.192 7.189 7.180 7.167 7.163 7.080 7.069 7.065
6.808 6.795 6.793
3.590 3.564 3.513 3.487
3.171 3.158 3.130 3.127
2.675 2.655 2.650
2.236 2.220 2.204 2.195 2.181 2.177
Br
N
Bn
O
H
2b
0.81
1.86
4.19
0.93
0.85
1.97
2.08
1.04
3.20
9.0 8.5 8.0 7.5 7.0 6.5 6.0 5.5 5.0 4.5 4.0 3.5 3.0 2.5 2.0 1.5 1.0 0.5 0.0
f1 (ppm)

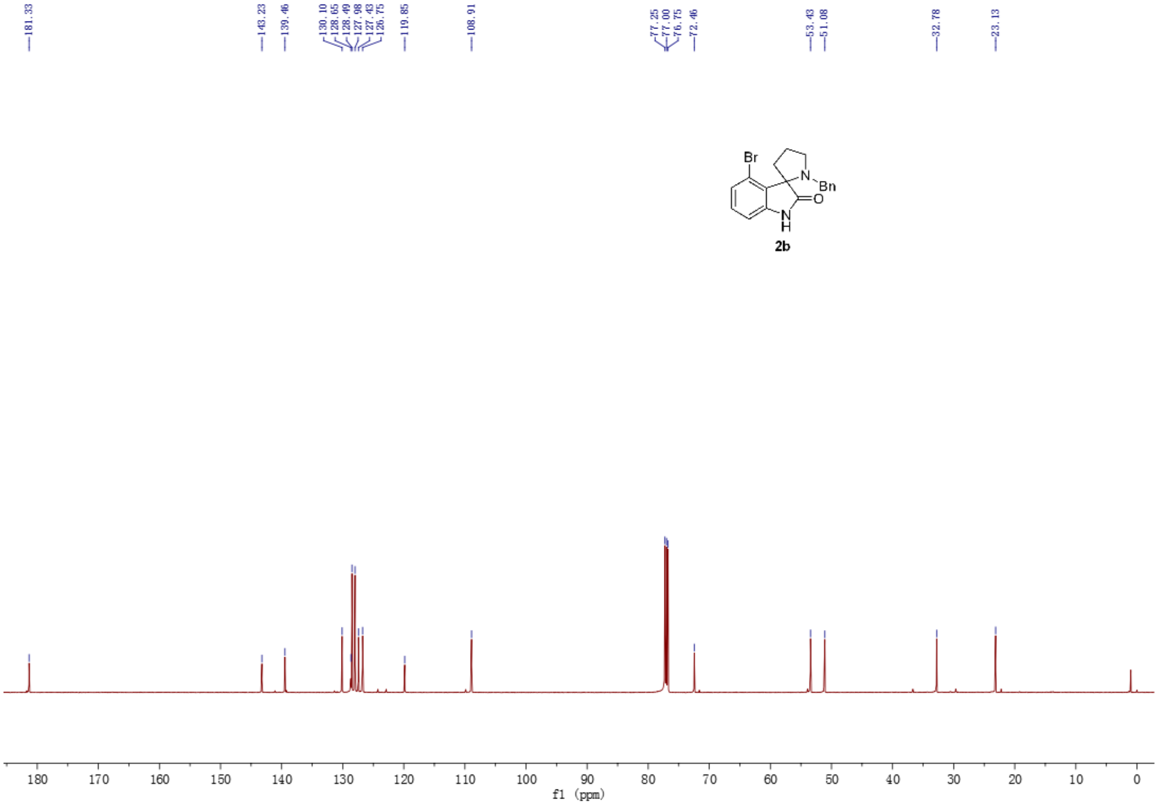
181.33
143.23
139.46
130.10 128.65 128.49 127.98 127.43 126.75
119.85
108.91
77.25 77.00 76.75
72.46
53.43
51.08
32.78
23.13
Br
N
Bn
O
H
2b
180 170 160 150 140 130 120 110 100 90 80 70 60 50 40 30 20 10 0
f1 (ppm)

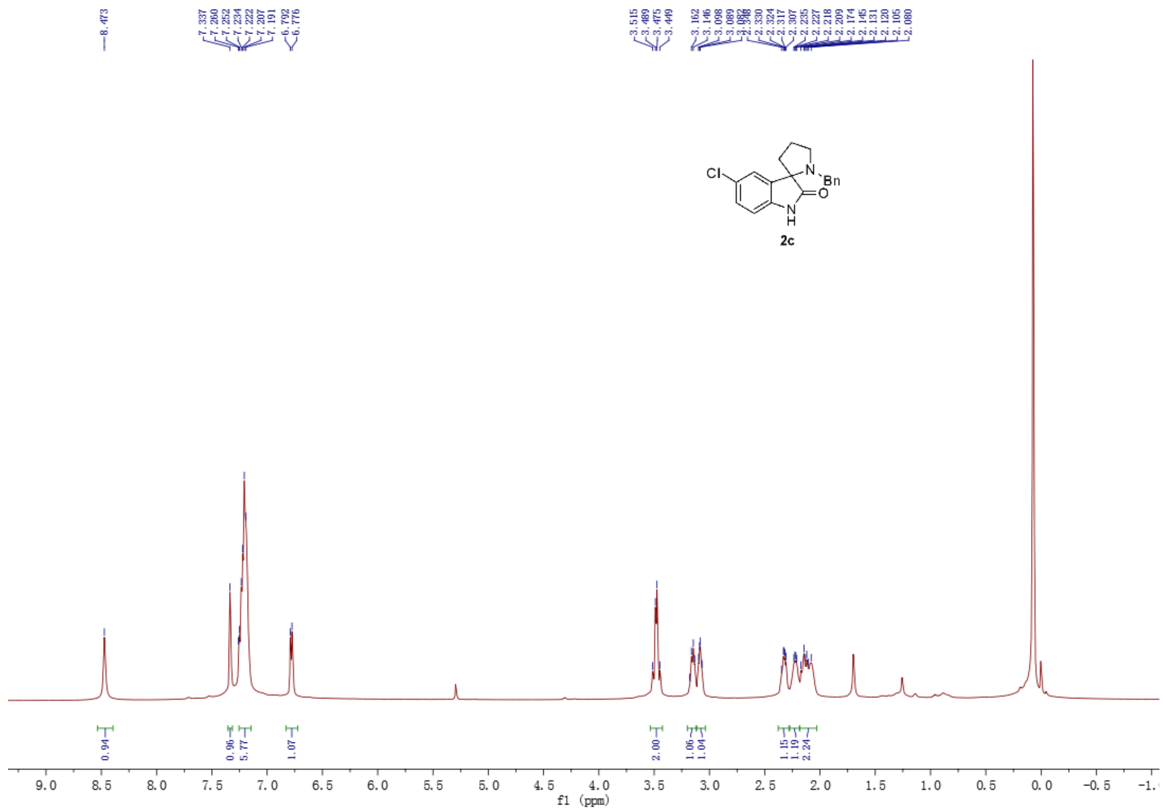
8.473
7.337
7.260
7.252
7.234
7.222
7.207
7.191
6.792
6.776
3.515
3.489
3.475
3.449
3.162
3.146
3.098
3.089
2.330
2.324
2.317
2.307
2.235
2.227
2.218
2.209
2.174
2.145
2.131
2.120
2.105
2.080
Cl
N
Bn
O
H
2c
0.94
0.96
5.77
1.07
2.00
1.06
1.04
1.15
1.19
2.24
f1 (ppm)

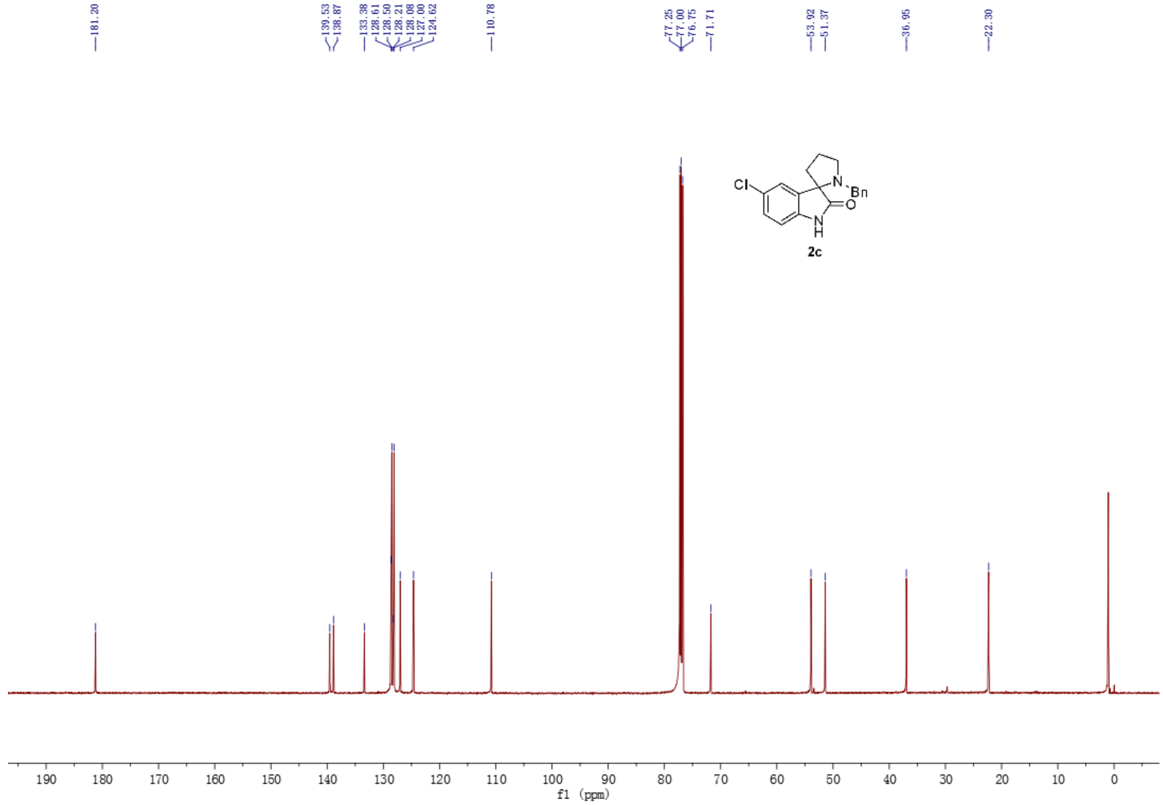
181.20
139.53
138.87
133.38
128.61
128.50
128.21
128.08
127.00
124.62
110.78
77.25
77.00
76.75
71.71
53.92
51.37
36.95
22.30
Cl
N
Bn
O
H
2c
f1 (ppm)

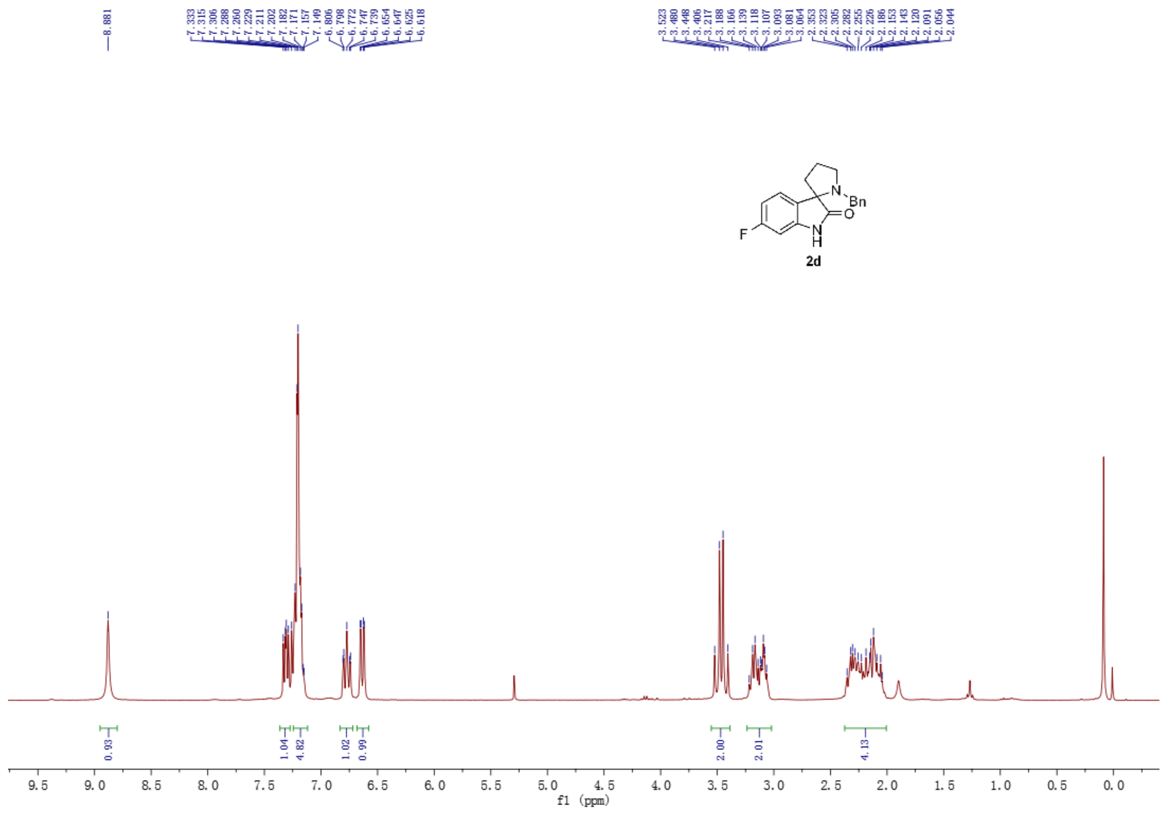
8.881
7.333 7.315 7.306 7.288 7.260 7.229 7.211 7.202 7.182 7.171 7.157 7.149 6.806 6.798 6.772 6.747 6.739 6.654 6.647 6.625 6.618
3.523 3.480 3.448 3.406 3.217 3.188 3.166 3.139 3.118 3.107 3.093 3.081 3.064 2.353 2.323 2.305 2.282 2.255 2.226 2.186 2.153 2.143 2.120 2.091 2.056 2.044
F
N
Bn
O
N
H
2d
0.93
1.04
4.82
1.02
0.99
2.00
2.01
4.13
f1 (ppm)

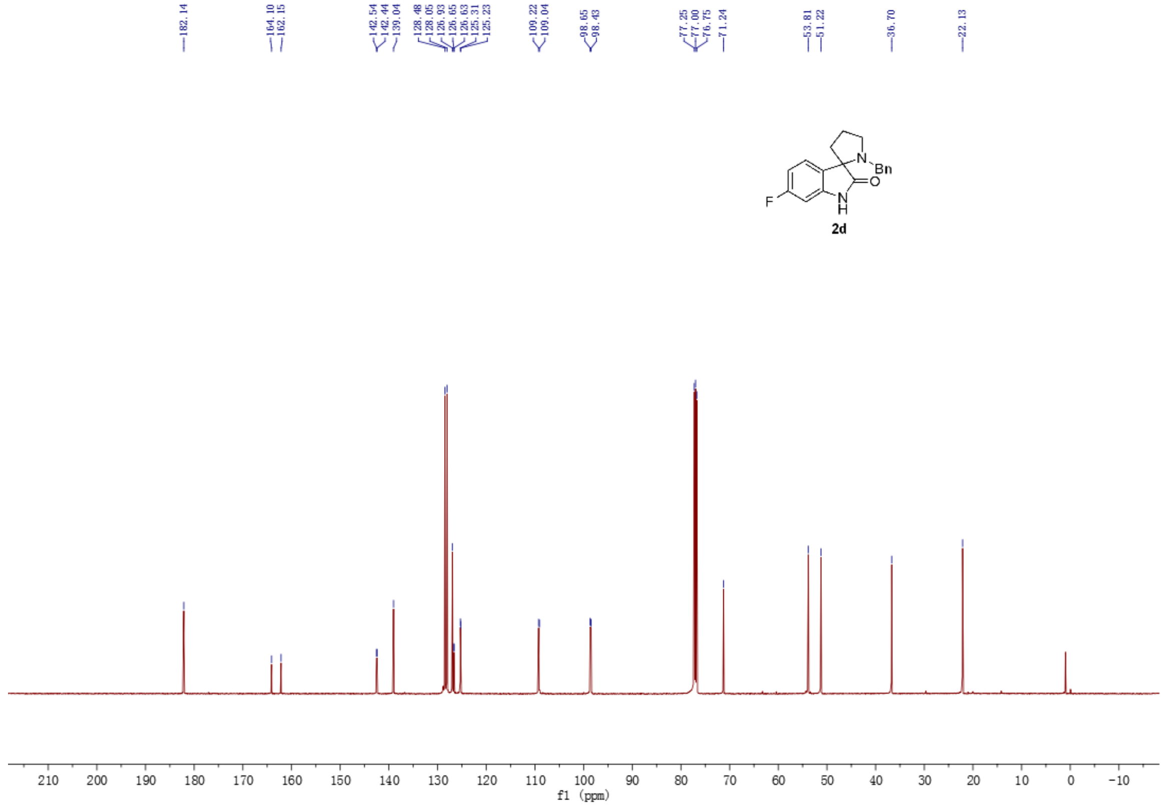
182.14
164.10 162.15
142.54 142.44 139.04
128.48 128.05 126.93 126.65 126.63 125.31 125.23
109.22 109.04
98.65 98.43
77.25 77.00 76.75
71.24
53.81 51.22
36.70
22.13
F
N
Bn
O
N
H
2d
f1 (ppm)

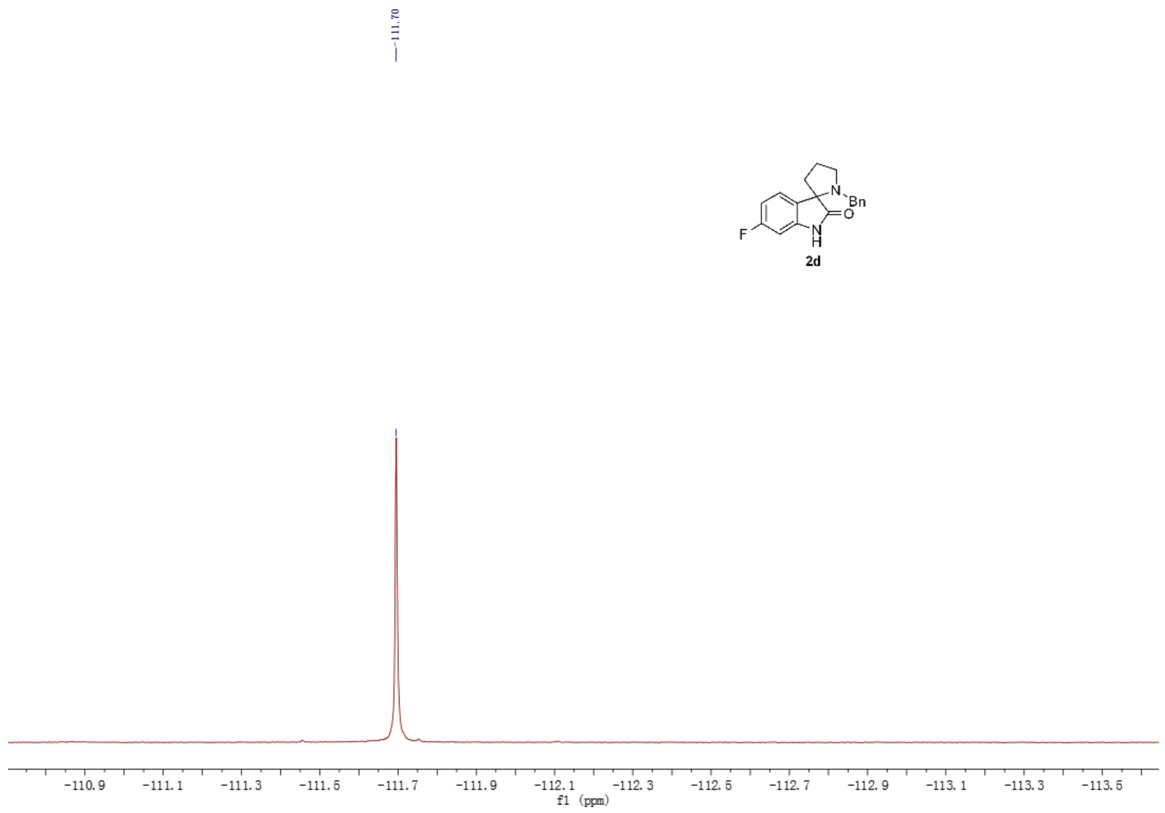
-111.70
2d
f1 (ppm)

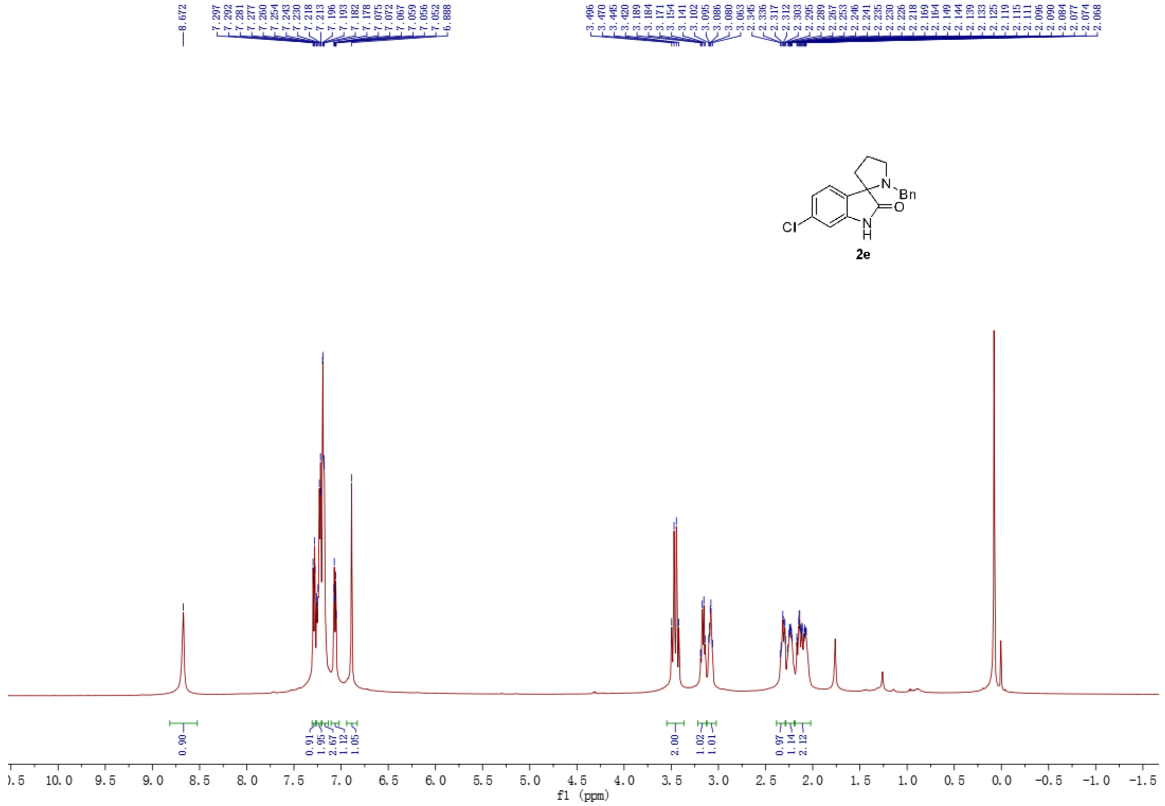
2e
f1 (ppm)

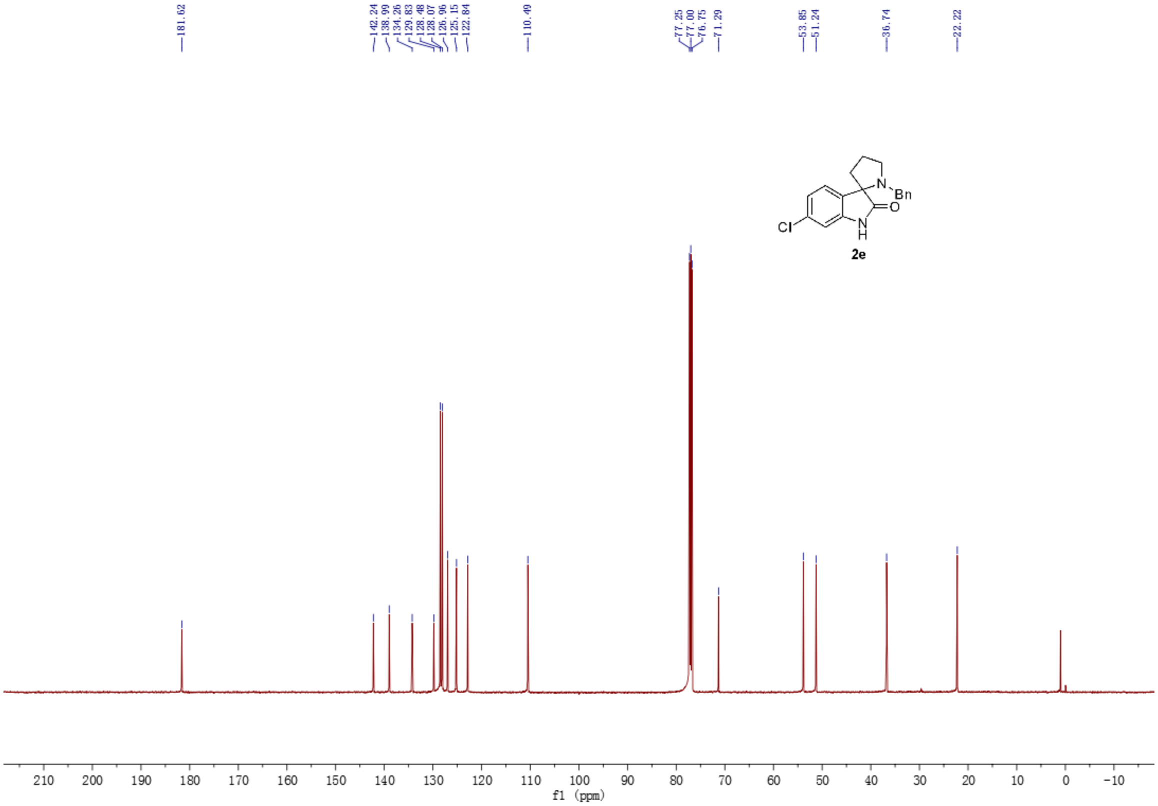
181.62
142.24
138.99
134.26
129.83
128.48
128.07
126.96
125.15
122.84
110.49
77.25
77.00
76.75
71.29
53.85
51.24
36.74
22.22
N
Bn
O
Cl
N
H
2e
210 200 190 180 170 160 150 140 130 120 110 100 90 80 70 60 50 40 30 20 10 0 -10
f1 (ppm)

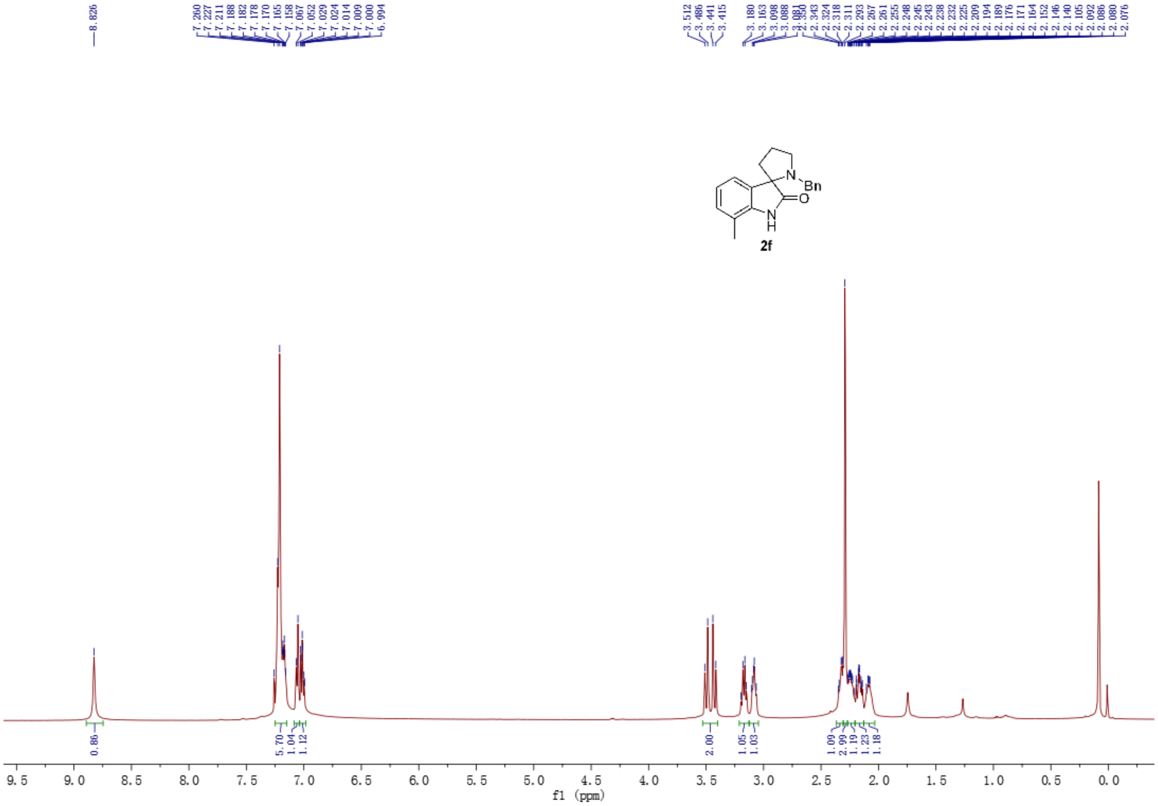
8.826
7.260
7.227
7.211
7.188
7.182
7.178
7.170
7.165
7.158
7.067
7.052
7.029
7.024
7.014
7.009
7.000
6.994
3.512
3.486
3.441
3.415
3.180
3.163
3.098
3.088
2.343
2.324
2.318
2.311
2.293
2.267
2.261
2.255
2.248
2.245
2.243
2.238
2.232
2.225
2.209
2.194
2.189
2.176
2.171
2.164
2.152
2.146
2.140
2.105
2.092
2.086
2.080
2.076
N
Bn
O
N
H
2f
0.86
5.70
1.04
1.12
2.00
1.05
1.03
1.09
2.99
1.19
1.23
1.18
9.5 9.0 8.5 8.0 7.5 7.0 6.5 6.0 5.5 5.0 4.5 4.0 3.5 3.0 2.5 2.0 1.5 1.0 0.5 0.0
f1 (ppm)

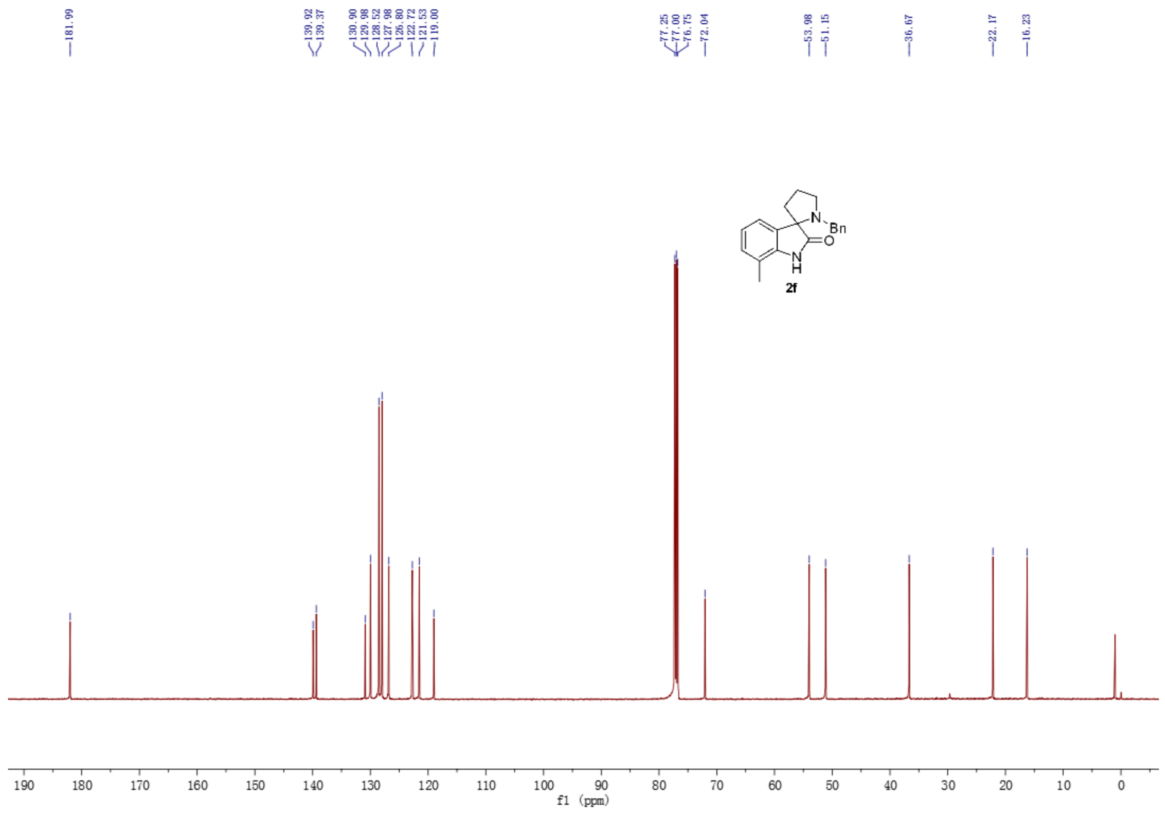
2f
f1 (ppm)

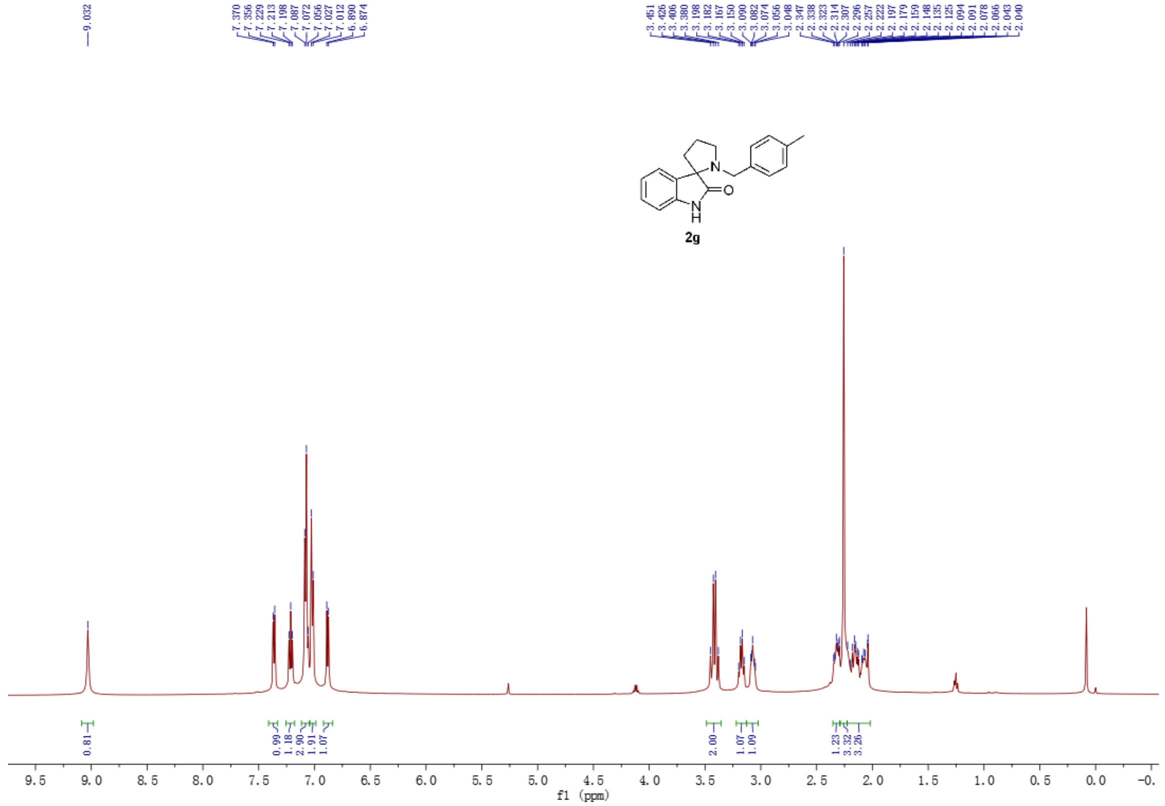
2g
f1 (ppm)

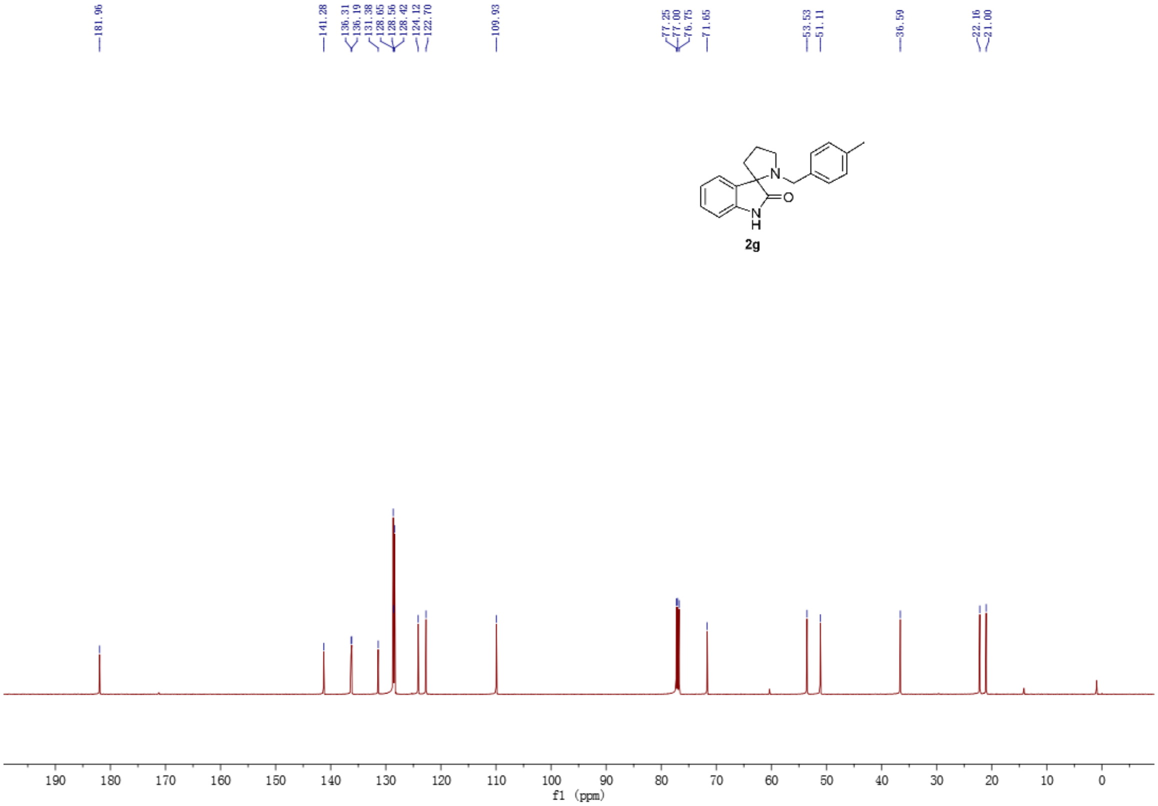
181.96
141.28
136.31
136.19
131.38
128.65
128.56
128.42
124.12
122.70
109.93
77.25
77.00
76.75
71.65
53.53
51.11
36.59
22.16
21.00
2g
190 180 170 160 150 140 130 120 110 100 90 80 70 60 50 40 30 20 10 0
f1 (ppm)

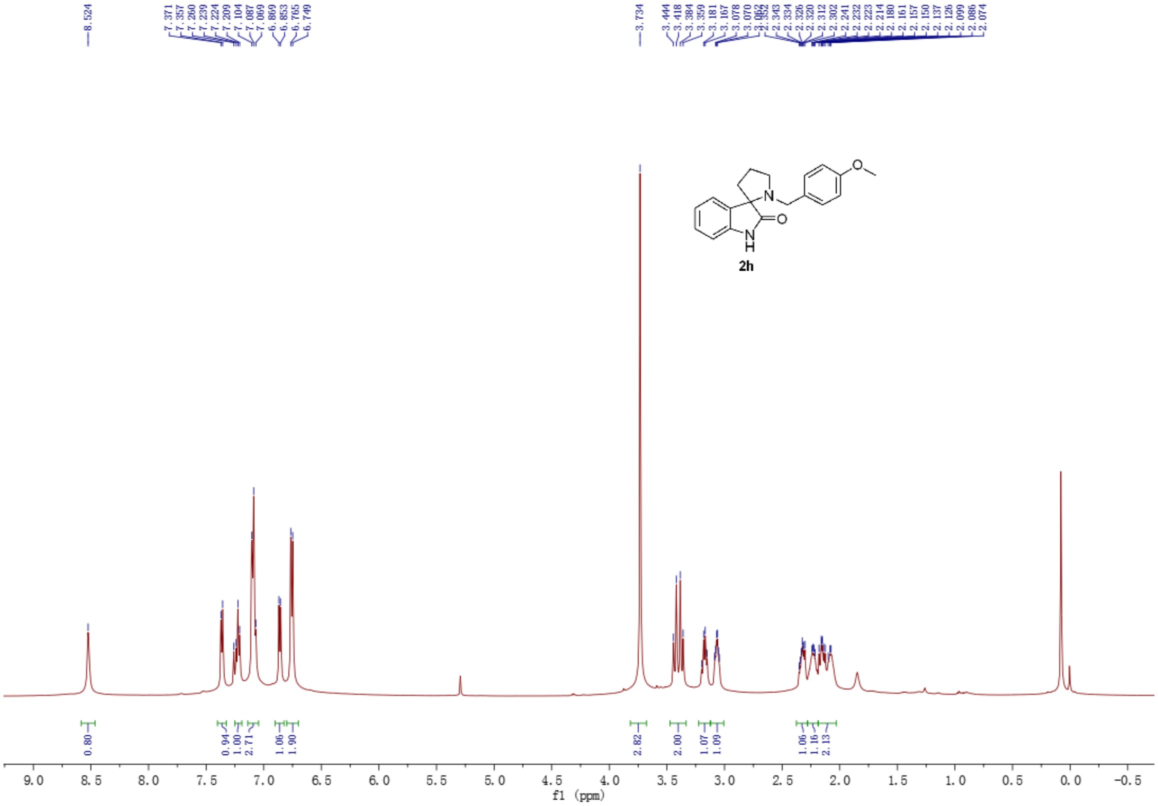
8.524
7.371
7.357
7.260
7.239
7.224
7.209
7.104
7.087
7.069
6.869
6.853
6.765
6.749
3.734
3.444
3.418
3.384
3.359
3.181
3.167
3.078
3.070
2.343
2.334
2.326
2.320
2.312
2.302
2.241
2.232
2.223
2.214
2.180
2.161
2.157
2.150
2.137
2.126
2.099
2.086
2.074
2h
0.80
0.94
1.00
2.71
1.06
1.90
2.82
2.00
1.07
1.09
1.06
1.16
2.13
9.0 8.5 8.0 7.5 7.0 6.5 6.0 5.5 5.0 4.5 4.0 3.5 3.0 2.5 2.0 1.5 1.0 0.5 0.0 -0.5
f1 (ppm)

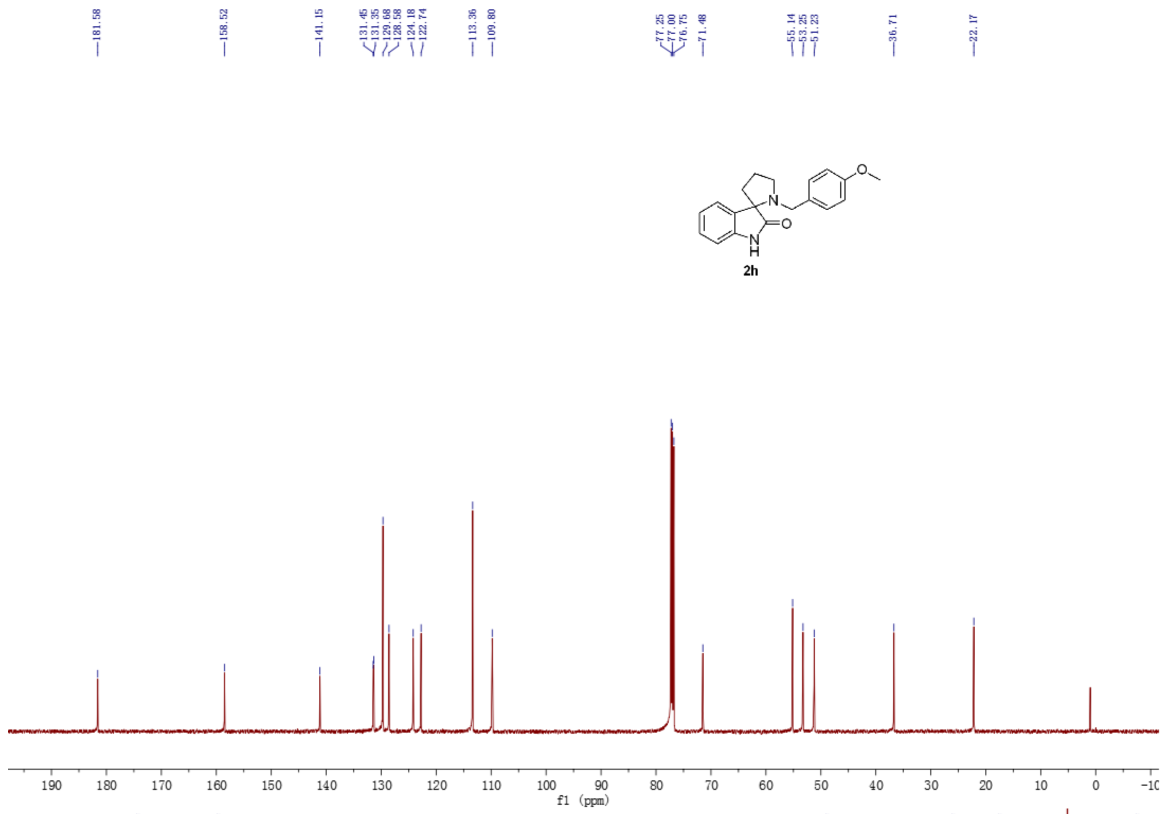
181.58
158.52
141.15
131.45
131.35
129.68
128.58
124.18
122.74
113.36
109.80
77.25
77.00
76.75
71.48
55.14
53.25
51.23
36.71
22.17
N
O
H
2h
f1 (ppm)

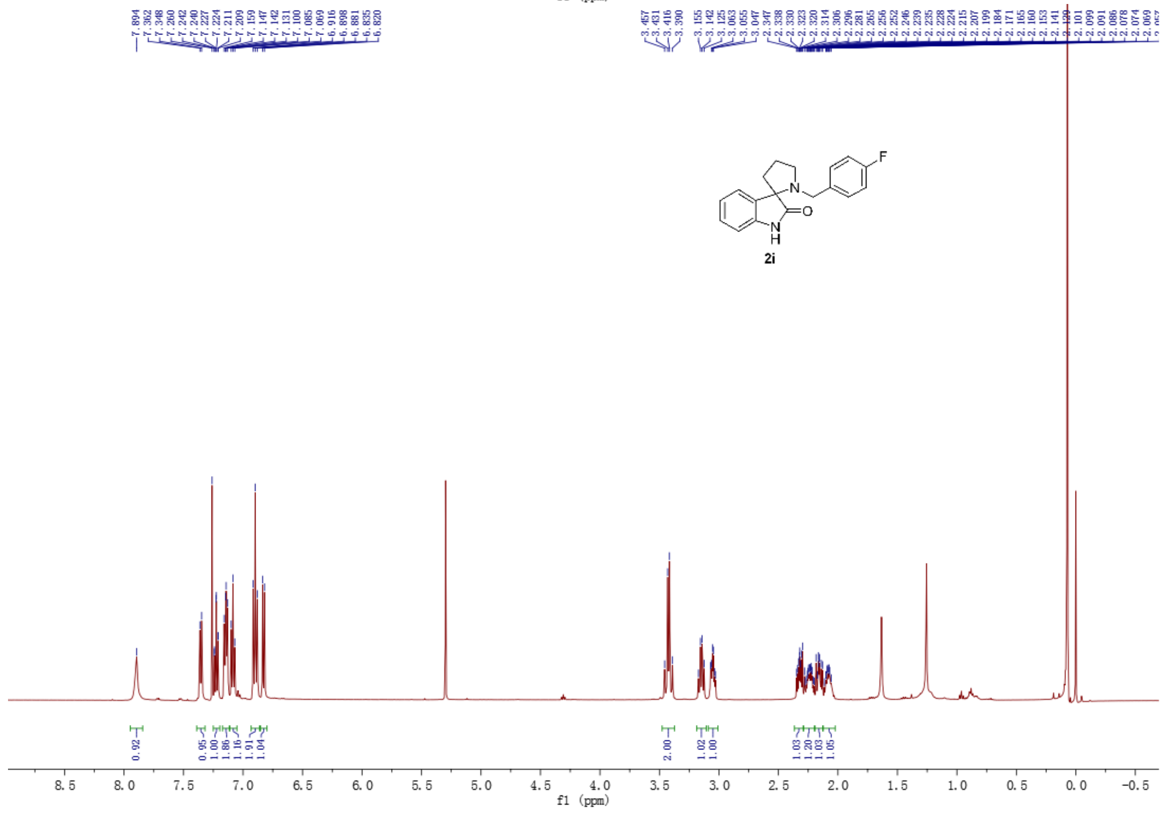
F
N
O
H
2i
0.92
0.95
1.00
1.86
1.16
1.91
1.04
2.00
1.02
1.00
1.03
1.20
1.03
1.05
f1 (ppm)

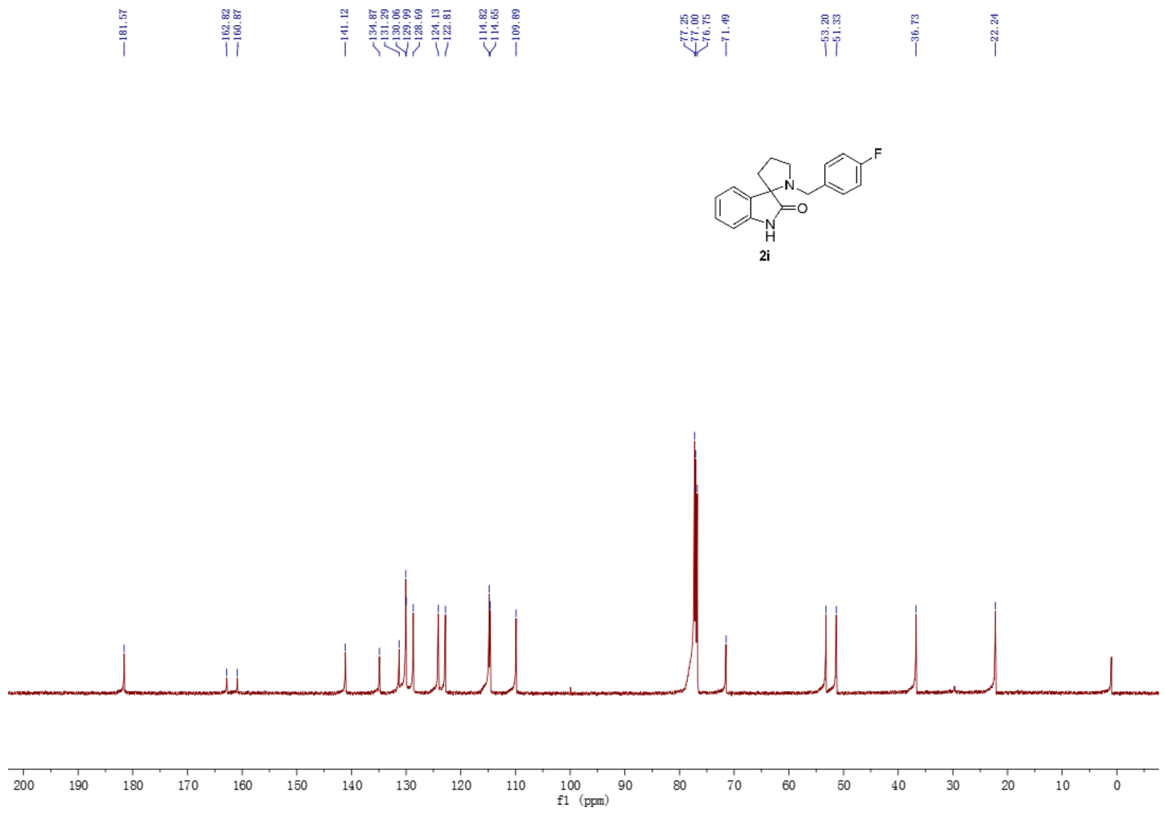
181.57
162.82
160.87
141.12
134.87
131.29
130.06
129.99
128.69
124.13
122.81
114.82
114.65
109.89
77.25
77.00
76.75
71.49
53.20
51.33
36.73
22.24
N
F
O
N
H
2i
f1 (ppm)

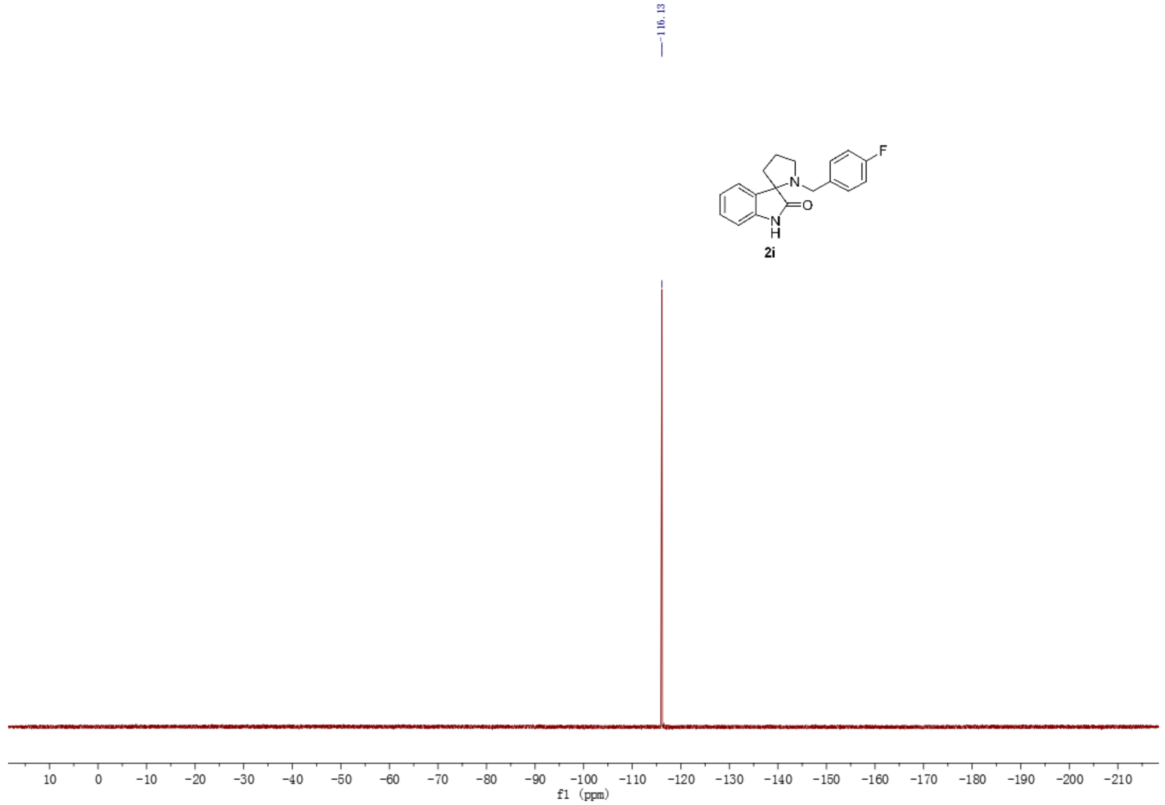
-116.13
N
F
O
N
H
2i
f1 (ppm)

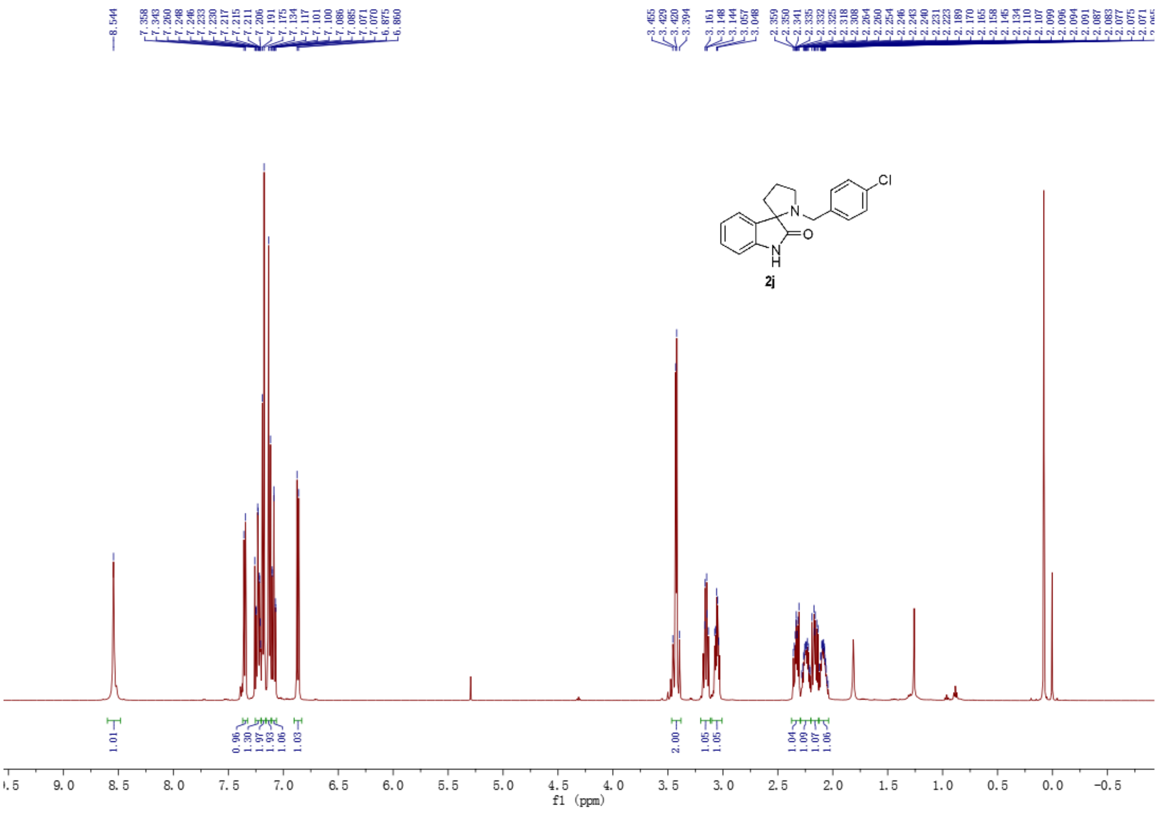

N
O
N
H
Cl
2j
f1 (ppm)

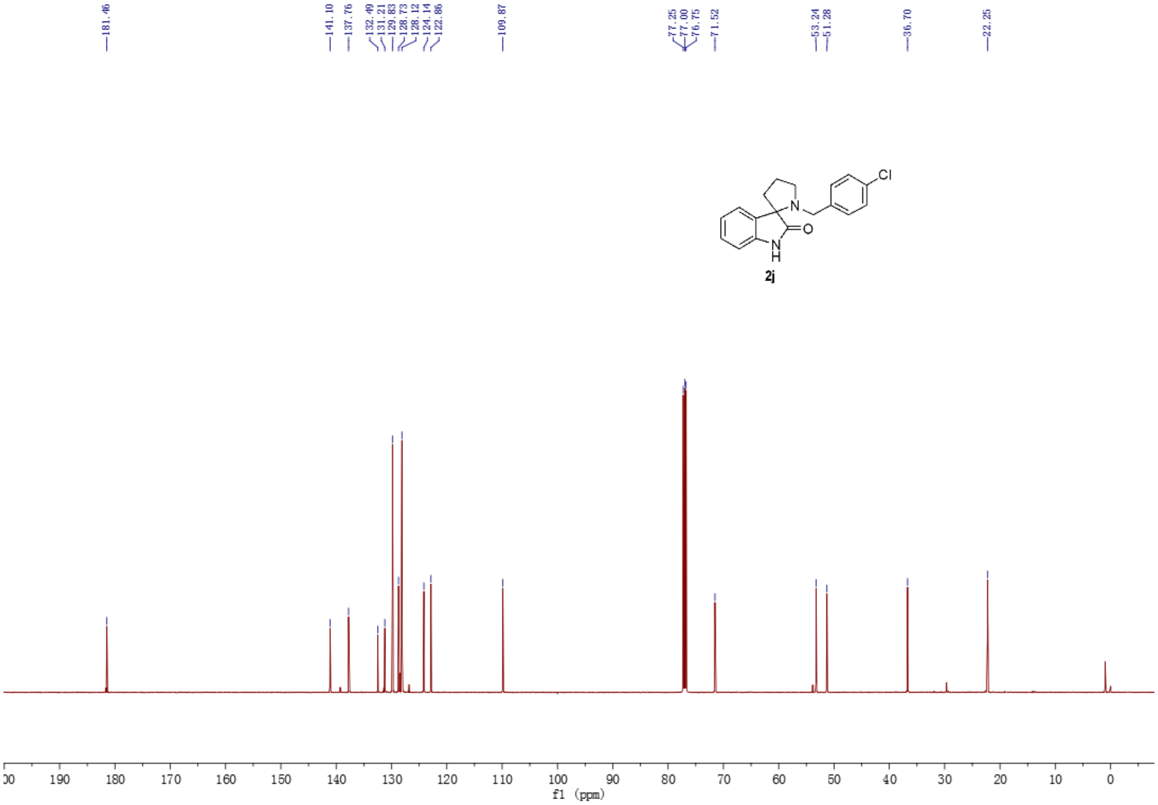

N
O
N
H
Cl
2j
f1 (ppm)

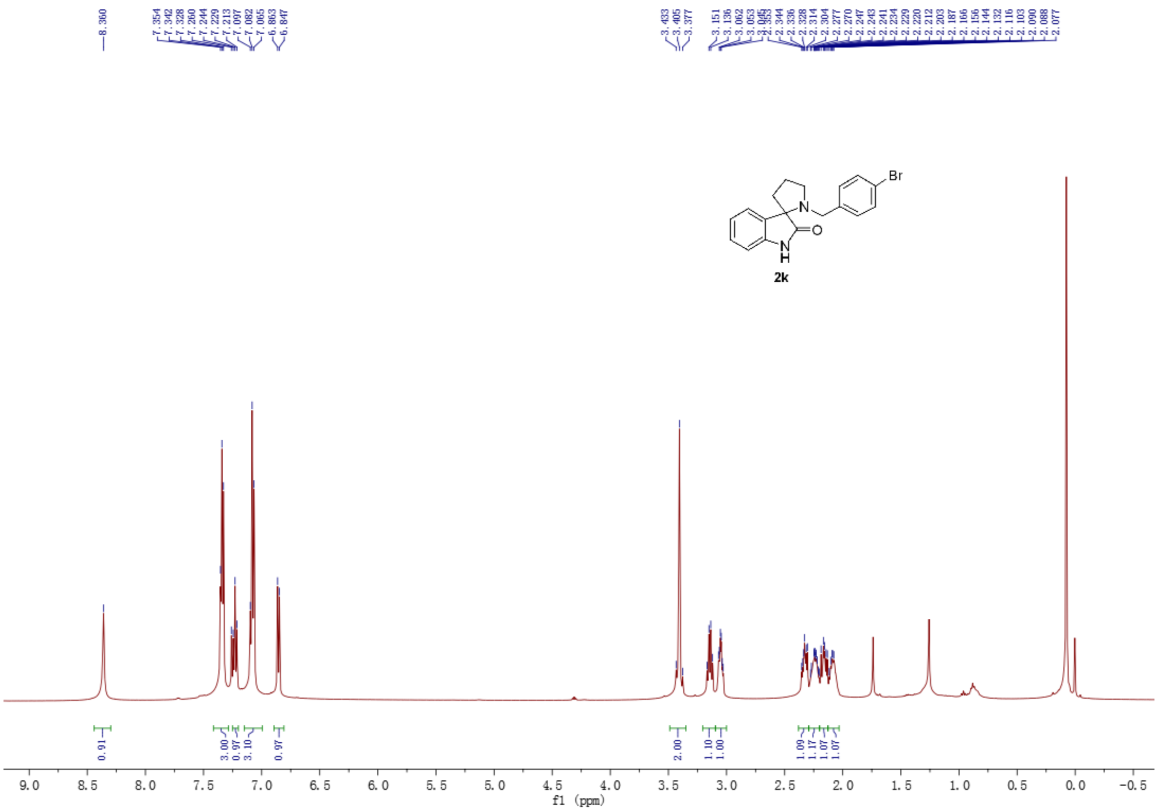
8.360
7.354
7.342
7.328
7.260
7.244
7.229
7.213
7.097
7.082
7.065
6.863
6.847
3.433
3.405
3.377
3.151
3.136
Br
N
O
N
H
2k
0.91
3.00
0.97
3.10
0.97
2.00
1.10
1.00
1.09
1.17
1.07
1.07
9.0
8.5
8.0
7.5
7.0
6.5
6.0
5.5
5.0
4.5
4.0
3.5
3.0
2.5
2.0
1.5
1.0
0.5
0.0
-0.5
f1 (ppm)

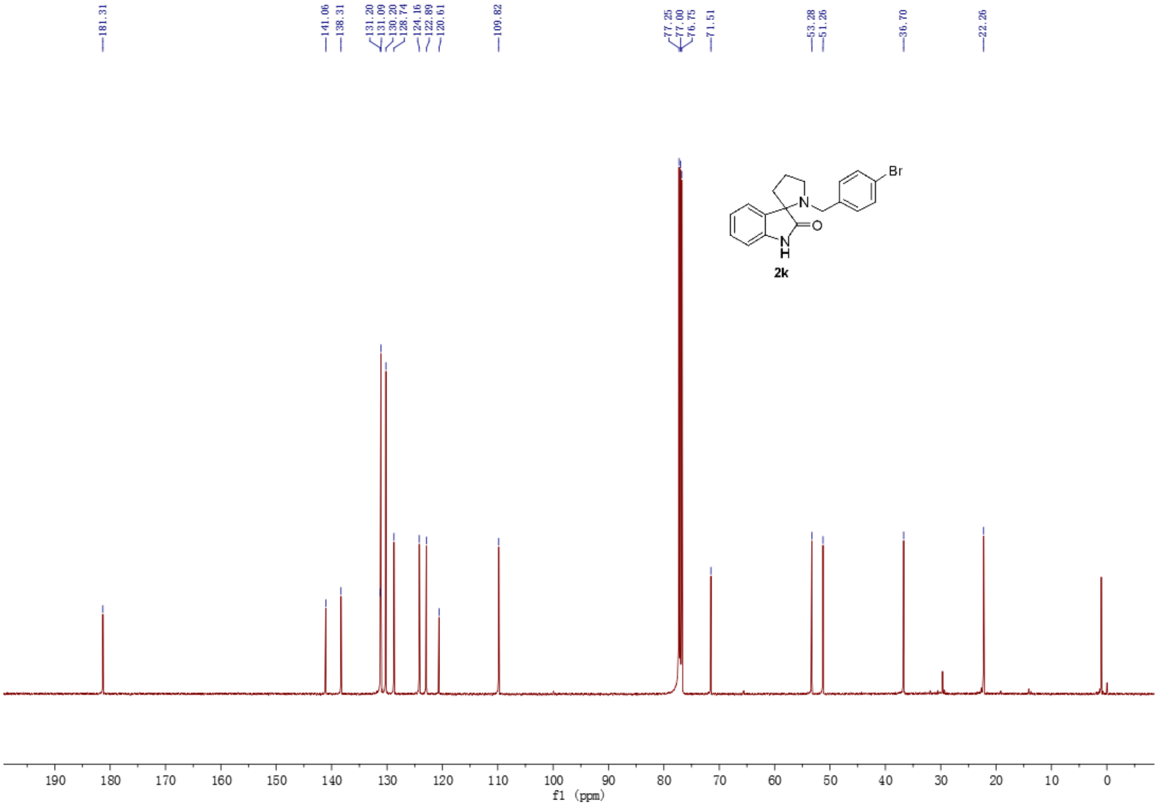
181.31
141.06
138.31
131.20
131.09
130.20
128.74
124.16
122.89
120.61
109.82
77.25
77.00
76.75
71.51
53.28
51.26
36.70
22.26
Br
N
O
N
H
2k
190
180
170
160
150
140
130
120
110
100
90
80
70
60
50
40
30
20
10
0
f1 (ppm)

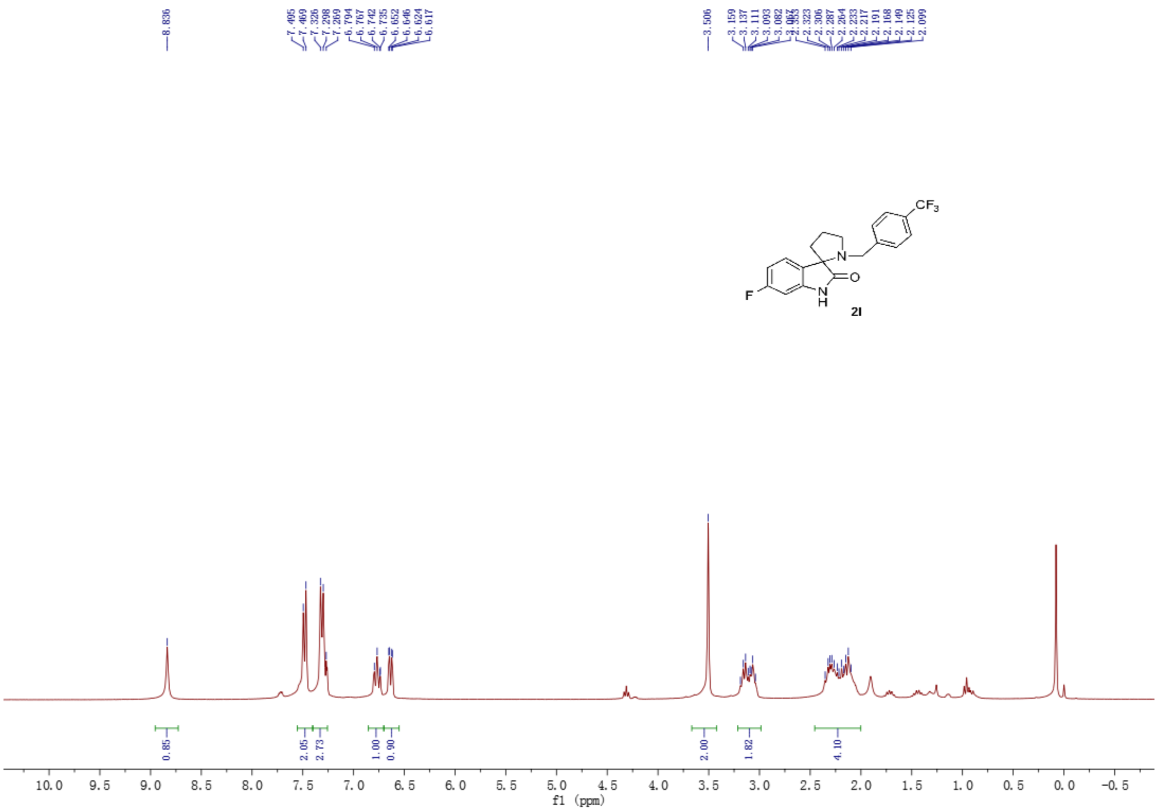
CF3
N
O
F
H
2l
f1 (ppm)

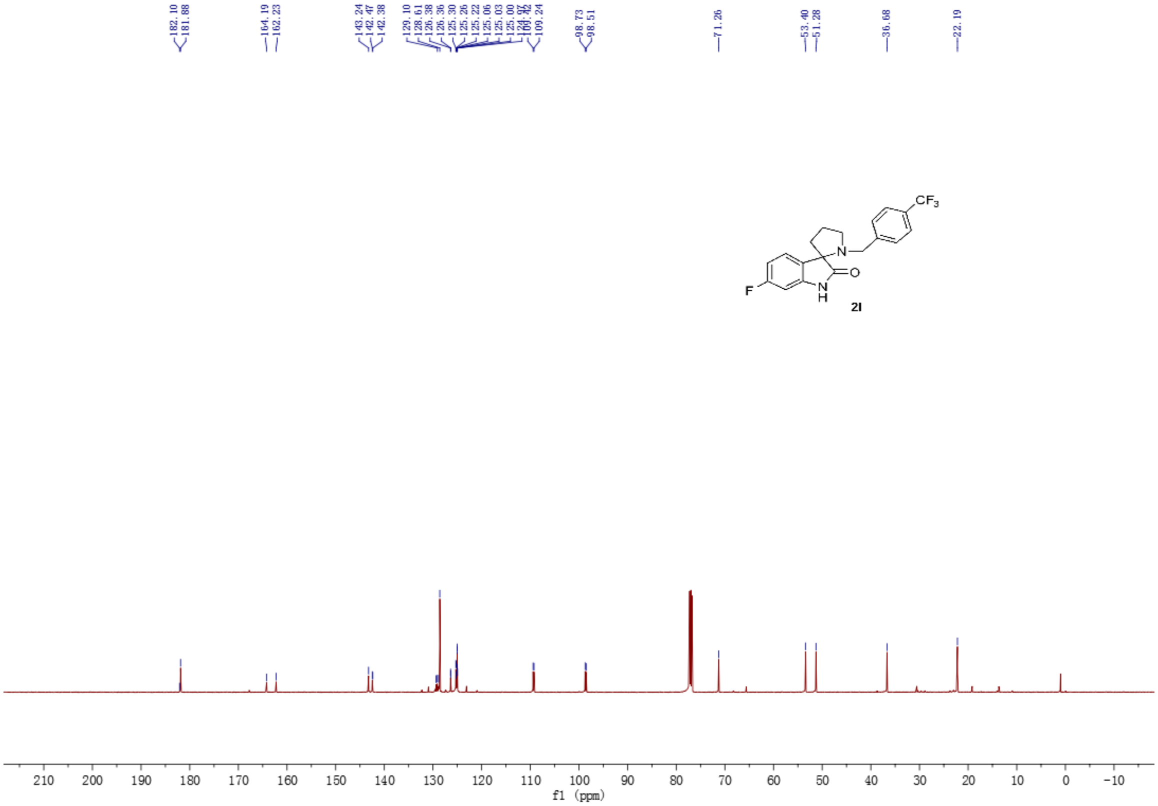
CF3
N
O
F
H
2l
f1 (ppm)

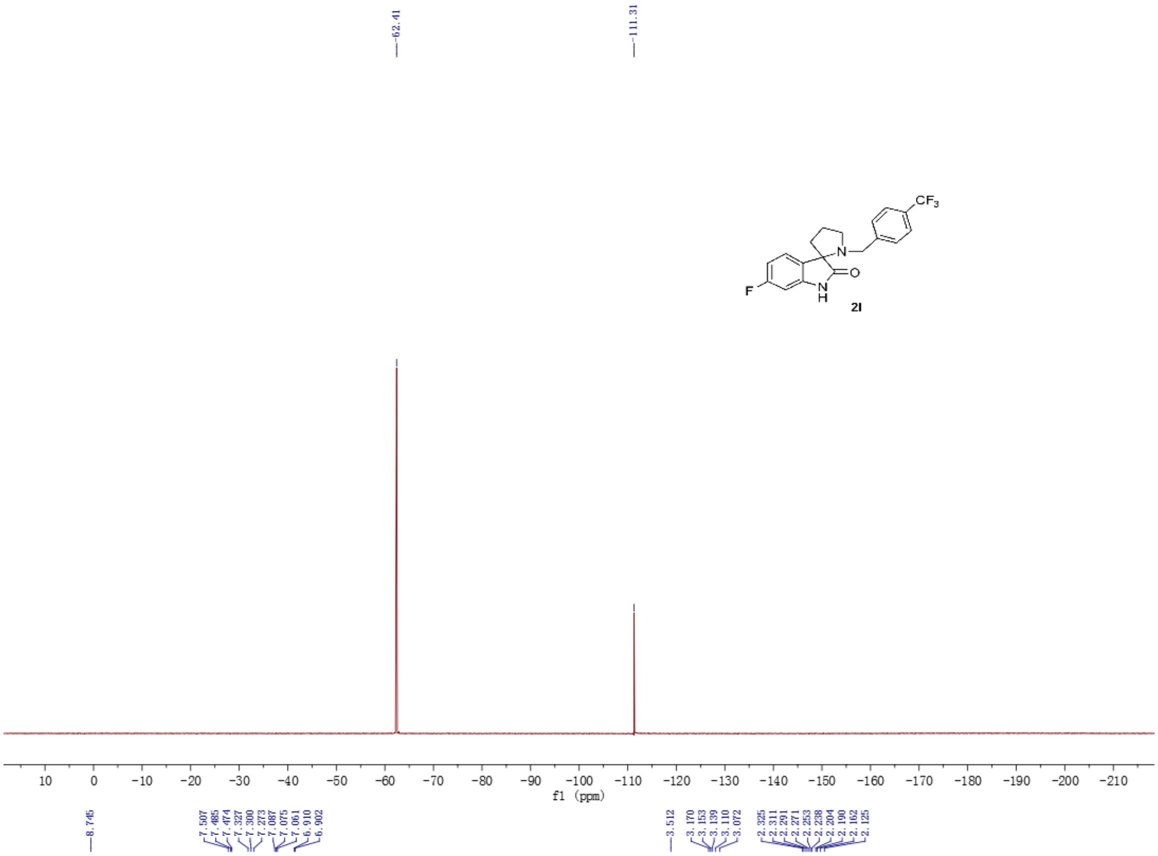

-62.41
-111.31
CF3
N
F
O
NH
2l
f1 (ppm)

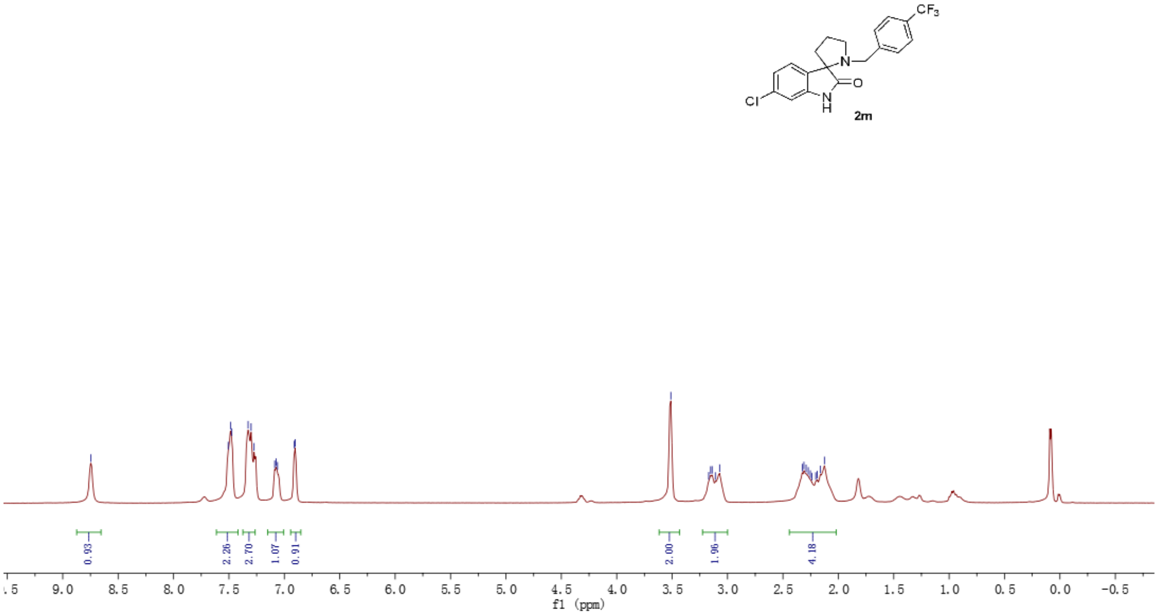

8.745
7.507 7.485 7.474 7.327 7.300 7.273 7.087 7.075 7.061 6.910 6.902
3.512
3.170 3.153 3.139 3.110 3.072
2.325 2.311 2.291 2.271 2.253 2.238 2.204 2.190 2.162 2.125
CF3
N
Cl
O
NH
2m
0.93 2.26 2.70 1.07 0.91 2.00 1.96 4.18
f1 (ppm)

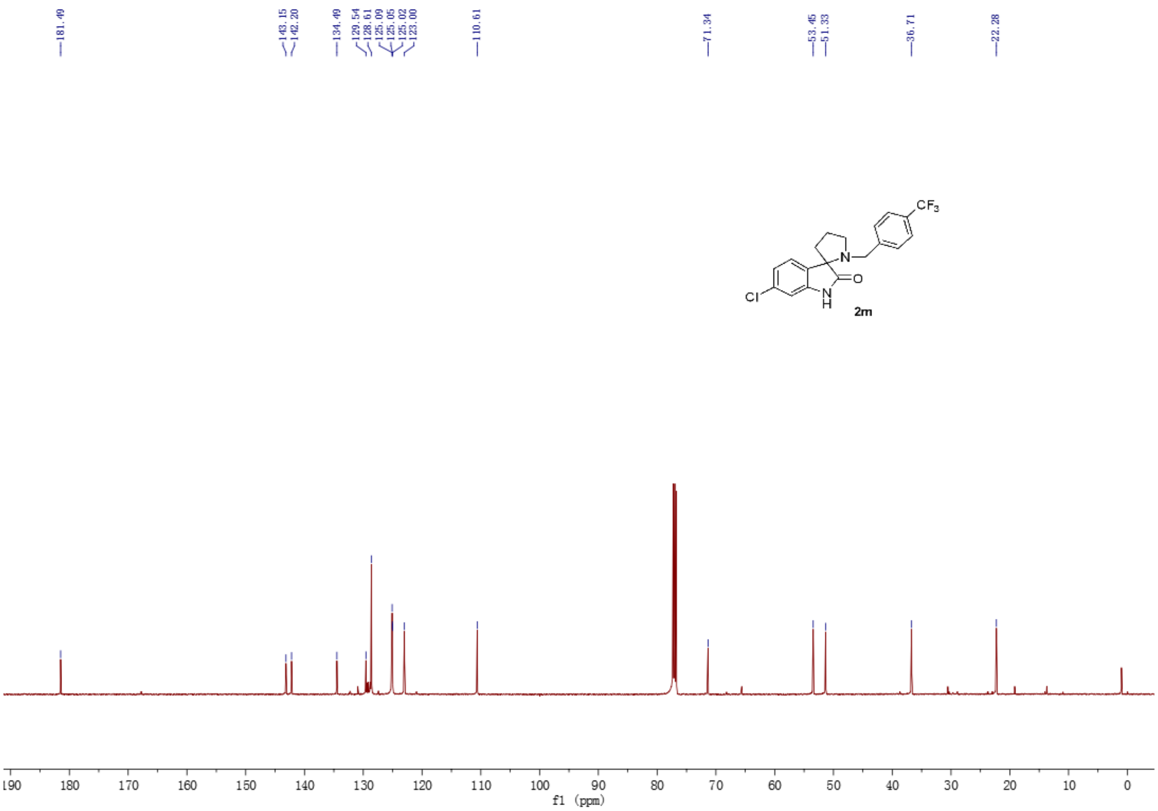
181.49
143.15
142.20
134.49
129.54
128.61
125.09
125.05
125.02
123.00
110.61
71.34
53.45
51.33
36.71
22.28
CF3
N
O
Cl
NH
2m
f1 (ppm)

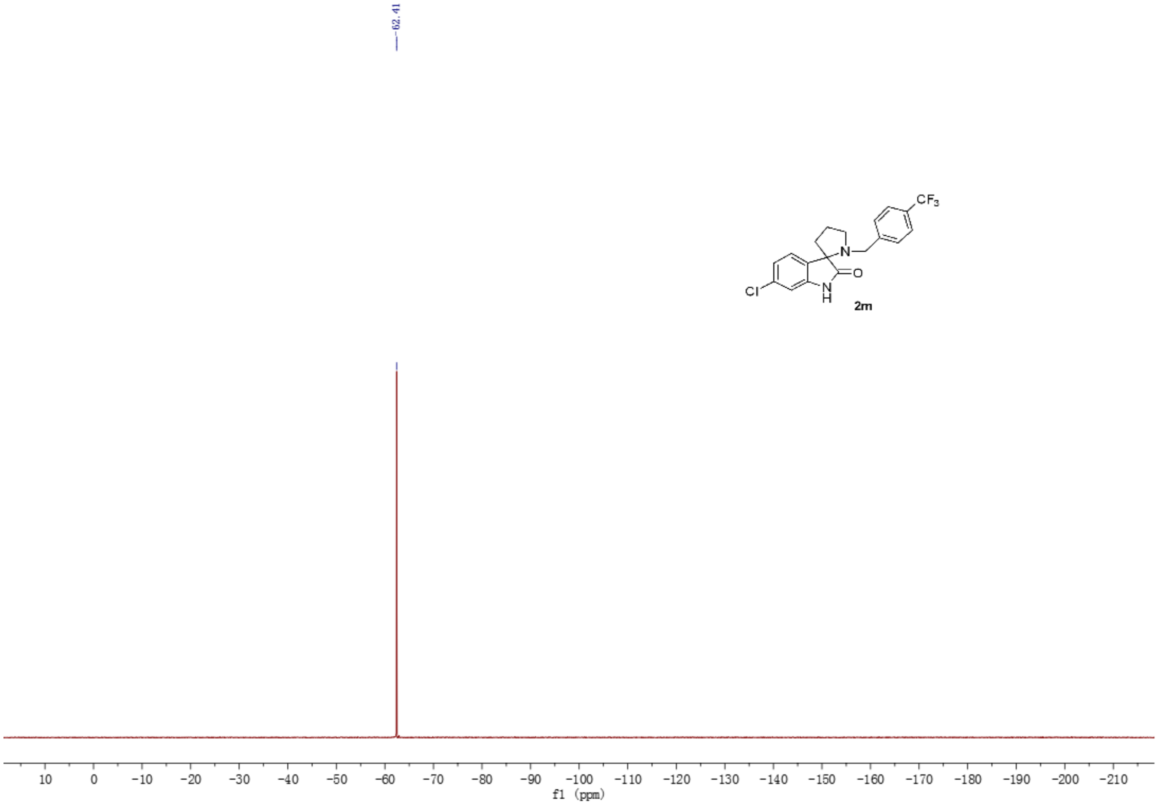
-62.41
CF3
N
O
Cl
NH
2m
f1 (ppm)

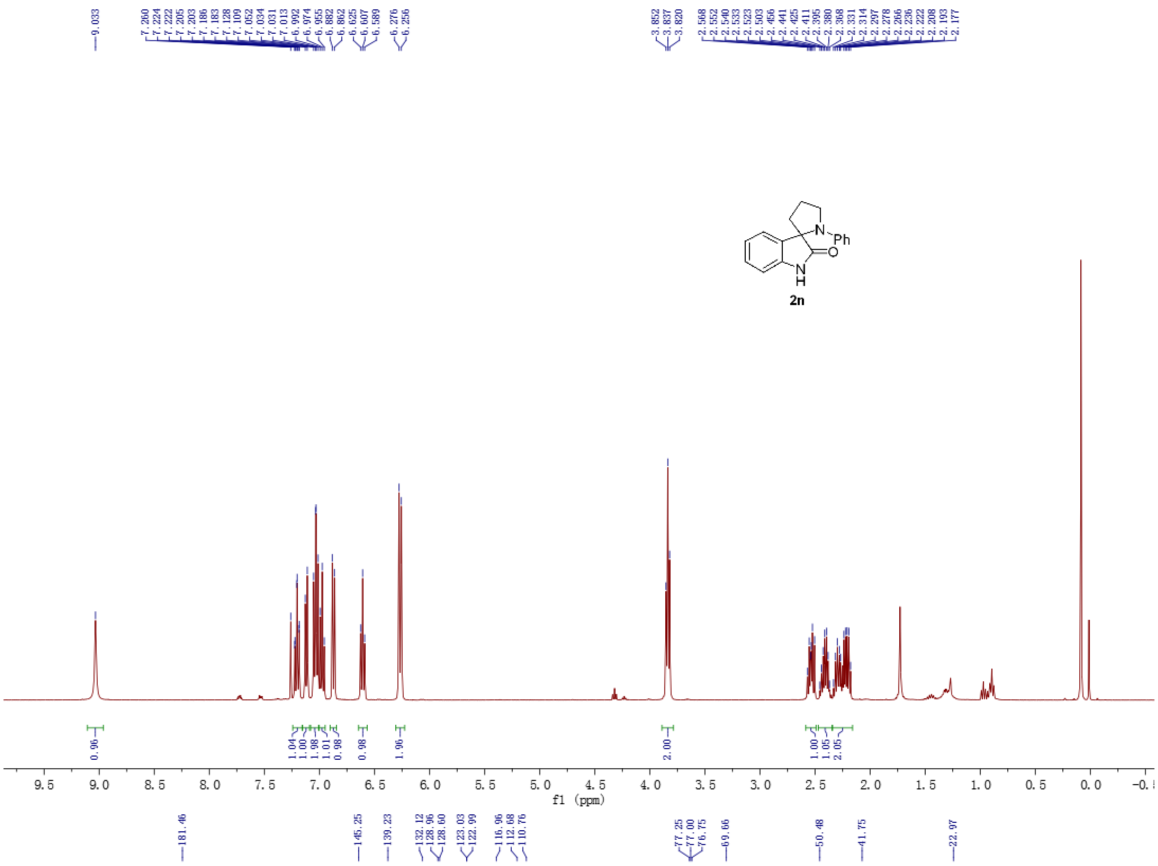
N
Ph
O
N
H
2n
f1 (ppm)

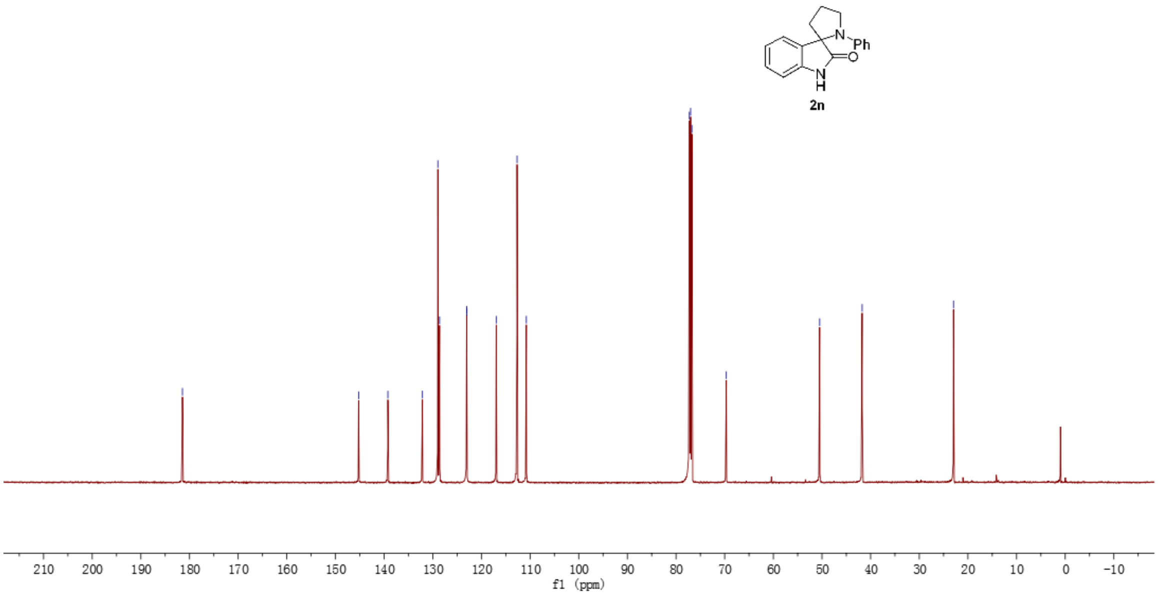
N
Ph
O
N
H
2n
f1 (ppm)

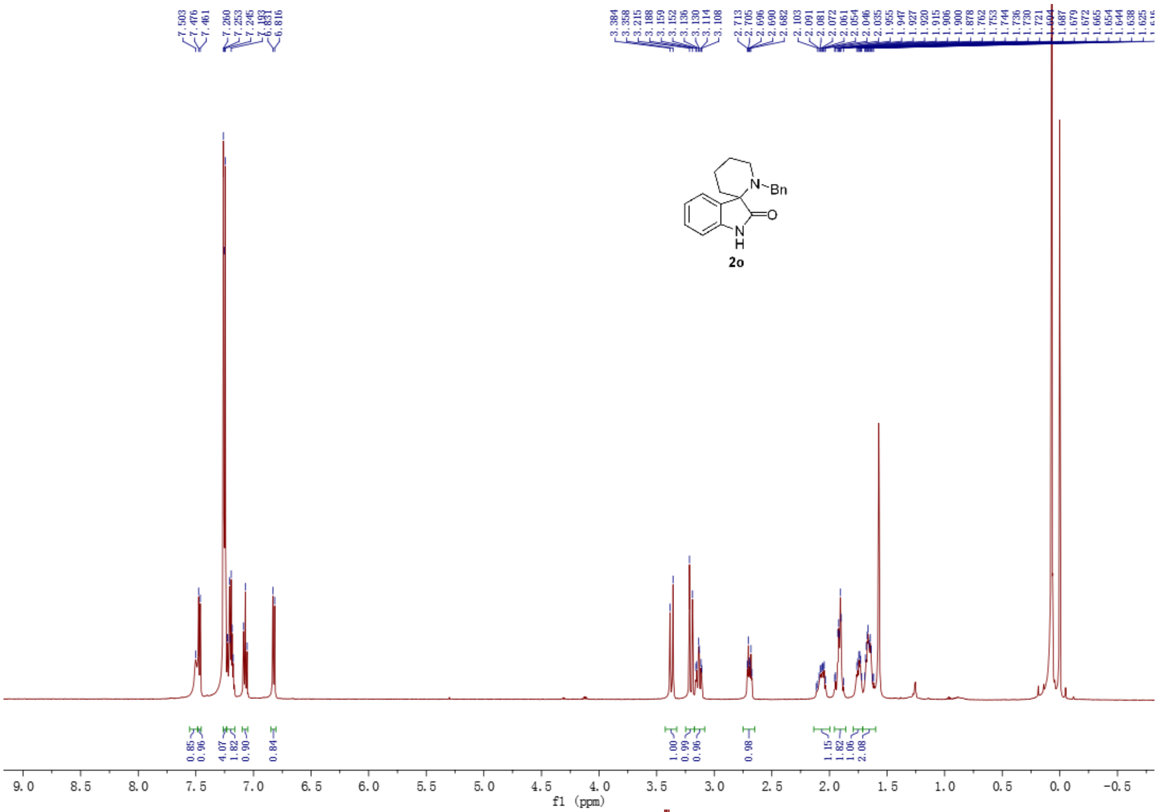
N
Bn
O
H
2o
f1 (ppm)

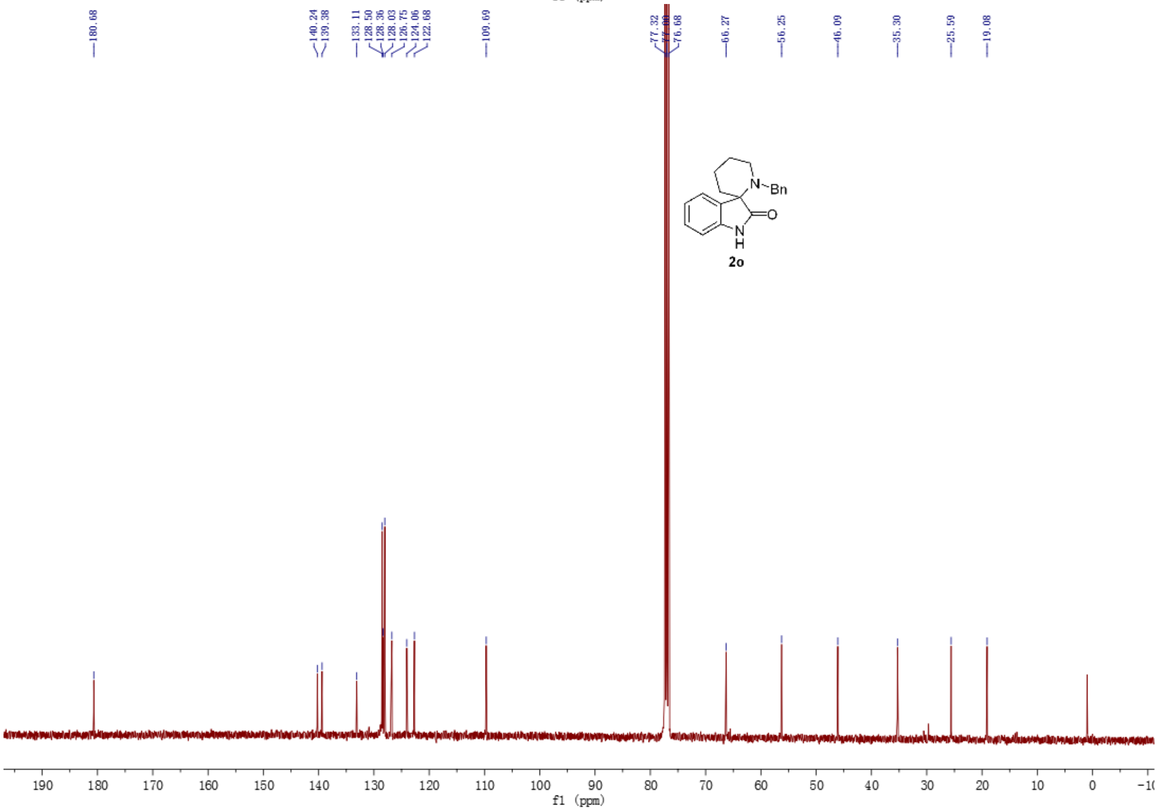
180.68
140.24
139.38
133.11
128.50
128.36
128.03
126.75
124.06
122.68
109.69
77.32
76.68
66.27
56.25
46.09
35.30
25.59
19.08
N
Bn
O
H
2o
f1 (ppm)

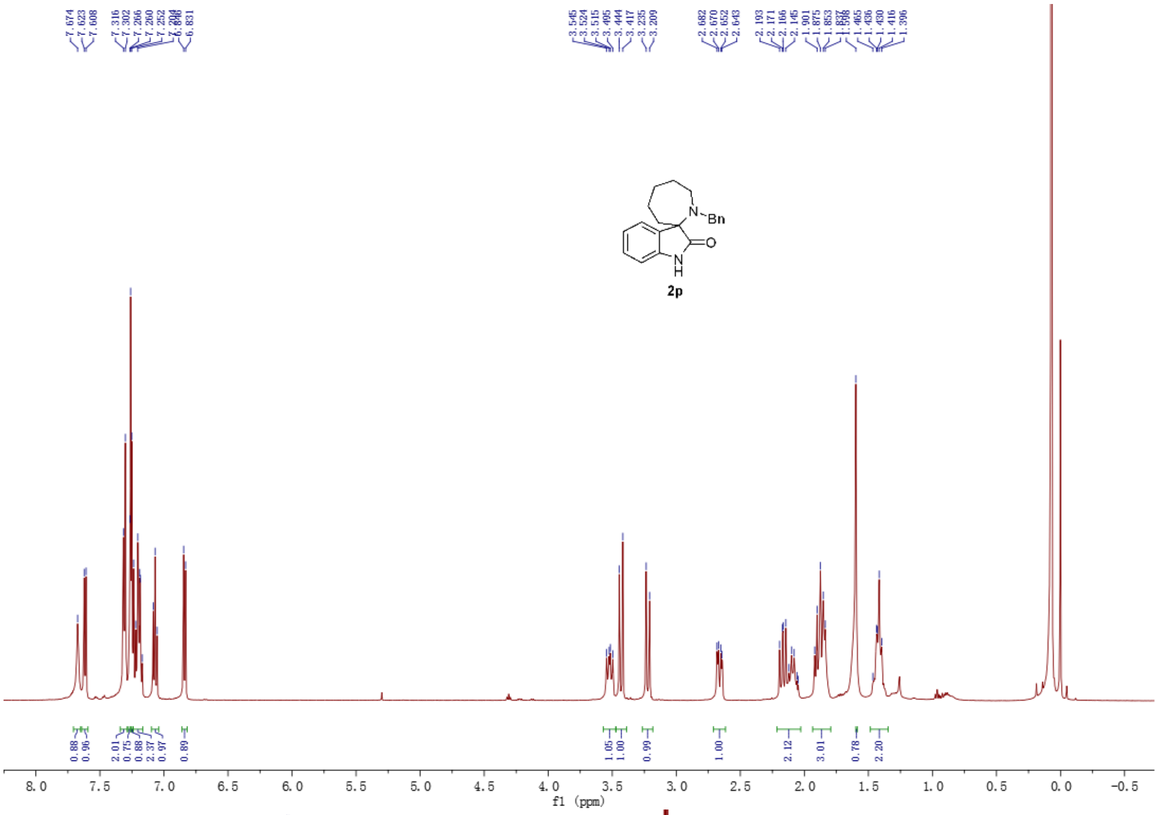
Bn
2p
f1 (ppm)

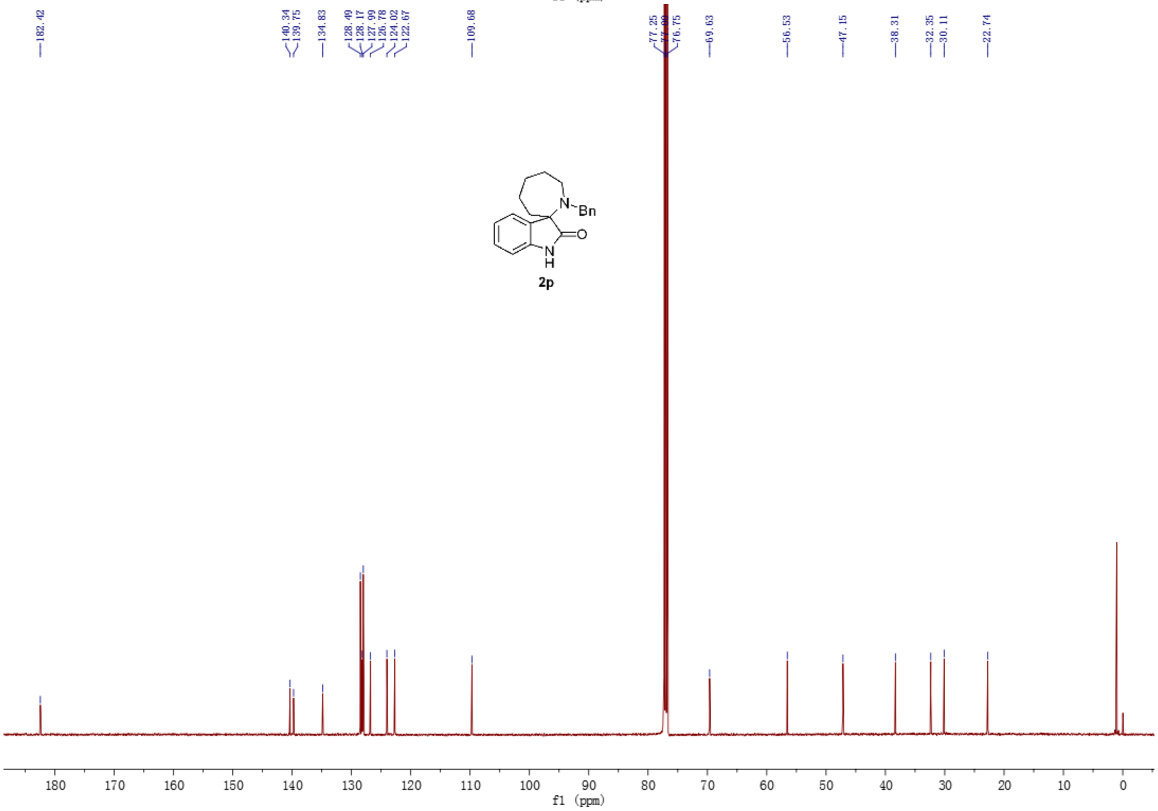
Bn
2p
f1 (ppm)

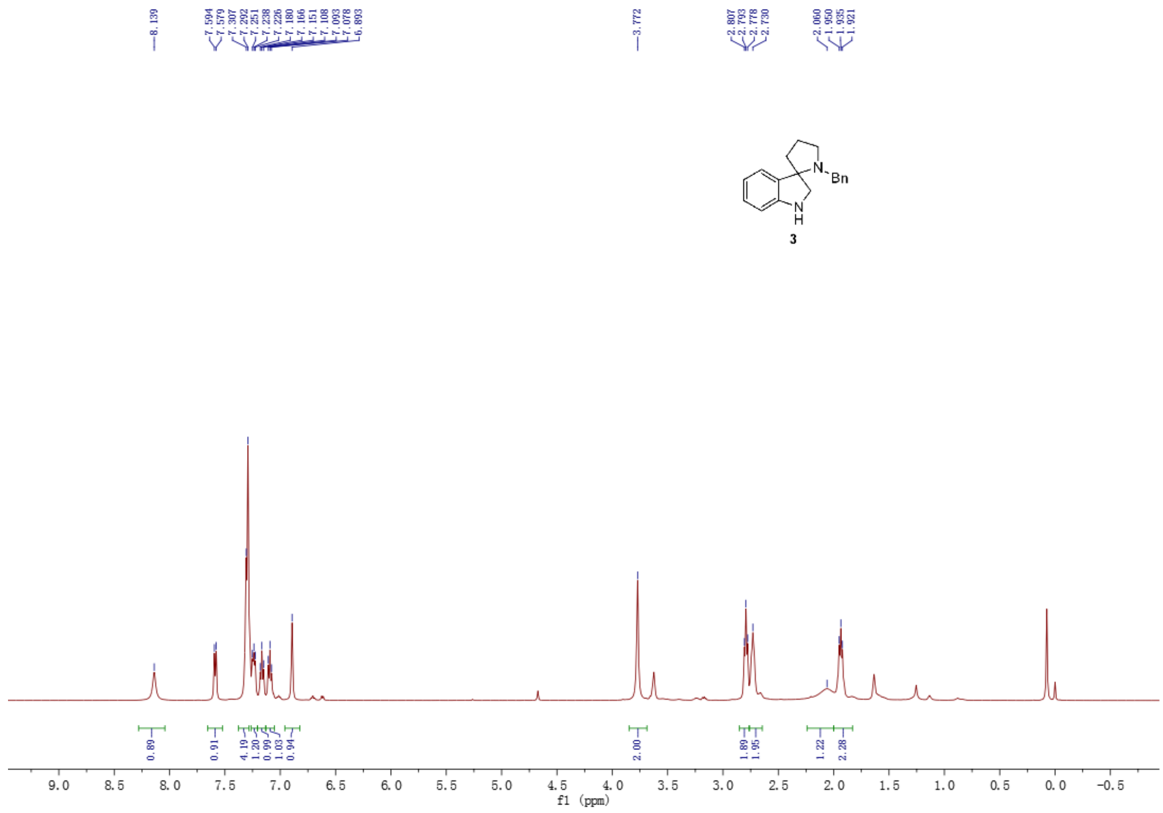
8.139
7.594 7.579 7.307 7.292 7.251 7.238 7.226 7.180 7.166 7.151 7.108 7.093 7.078 6.893
3.772
2.807 2.793 2.778 2.730
2.060 1.950 1.935 1.921
N
Bn
H
3
0.89 0.91 4.19 1.20 0.99 1.03 0.94 2.00 1.89 1.95 1.22 2.28
9.0 8.5 8.0 7.5 7.0 6.5 6.0 5.5 5.0 4.5 4.0 3.5 3.0 2.5 2.0 1.5 1.0 0.5 0.0 -0.5
f1 (ppm)

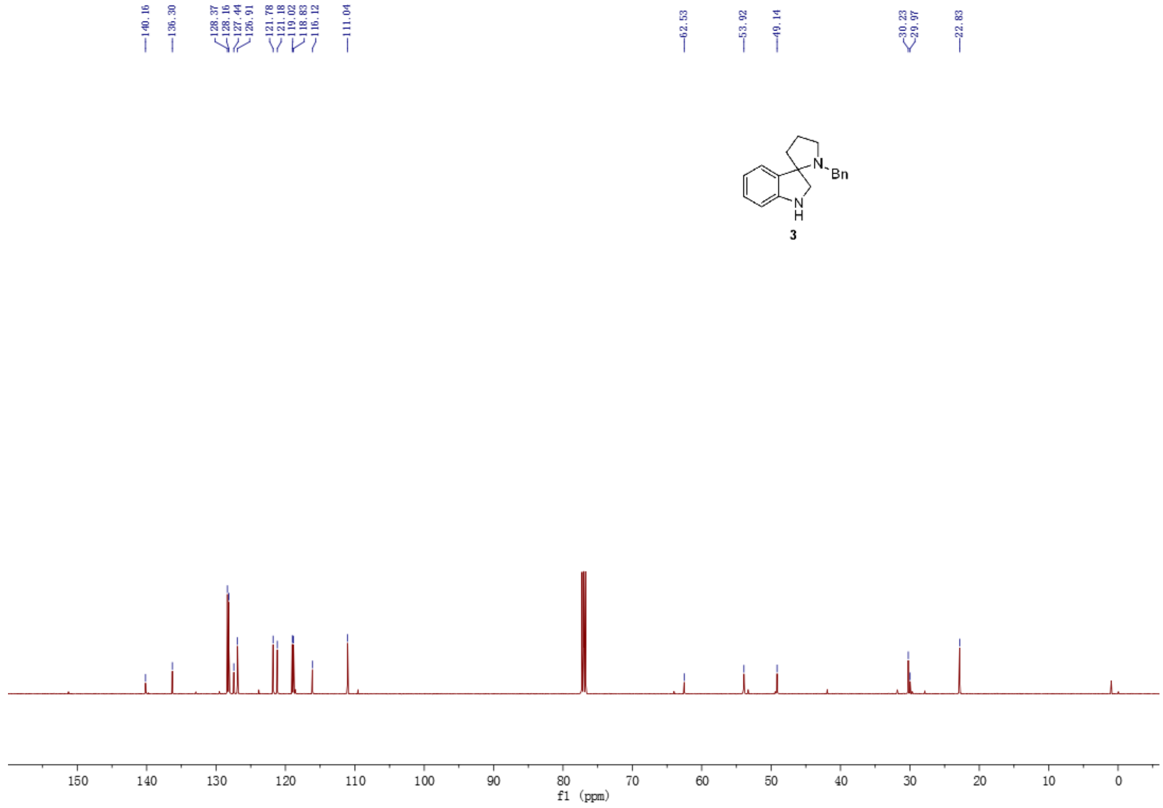
140.16
136.30
128.37 128.16 127.44 126.91
121.78 121.18 119.02 118.83 116.12
111.04
62.53
53.92
49.14
30.23 29.97
22.83
N
Bn
H
3
150 140 130 120 110 100 90 80 70 60 50 40 30 20 10 0
f1 (ppm)

References

[S1] Li, G.; Huang, L.; Xu, J.; Sun, W.; Xie, J.; Hong, L.; Wang, R. *Adv. Synth. Catal.* **2016**, *358*, 2873.

[S2] Martin, D. B. C.; Nguyen, L. Q.; Vanderwal, C. D. *J. Org. Chem.* **2012**, *77*, 17 .

[S3] Cashion, D.; Mortensen, D; Huang, D.-H.; Torres, E.; Parens, J.; Sapienza, J.; Hansen, J.; Leftheris, K.; Correa, M.; Delgado, M. M.; Raheja, R.; Papa, P.; Bahmanyar, S.; Hesge, S.; Norris, S.; Plantevin-Krentiskysky, V.; Calabrese, A. A.; Cathers, B. E.; Whitefield, B. W.; Bennett, B. "Substituted Diaminopyrimidyl Compouns, Compositions Thereof, and Methods of Treatment Therewith." WO 095679 A1, June 25, 2015.
